# Toll-like receptor 9 (TLR9) expression correlates with cell of origin and predicts clinical outcome in diffuse large B-cell lymphoma

**DOI:** 10.1186/s12885-025-14359-7

**Published:** 2025-05-28

**Authors:** Sulaf Abd Own, Ioanna Xagoraris, Konstantina Stathopoulou, Björn E. Wahlin, Weicheng Ren, Mehran Ghaderi, Qiang Pan-Hammarström, Birgitta Sander, Karin E. Smedby, Georgios Rassidakis

**Affiliations:** 1 Department of Medicine Solna, Division of Clinical Epidemiology, Karolinska Institutet, Stockholm, Sweden; 2 Department of Clinical Pathology and Cancer Diagnostics, Karolinska University Hospital, Stockholm, 141 86 Sweden; 3 Department of Oncology-Pathology, Karolinska Institutet, Stockholm, Sweden; 4 Department of Hematology, Karolinska University, Hospital Solna, Stockholm, Sweden; 5 Department of Medicine Huddinge, Division of Hematology, Karolinska Institutet, Stockholm, Sweden; 6 Department of Medical Biochemistry and Biophysics, Division of Immunology, Karolinska Institutet, Stockholm, Sweden; 7 Department of Laboratory Medicine, Division of Pathology, Karolinska Institutet and Karolinska University Hospital, Stockholm, 14186 Sweden

**Keywords:** TLR9, Biomarker, Prognosis, DLBCL

## Abstract

**Background:**

Biological insights beyond the cell-of-origin (COO) classification can support clinical management in diffuse large B-cell lymphoma (DLBCL). We investigated if Toll-like receptor 9 (TLR9) expression could serve as a prognostic marker in DLBCL.

**Method:**

*TLR9* gene expression was analysed in four publicly available cohorts (*n = *2474), and protein expression was investigated in germinal centre B-cell (GCB) and activated B-cell (ABC) DLBCL cell lines. Next, TLR9 protein expression was analysed in 120 diagnostic samples from R-CHOP-treated patients with relapsed/refractory disease (poor outcome, *n = *50) or in complete remission (good outcome, *n = *70). Associations were evaluated using logistic regression, estimating odds ratios (OR) and 95% confidence intervals (CI).

**Results:**

*TLR9* gene expression was higher in ABC DLBCL compared to GCB DLBCL in external cohorts, and similar results were obtained for protein expression in cell lines. In patient samples, high TLR9 protein expression correlated with non-GCB type (*p =* 0.003) and poor outcome (*p =* 0.0016). High TLR9 expression remained associated with poor outcome in multivariable analysis after adjusting for COO and other clinical features (OR = 3.36, 95% CI 1.41–8.04). In exploratory analyses, a decrease of cell growth in ABC cell lines following inhibition of TLR9 activity with ODN4084-F was suggested.

**Conclusion:**

We conclude that TLR9 correlates with ABC/non-GCB phenotype and is a potential predictor of poor prognosis in DLBCL.

**Supplementary Information:**

The online version contains supplementary material available at 10.1186/s12885-025-14359-7.

## Background

Diffuse large B-cell lymphoma (DLBCL) is the most common type of lymphoid neoplasm. Although DLBCL is an aggressive disease, it has diverse molecular characteristics and clinical outcome. Approximately 25% of the patients suffer from refractory disease or relapse after standard chemoimmunotherapy with anthracycline-based regimens [1]. Treatment of refractory disease and early relapse (within 12 months) remains a large clinical challenge. In daily practice, primary treatment decisions and risk stratifications are still largely based on the international prognostic index (IPI) score although knowledge and use of more refined biological prognostic and predictive markers has increased dramatically in recent years. In the era of next generation sequencing (NGS), genetic signatures and oncogenic pathway drivers have proven to be important predictors for DLBCL outcome [2–4]. However, NGS-based classification algorithms are not yet implemented in clinical practice.

In this study, we sought to investigate the clinical significance of Toll-like receptor 9 (TLR9) expression in DLBCL. The TLRs are a family of proteins that play a fundamental role in the innate immune system and 10 TLRs have been described in humans. They act as pattern recognition receptors recognizing specific molecular patterns often associated with pathogens like virus and bacteria. In a previous study, genes involved in the TLR pathway were more frequently mutated in patients who did not achieve complete remission following primary treatment of DLBCL [5]. TLR9 is the dominant TLR in B cells, and it is highly expressed in the dark and light zones subcomponent of the germinal center and TLR9 can, by binding to CpG DNA, drive B-cell proliferation and survival through NF-kB activation, a hallmark of activated B-cell (ABC) DLBCL. TLR9 signals primarily through MYD88, leading to the activation of downstream signaling cascades [6–8]. In MCD, a newly defined subtype of ABC DLBCL, MYD88, TLR9 and BCR signaling interact in a protein complex, the so-called the MY-T-BCR supercomplex, the presence of which may influence response to Bruton Tyrosine Kinase (BTK) inhibitors [9, 10]. Early clinical trials of TLR9 agonists have shown promising results in solid tumors such as ovarian and lung carcinoma, and in chronic lymphocytic lymphoma, pediatric acute lymphoblastic and acute myeloid leukemia [11–13]. In addition, TLR9 inhibition has been indicated as a possible therapeutic target in ABC DLBCL [14–16]. In view of the pluripotent role of TLR9 in DLBCL, especially of the ABC/non-germinal center B-cell (GCB) type, we investigated its potential as a prognostic marker in a population-based cohort of patients with good- and poor-prognosis DLBCL. We also performed *in-vitro* experiments to further substantiate our findings and explore its role as a future treatment target.

## Material and methods

### *TLR9* gene expression in external cohorts

We analysed gene expression in four external DLBCL cohorts with accession number phs001444 (*n = *521) [17], GSE117556 (*n = *723) [18], GSE181063 (*n = *810) [19] and GSE10846 (*n = *420) [20]. We initially analysed gene expression of all *TLRs* (*TLR1-10*) by cell of origin (COO) across all cohorts and subsequently focused on *TLR9*. We next analysed *TLR9* expression across various genetic subtypes [3] in the cohorts with available data. Additionally, we assessed the associations between *TLR9* expression and patients’ overall survival (OS) and progression-free survival (PFS) using the Kaplan–Meier method.

### Cell lines

We used four DLBCL cell lines (two GCB and two ABC) and two control cell lines of other lymphoma subtypes to assess TLR9 protein expression (Suppl. Table 1). More details are provided in the Supplementary methods.

### Study design and population

The study population was identified from a national population-based cohort of patients diagnosed with DLBCL between 2007 and 2014, registered in the Swedish Lymphoma register [21]. Among these, 685 patients were diagnosed at the Karolinska University Hospital in Stockholm. For the current study, a nested case–control design was used within the Karolinska Hospital cohort. The patients were followed for identification of refractory disease or relapse through Oct 31, 2017, with a median follow-up of 4.3 years (range 0 to 10.8 months) [22], and are henceforth referred to as the poor outcome group. Incidence density sampling was used to select controls from the underlying cohort of DLBCL patients who were alive and relapse-free at the time each case occurred, i.e. reflecting the same follow-up time (good outcome group). A total of 120 patients (50 cases and 70 controls) with sufficient paraffin embedded tumor tissue and optimal TLR9 immunohistochemical staining (IHC) were included in the final analysis. The workflow of identification of the study participants is presented in (Figure S1). A power calculation showed that with an exposure prevalence of 30% among the controls and a significance level of 5%, we had 80% power to detect an odds ratio (OR) of 3.00 with a sample size of at least 50 cases and 50 controls. Clinical data at diagnosis and during follow-up was available from the Swedish Lymphoma Register and previous medical records review [21, 22]. The clinical characteristics of interest included age, sex, Ann Arbor stage (I-IV), lactate dehydrogenase (LDH) level, performance status and nodal/extranodal presentation. Age, performance status, stage, LDH and > 1 extranodal location were summarized into the International Prognostic Index (IPI) score. Pathology reports were retrieved from the pathology database at the Department of Clinical Pathology and Cancer diagnostics, Karolinska University Hospital, Stockholm. DLBCL COO subtyping was performed according to the Hans algorithm [23].

The study was ethically approved (2015/2028 31/2).

### Immunohistochemical methods and detection of *MYD88 L265P* mutation

TLR9 protein expression was assessed by IHC using the diagnostic tissue biopsy taken prior to first-line treatment. In addition, full tissue sections from five reactive lymph nodes and two tonsils were included as controls. All IHC analyses were performed in the same research laboratory (Karolinska Institutet) using an identical protocol for all tumor samples, as previously described [24]. The IHC assessment was performed by two hematopathologists (SA and GR). Immunohistochemical scoring was based on counting at least 500 lymphoma cells from representative microscopy fields in each case, thus generating a percentage of TLR9-positive cells for each patient irrespective of staining intensity. A TLR9 expression of 20% was used to define high TLR9 expression (Figure S2). In a subset of samples with sufficient material, real-time PCR was conducted to detect the *MYD88* L265P mutation. Further details are provided in Supplementary methods.

### Inhibition of TLR9 activity

TLR9 inhibition was carried out on two GCB (MS and RCK8) and two ABC (OCI-LY3 and U2932) DLBCL cell lines by using the TLR9 antagonist ODN4084-F (Supplementary methods).

### Statistical methods

The relative risk of poor outcome by TLR9 expression and other factors was estimated with odds ratios (OR) and 95% confidence intervals (CI) using univariable and multivariable logistic regression. In the multivariable model, we adjusted for age, sex, COO, IPI, calendar period and nodal/extranodal presentation. Comparisons of TLR9 expression by COO groups and other clinicopathological features were performed with Mann–Whitney U test, Chi-square test or Fisher’s exact test. Comparisons of cell viability and growth percentage before and after treatment with TLR9 antagonist were performed using t-test.

All statistical analyses were performed using STATA and SAS software (College Station, Texas, USA) (Institute Inc., Cary, North Carolina, USA).

## Results

### *TLR9* gene expression in DLBCL

**Fig. 1:**
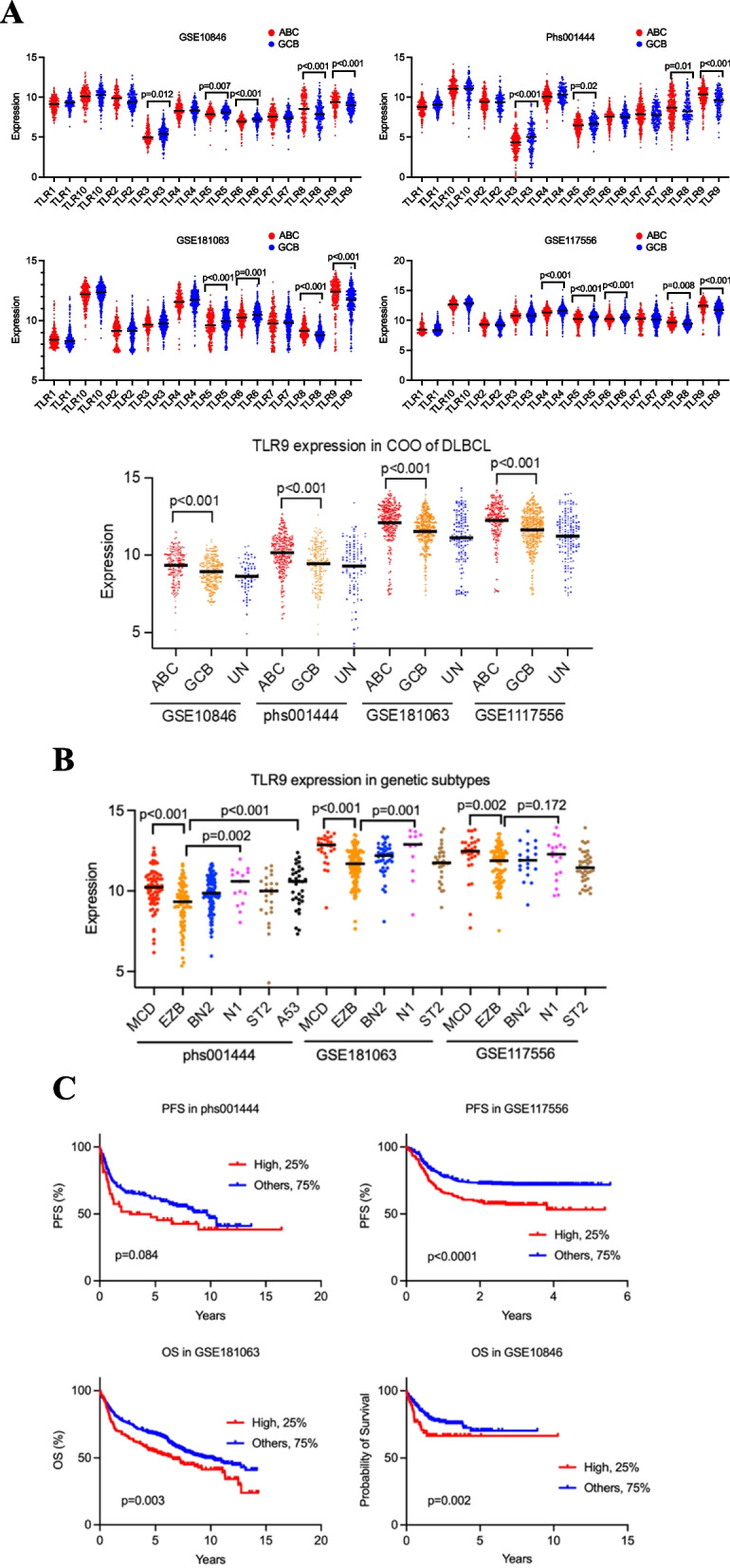
**A** TLR mRNA expression levels were assessed in GSE10846, phs001444, GSE117556, and GSE181063 cohorts by cell of origin. *TLR9* genes are highly expressed in ABC (*p* < 0.001) compared to GCB. **B** TLR9 mRNA expression levels were assessed in the four cohorts by genetic subtypes according to the Wright et al. classifier [3]. There was a higher *TLR9* expression in MCD, N1 and A53 subtypes compared to EZB subtype. **C** Kaplan–Meier survival analysis revealed that higher *TLR9* expression was associated with poorer overall survival (OS) and progression-free survival (PFS) across different cohorts. Patients were stratified as follows: within each cohort, the high-expression group comprised the top 25% of patients with the highest *TLR9* expression

Analysis of publicly available databases from four external DLBCL cohorts revealed that *TLR9* gene expression at the mRNA level was consistently higher in the ABC subtype compared to the GCB subtype across all cohorts (*p* < 0.001). Higher *TLR8* gene expression was also consistently observed in the ABC subtype, whereas higher *TLR5* expression was observed in the GCB subtype (Fig. 1 A). Due to the strong and consistent results for *TLR9* and its known dominant role in B cells, we focused on *TLR9* in further analyses. Genomic data on LymphGen subtype was available for three cohorts, where *TLR9* expression was highest in the MCD, N1 and A53 subtypes associated with poor outcomes (Fig. 1B) [4]. Patients with high *TLR9* gene expression (highest quartile) had worse PFS in two cohorts where this outcome could be assessed (significantly worse in one cohort and close to significant in the other, Fig. 1C). In the remaining two cohorts, high *TLR9* expression was associated with significantly worse OS compared to lower *TLR9* expression (Fig. 1 C).

### TLR9 protein expression in cell lines

**Fig. 2:**
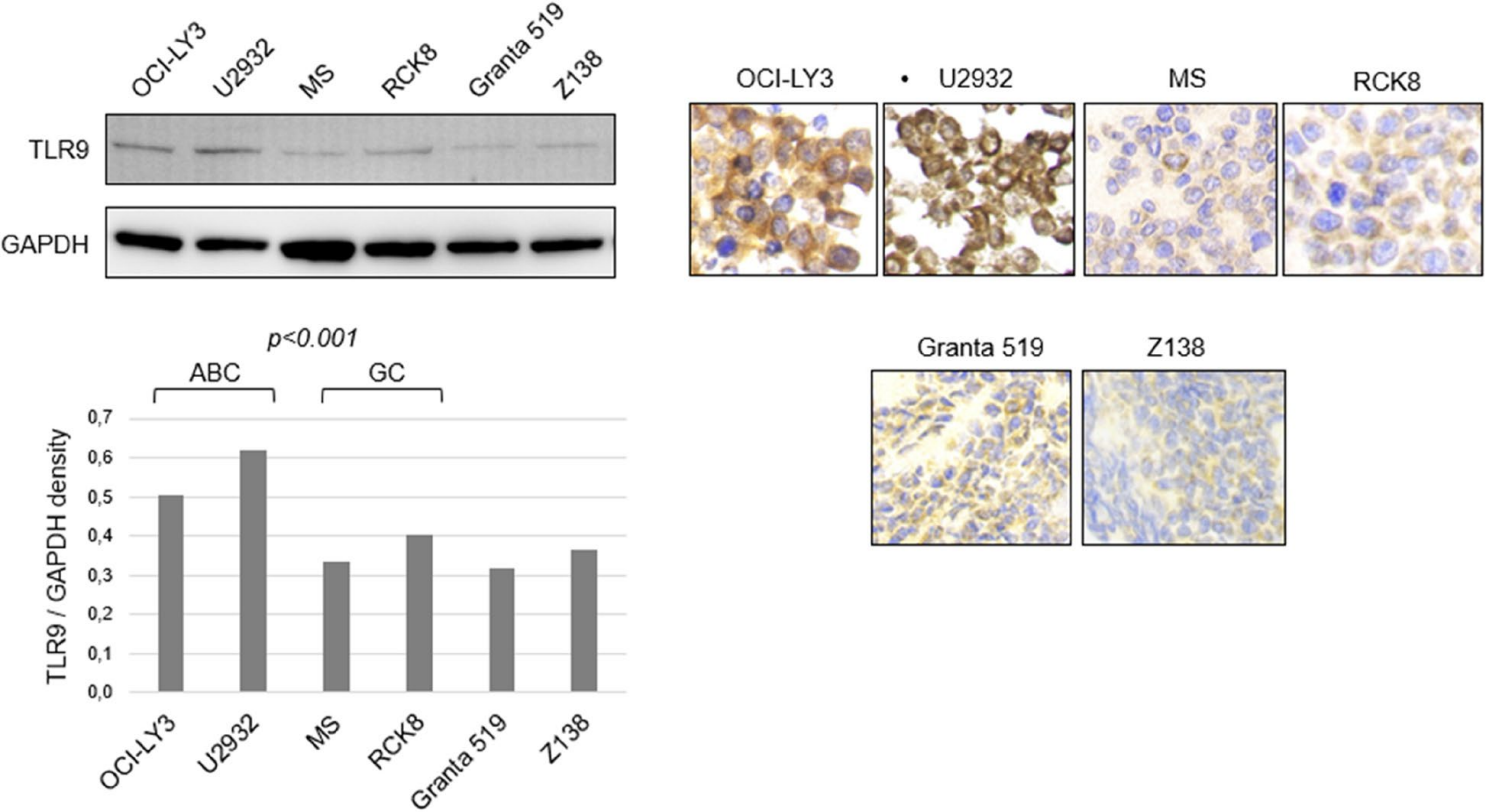
TLR9 protein expression on cell lines assessed by western blot (upper left panel) and measured by band density in (lower left panel). TLR9 immunohistochemical stains (right panel). Cell lines include Diffuse large B-cell lymphoma (DLBCL) activated B-cell (ABC) type (OCI-LY3 and U2932), germinal-centre B-cell (GCB) type (MS and RCK8), Mantle cell lymphoma (MCL) and (Granta519 and Z138). Higher expression of TLR9 in ABC compared to GCB

The baseline levels of TLR9 protein expression were assessed by Western blot analysis in ABC and GCB DLBCL cell lines. As shown in Fig. 2, the TLR9 protein levels were higher in the DLBCL cell lines of ABC type as compared to those of GCB type. The difference in the TLR9 protein expression level was confirmed by IHC performed on cell blocks prepared from the same DLBCL cell lines (Fig. 2). The *TLR9* mRNA expression was also significantly higher in the ABC cell line OCI-LY3 as compared to the GCB cell line MS (Figure S4).

### Characteristics of study population and TLR9 expression in primary DLBCL samples

**Fig. 3:**
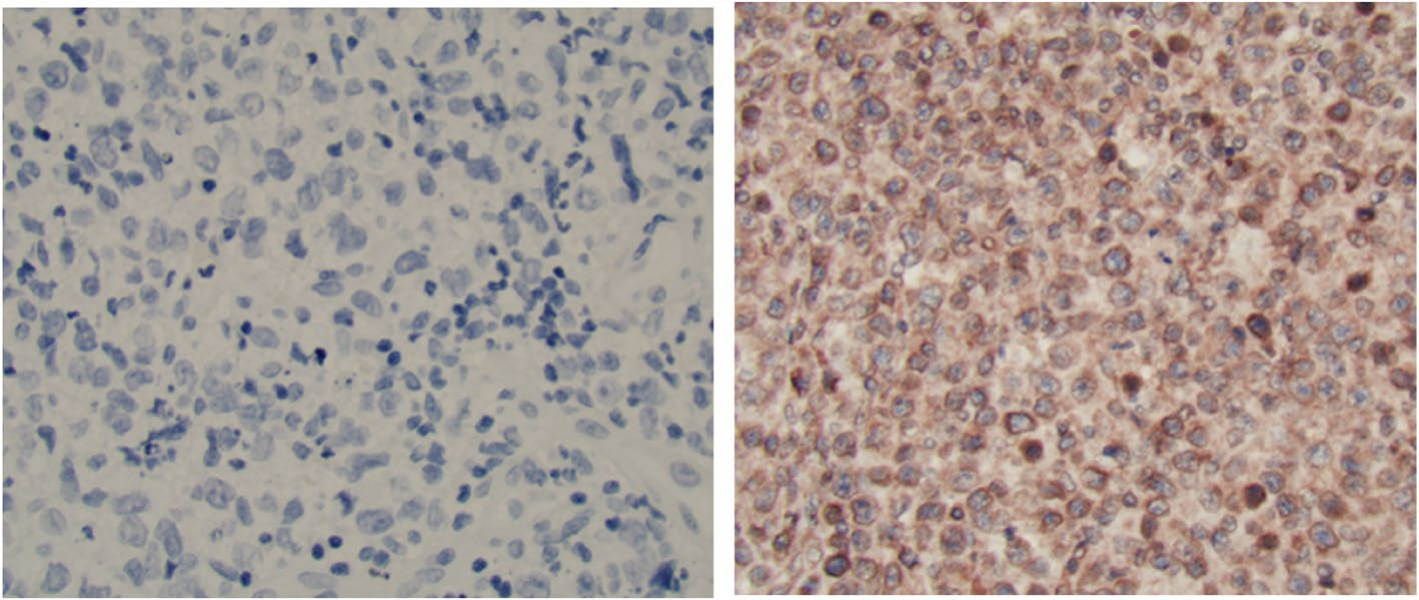
Immunohistochemical stain of TLR9 expression in DLBCL using monoclonal anti-TLR9 antibody distinguishing low (left) and high expression (right)

One hundred and twenty patients including 50 cases with poor outcome (refractory/relapsed DLBCL during follow-up) and 70 matched controls with good outcome (DLBCL in remission) were included in the final study population (Table 1). Using the predefined cut-off of 20%, TLR9 was high in 54 patients, of whom 31 (62%) had poor outcome while TLR9 was low in 66 patients, including only 19 (38%) with poor outcome (*p* < 0.001) (Table 1). Using IHC staining, TLR9 expression could be detected in the cell membrane and cytoplasm (Fig. 3). In the univariable analysis, TLR9 expression and IPI were associated with poor outcome, unlike other variables which did not differ significantly by case–control status (Table 1). In the multivariable analysis, adjusting for age, sex, COO, IPI, nodal/extranodal presentation, and calendar period of diagnosis, TLR expression remained associated with poor outcome (OR = 3.36, 95% CI 1.41–8.04, *p =* 0.01, Table 1).

**Table 1:** Clinicopathological features of the study population. Number and proportion of patients with Diffuse large B-cell lymphoma (DLBCL) in remission after primary treatment (good outcome) and with relapsed/refractory disease (poor outcome) during a median follow-up of 4.3 years, and relative risk (odds ratio, OR, with 95% confidence intervals, CI, and *p*-value) of poor outcome by clinicopathological features

Clinicopathological feature	Poor outcome *N = *50	Good outcome *N = *70	Relative risk of poor outcome DLBCL			
	**N (%)**	**N (%)**	**Univar OR (95% CI)**	***p*-value**	**Multivar**^**a**^** OR (95% CI)**	***p*-value**
Age				0.97		0.51
≤ 60	10 (20%)	15 (21%)	1.00 (reference)			
61–69	21 (42%)	28 (40%)	1.12 (0.42–2.99)		0.97 (0.32–2.98)	
≥ 70	19 (38%)	27 (39%)	1.06 (0.39–2.84)		1.71 (0.53–5.45)	
Sex				0.90		0.60
Male	27 (54%)	37 (53%)	1.00 (reference)			
Female	23(46%)	33 (47%)	0.95 (0.46–1.97)		0.80 (0.35–1.83)	
COO^b^				0.27		0.75
GCB	22 (44%)	38 (54%)	1.00 (reference)			
Non-GCB	28 (56%)	32 (46%)	1.51 (0.72–3.13)		1.14 (0.48–2.69)	
IPI				< 0.001		0.01
0–1	7 (14%)	34 (49%)	1.00 (reference)			
2–3	32 (64%)	31 (44%)	5.01 (1.93–12.9)		5.44 (1.98–14.9)	
4–5	11(22%)	5 (7%)	10.7 (2.81–40.5)		11.7 (2.77–49.3)	
Presentation				1.00		0.61
Nodal	30 (60%)	42 (60%)	1.00 (reference)			
Extranodal	20 (40%)	28 (40%)	1.00 (0.47–2.09)		0.91 (0.38–2.20)	
Diagnosis period				0.73		0.85
2007–2010	23 (46%)	30 (43%)	1.00 (reference)			
2011–2014	27 (54%)	40 (57%)	0.88 (0.42–0.82)		1.05 (0.46–2.38)	
TLR9 expression				< 0.001		0.01
Low	19 (38%)	47 (67%)	1.00 (reference)			
High	31 (62%)	23 (33%)	3.33 (1.56 −7.12)		3.36 (1.41–8.04)	

*Abbreviations*: *COO* cell of origin, *GCB* germinal center B-cell, *IPI* International Prognostic Index

^a^multivariable model adjusted for age, sex,COO, IPI, nodal-extranodal presentation, period of diagnosis

^b^According to the Hans algorithm

### *MYD88* L265P mutation

We could investigate *MYD88* L265P mutation status in 69 patients with available material. *MYD88* L265P mutation was present in 10% (3/30) of the patients with good outcome as compared to 18% (7/39) of patients with poor outcome (Suppl. Table 3). However, the difference was not statistically significant. In addition, no difference in the frequency of *MYD88* L265P mutation between TLR9-high and TLR9-low patients was observed; five mutated cases could be identified in each group (5/33 high and 5/36 low, p for difference 0.88). Also, there was no correlation between TLR9 expression by IHC and *MYD88* mutation status among patients with non-GC DLBCL (*n = *37) (data not shown).

### Cell growth and cell viability in response to TLR9 inhibition

The cell growth and viability of one cell line of ABC type (U2932) were evaluated after exposing the cells to the TLR9 inhibitor ODN4084-F. As the concentration of the TLR9 antagonist increased, cell growth (measured as number of viable cells/ml) gradually decreased, however, albeit to a lesser extent regarding viability (measured by trypan blue exclusion assay) (Figure S5). Using the TLR9 inhibitor at concentrations of 10 μΜ and 20 μΜ resulted in a significant, concentration-dependent decrease in cell growth in DLBCL cell lines of ABC type (U2932, OCI-LY3) but not in those of GCB type (MS, RCK8) (Figure S6). The effect of TLR9 inhibition on cell viability was small but slightly more prominent in the DLBCL cell lines of ABC type as compared to those of GCB type (Figure S7). These findings may suggest that TLR9 inhibition leads to cell cycle arrest rather than apoptosis in ABC-type DLBCL cells. In order to investigate the mechanism of cell cycle arrest upon TLR9 inhibition, Western blot analysis was performed. A concentration dependent decrease in the expression level of NF-kB transcription factor was found which was associated with upregulation of the universal cyclin-dependent kinase (CDK) inhibitor p27 and downregulation of phosphorylated NF-κB P65. The expression levels of certain anti-apoptotic proteins (i.e., BCL2) did not show any significant changes following TLR9 inhibition (Figure S8).

## Discussion

Our study based on data from publicly available DLBCL databases, DLBCL cell lines and patient samples confirms higher *TLR9* gene expression in ABC/non-GCB versus GCB DLBCL. High *TLR9* gene expression was further associated with poor outcomes (PFS or OS) in the external cohorts. TLR9 protein expression was evaluated on available archived tissue material in relation to risk of relapsed/refractory DLBCL using a nested case–control design. For the first time, we report that high TLR9 protein expression is associated with poor outcome DLBCL. TLR9 protein expression was associated with a statistically significantly increased risk of relapsed/refractory disease after adjustment for COO and other established clinical prognostic factors in multivariable analysis.

Although *TLR8* and *TLR5* shows a significant association with COO across all the four external cohorts, previous studies have shown that these TLRs are mainly expressed in myeloid cells such as monocytes, macrophages dendritic cells and neutrophils rather than B-cells [6, 25]. By further study of the potential impact of a TLR9 inhibitor on ABC and GCB cell lines, we present initial preliminary results of a possible effect on ABC DLBCL cell growth in vitro linked to NF-kB de-activation. These results warrant further exploration of the role of TLR9 in DLBCL progression and treatment.

To explore the biological impact of all ten TLRs, we analyzed four independent cohorts (phs001444, GSE117556, GSE181063 and GSE10846) with a total of 2474 DLBCL patients [17–20]. We observed significant associations between *TLR3-TLR10* expression and COO, though the patterns varied across cohorts. Notably, *TLR9* expression consistently showed higher levels in the ABC subtype across all four cohorts. Further analysis in three cohorts revealed that *TLR9* expression was highest in the MCD type (carrying both *MYD88* and *CD79B* mutations), N1 (*NOTCH1* mutation) and A53 (*TP53* mutations and/or deletion). These subtypes are associated with ABC subtype and with unfavorable survival [3, 17]. There is an indication that TLR9 is crucial for MCD by coordinating the protein supercomplex built of MYD88, TLR9, and IgM components of BCR (the My-T-BCR complex). This supercomplex initiates signaling cascades leading to NF-kB activation that regulates the expression of specific genes and eventually generate anti-apoptotic signals affecting survival of ABC DLBCL cells [9, 26]. However, no significant correlation was identified in our own cohort between *MYD88* L265P mutation and TLR9 expression by IHC or COO by the Hans classifier, perhaps because of limited sample size.

TLR9 protein was expressed in the cell membrane and in the cytoplasm. In ABC-DLBCL, chronic NF-κB activation may increase receptor internalization, leading to a dual localization pattern [27]. To our knowledge, only one previous study has investigated TLR9 expression by IHC in DLBCL. In this study, *TLR9* m RNA expression by RT-PCR on 41 patient samples were not significantly different by COO, and clinical parameters including age, sex or extranodal presentation. However, clinical outcome was not considered. High expression of TLR9 protein was observed in 34 DLBCL patient samples (75.6%), although correlations between TLR9 protein expression by IHC expression and clinicopathological features were not studied [28]

According to previous studies in other solid cancer forms, TLR9 protein expression has been associated with varying prognosis; in prostate cancer high TLR9 expression has been associated with poor prognosis [29], whereas in breast cancer treated with neoadjuvant chemotherapy, a favorable survival has been indicated [30]. In lymphoma, a recent study reported a positive correlation between high expression of TLR9 and PD-L1 with poor prognosis in angioimmunoblastic T-cell lymphoma (AITL) [31]. In our study, TLR9 IHC expression remained associated with poor outcome after adjustment for COO and clinical risk factors in multivariable analysis. In a large cohort of Chinese patients with DLBCL, genes involved in TLRs and tumor necrosis factor receptor (TNFR) pathways were more frequently mutated in patients who did not achieve complete remission [5]. However, that study did not address different types of TLR.

We further explored effects of TLR9 inhibition in cell lines of GCB and ABC type DLBCL. Here, we observed minimal effects on cell viability but significant effects on cell growth/number in ABC (not GCB) cell lines were suggested. Considering proteins engaging in the cell regulatory cycle, we observed an upregulation of cyclin-dependent kinase (CDK) inhibitors such as p27 with increasing concentrations of TLR9-inhibitor [32].

In general, TLRs can contribute to carcinogenesis by promoting tumor cell survival, proliferation, and generating of anti-apoptotic products induced by NF-kB or mitogen-activated protein kinase (MAPK) pathway activation. On the contrary, TLRs may also create anti-tumor responses by triggering the immune system in addition to its influence on the tumor microenvironment. The pro- and anti-tumor activity are determined by tumor and TLR type [33]. TLR9 acts as a checkpoint protein linking BCR and NF-kB leading to activation of the latter pathway via phosphorylation. Within this notion, evidence pointed towards the potential of TLR9 blockage in curbing DLBCL progression in a preclinical study model [15]. A phase 1/2 clinical trial has shown a potential therapeutic impact of TLR9 inhibition on ABC DLBCL bearing *MYD88* L265P mutation targeting TLR9 by an inhibitor referred as IMO-8400 in patients with relapsed or refractory DLBCL. This study tested the drug’s safety, tolerability, and efficacy, providing initial clinical evidence of IMO-8400’s therapeutic potential in DLBCL as the laboratory results showed decreases in tumor cells'survival and proliferation [15]. Furthermore, in a preclinical study, an antagonist of TLR9 and TLR7 labeled as HJ901 was investigated and was observed to inhibit tumor cell proliferation in animals and on three of five ABC but not GCB cell lines [14].

Several studies have also investigated the role of TLR9 in the tissue microenvironment of DLBCL. Studies demonstrated that DLBCL tumors infiltrated by neutrophils produce interleukin-8 (IL-8), which might trigger the production of neutrophil extracellular traps (NETs). In turn, this may lead to the activation of TLR9 and upregulation of NF-κB contributing to tumor progression and poorer survival [34, 35]. Since TLR9 plays a crucial role in the innate immune system through the recognition of microbial infection, high protein expression could reflect microenvironmental activation against microbial DNA in tumor cells. However, a more likely hypothesis concerns the direct stimulation of the NF-κB pathway which is considered as a hallmark of the ABC cell type promoting proliferation and cell survival.

The strengths of our study include the analyses across different materials and the careful study design and possibility to control for potential confounders in the analysis of patient samples. Nested case–control studies have a low risk of selection bias because cases and controls are drawn from the same well-defined cohort and follow up time. Another strength is that the IHC staining was performed in one center. On the other hand, since TLR9 staining is not part of the routine immunohistochemical analysis there is a future need to consider multicenter testing of this staining to achieve a marker with high accuracy. In the TLR9 IHC expression analysis, only lymphoma cells were considered. The background reflecting the microenvironment, including non-tumor cells such as reactive lymphocytes or dendritic cells, was not accounted for and used only as a control. Another limitation is the possible off-target effects of the TLR9 inhibitor tested in this study, which cannot be ruled out; these experiments aimed to provide an initial insight. Additionally, missing biomarker and mutation data such as *MYD88* mutations, which were assessed in only a limited number of cases restricted further analyses. Furthermore, the lack of independent validation remains an aspect that could be improved in the future. This spectrum of expression may need to be studied in more extensive materials including an independent cohort since we used heterogeneous types of tissue sections, including small biopsies, reflecting clinical practice.

## Conclusion

We demonstrate that *TLR9* mRNA and protein expression are elevated in the ABC/non-GC subtype of DLBCL and are associated with poor clinical outcomes. Further studies are required to validate these findings and to elucidate the mechanistic role of TLR9 signaling in DLBCL pathogenesis and treatment.

## Supplementary Information

Supplementary Material 1.

Supplementary Material 2.

Supplementary Material 3.

Supplementary Material 4.

## Data Availability

No datasets were generated or analysed during the current study.

## Abbreviations

TLR9: Toll-like receptor 9
DLBCL: Diffuse large B-cell lymphoma
COO: Cell-of-origin
GCB: Germinal centre B-cell
ABC: Activated B-cell
IPI: International prognostic index
NGS: Next generation sequencing
BTK: Bruton Tyrosine Kinase
IHC: Immunohistochemical
