# Supplementary material for: Toll-like receptor 9 (TLR9) expression correlates with cell of origin and predicts clinical outcome in diffuse large B-cell lymphoma
Source: BMC Cancer. 2025 May 28;25:959. doi: 10.1186/s12885-025-14359-7 (PMC12117956; doi:10.1186/s12885-025-14359-7)
Supplement: Supplementary file 1 — Supplementary Material 1. [file 12885_2025_14359_MOESM1_ESM.docx]

**SUPPLEMENTARY MATERIAL**

**Supplemental Tables**

**Suppl. Table 1: List of cell lines used in the study**

| **Cell line** | **Lymphoma type** | **Cell of origin** | **Source** |
| --- | --- | --- | --- |
| **OCI-LY3** | Diffuse large B-cell lymphoma | activated B-cell (ABC) | DSMZ, Germany |
| **U-2932*** | Diffuse large B-cell lymphoma | activated B-cell (ABC) |  |
| **MS**** | Diffuse large B-cell lymphoma | germinal center B-cell (GCB) |  |
| **RCK8** | Diffuse large B-cell lymphoma | germinal center B-cell (GCB) | DSMZ, Germany |
| **Granta519** | Mantle cell lymphoma |  | DSMZ, Germany |
| **Z138** | Blastoid mantle cell lymphoma |  | ATCC, USA |

* Kindly provided by by Dr. G. Enblad’s research group from (Uppsala University).

** Kindly provided by Dr. RJ. Ford, MD Anderson Cancer Center, Houston, TX, USA.

**Suppl. Table 2**: **Number and proportion of patients with Diffuse large B-cell lymphoma (DLBCL) with high and low TLR9 expression by clinicopathological features and outcome**.

| **Clinicopathological**  **feature** | | **TLR9**  **High**  **n=54 (45% of total)** | **TLR9**  **Low**  **n=66 (55% of total)** | **P value*** |
| --- | --- | --- | --- | --- |
| **Outcome** |  |  |  | 0.0016 |
| **Poor** |  | 31 (57) | 19 (29) |  |
| **Good** |  | 23 (43) | 47 (71) |  |
| **Age** |  |  |  |  |
| **>=70** |  | 21 (39) | 25 (38) | 0.12 |
| **61-69** |  | 26 (48) | 23 (35) |  |
| **<=60** |  | 7 (13) | 18 (27) |  |
| **Sex** |  |  |  | 0.77 |
| **Male** |  | 28 (52) | 36 (55) |  |
| **Female** |  | 26 (48) | 30 (45) |  |
| **Presentation** | |  |  | 0.33 |
| **Nodal** |  | 35 (65) | 37 (56) |  |
| **Extranodal** |  | 19 (35) | 29 (44) |  |
| **COO**** | |  |  |  |
| **GCB** |  | 19 (35) | 41 (62) | 0.003 |
| **Non-GCB** |  | 35 (65) | 25 (38) |  |
| **IPI** | |  |  |  |
| **0-1** | | 15 (28) | 26 (39) | 0.14 |
| **2-3** | | 30 (55) | 33 (50) |  |
| **4-5** | | 9 (17) | 7 (11) |  |

Abbreviations: COO= cell of origin, GCB= germinal centre B-cell IPI= International Prognostic Index

*P-values from chi2-test
** According to Hans algorithm

**Suppl. Table 3**: **Clinical and pathologic features of Diffuse large B-cell lymphoma (DLBCL) by *MYD88* L265P mutation or wild type (WT)**

| **Clinicopathological**  **feature** | | ***MYD88* L265P**  **N=10** | ***MYD88* WT**  **N=59** |
| --- | --- | --- | --- |
|  |  | **N (%)** | **N (%)** |
| **Outcome** |  |  |  |
| **Poor** |  | 7 (70%) | 32 (54%) |
| **Good** |  | 3 (30%) | 27 (48%) |
| **COO*** |  |  |  |
| **GCB** |  | 3 (30%) | 29 (49%) |
| **Non-GCB** |  | 7 (70%) | 30 (51%) |
| **Presentation** |  |  |  |
| **Nodal** | | 7 (70%) | 39 (66%) |
| **Extranodal** |  | 3 (30%) | 20 (34%) |
| **TLR9 IHC** |  |  |  |
| **low** |  | 5 (50%) | 31 (53%) |
| **high** |  | 5 (50%) | 28 (47%) |

Abbreviations: COO=cell of origin, IHC=immunohistochemistry
* According to Hans algorithm
