# Supplementary material for: Toll-like receptor 9 (TLR9) expression correlates with cell of origin and predicts clinical outcome in diffuse large B-cell lymphoma
Source: BMC Cancer. 2025 May 28;25:959. doi: 10.1186/s12885-025-14359-7 (PMC12117956; doi:10.1186/s12885-025-14359-7)
Supplement: Supplementary file 2 — Supplementary Material 2. [file 12885_2025_14359_MOESM2_ESM.docx]

**SUPPLEMENTARY MATERIAL**

**Supplementary text Methods section**

*Cell lines*

Cell line OCI-LY3 was obtained from the Leibniz-Institute DSMZ (Braunschweig, Germany). RCK8 was purchased from DZMS and authenticated through a PCR single-locus technology test conducted by Eurofins Genomics in Germany. U2932 provided kindly by Dr. G. Enblad’s research group from (Uppsala University). MS cell line Kindly provided by Dr. RJ. Ford, MD Anderson Cancer Centre, Houston, TX, USA, authenticated in his laboratory. Z138 purchases from the American Type Culture Collection (Manassas, USA) and Granta 519 from DSMZ. All cell lines were grown in Roswell Park Memorial Institute (RPMI)-1640 medium (Life Technologies, Grand Island, NY, USA) supplemented with 10% foetal bovine serum and incubated at 37°C in a humidified atmosphere containing 5% CO2. Western Blot analysis at baseline of TLR9 and the active subunit of NF-kB p65 (and GAPDH as control) was performed on all cell lines. Cell pellets were prepared using 20x106 cells and the pellets were fixed in 4% formalin for 24 hours and then embedded in paraffin to make cell blocks. Sections were cut from the cell blocks and the sections were subjected to IHC analysis.

*Detection of MYD88 L265P mutation in lymphoma samples*

DNA was isolated from 2×10 µm sections (approximately 1 cm² of tissue area) of formalin-fixed paraffin-embedded (FFPE) tissue curls using the xylene-free automated Maxwell® RSC FFPE DNA kit (Promega, Madison, USA). The extracted DNA was subsequently quantified using the NanoDrop® ND-2000 Spectrophotometer (ThermoFisher Scientific, Waltham, USA). To identify the MYD88 L265P mutation (Cosmic ID: COSM85940) within the genomic DNA, we utilized the commercially available IVD-qPCR kit PlentiPlex® (PentaBase, Odense, Denmark). Real-time PCR was conducted on the QuantStudio® 3 instrument (ThermoFisher Scientific, Waltham, USA) following the manufacturer's protocol.

*Protein expression*

In the Western Blot analysis at baseline, cells were collected during the exponential phase of growth and in experimental conditions at 48 hours following TLR9 inhibition and were washed twice in cold phosphate-buffered saline (PBS), and lysed at 4°C in lysis buffer. Western blot analysis was performed using standard methods, as reported previously ^1^ The primary antibodies used for this study were anti-TLR9 (cat. no. sc-52966, Santa Cruz Biotechnology, Dallas, TX, USA) at a dilution of 1:500, p NF-kB p65(S536) (cat. no. 3033, Cell Signalling Technology, Danvers, MA, USA) at a dilution of 1:1000, pNFkB p100 (S866/870) (cat. no. 4810, Cell Signalling Technology, Danvers, MA, USA) at a dilution of 1:1000, NF-kB p65 (cat. no. 8242, Cell Signalling Technology, Danvers, MA, USA) at a dilution of 1:1000, NFkB2 p100/p52 (cat. no. 4882, Cell signalling Technology, Danvers, MA, USA) and GAPDH (cat. no. ab8245, Abcam, Cambridge, UK) used at a dilution 1:5000.

*Gene expression analysis*

Baseline TLR9 mRNA expression was assessed by quantitative reverse transcription polymerase chain reaction (RT-qPCR) (Fig S4). RNA was isolated and purified using a PureLinkTM RNA Mini Kit (Invitrogen/Thermo Fisher Scientific, Waltham, MA, USA), following the manufacturer’s instructions. The RNA concentration in individual RNA samples was determined using an Invitrogen Qubit RNA BR Assay Kit. cDNA synthesis using a High-Capacity RNA-to-cDNATM Kit (Applied Biosystems/Thermo Fisher Scientific) and RT-qPCR were performed as described ^1^. Gene expression was normalized to the housekeeping GAPDH gene and the relative expression was calculated, converting the difference in cycle thresholds (∆Ct) using the 2−∆Ct method as described. Two mantle cell lymphoma cells lines, Granta 519 and Z138, served as positive control ^2^.

*Immunohistochemical methods*

A monoclonal rabbit anti-TLR9 antibody (cat. no. sc-52966, Santa Cruz Biotechnology, Dallas, TX, USA) and the UltraVision LP Detection System, Large Volume HRP Polymer (RTU) (Thermo Fisher Scientific, Stockholm, Sweden) were utilized. The specificity of the TLR9 monoclonal antibody was tested by Western blot analysis in lymphoma cell lines and IHC staining on the cell blocks prepared from the same cell lines. The staining patterns were evaluated over the range of the entire samples to establish a cut-off to define a dichotomized expression. Based on the distribution of data (percentage of positive tumour cells) among DLBCL, a 20% cut-off for TLR9 expression was chosen (Fig S2), which was also used by others ^3^ Non-tumour small, reactive lymphocytes, dendritic cells or macrophages were used as internal positive and negative controls, while reactive lymph nodes and tonsils were used as external controls (Fig S3) Membranous and cytoplasmic staining of the tumour cells was considered positive irrespective of intensity.

*Inhibition of TLR9 activity*

The cell lines (1x106 cells in 1 ml culture medium) were treated with a serial of concentrations, 1 μΜ, 5 μΜ, 10 μΜ and 20 μΜof TLR9 antagonist ODN4084-F (cat. no. tlrl-4084, Invivo Gen, Toulouse, France) or an equivalent amount of Limulus Amebocyte Lysate (LAL) reagent water as control for 48 hours. Cell viability was assessed using trypan blue exclusion assay in triplicate, and the mean percentage of viable cells was calculated. More specifically, the number of viable (and dead) cells was calculated in three different samples for each condition after trypan blue staining. Cell viability was defined as the mean percentage of viable cells and cell number/ml medium as the mean number of viable cells per ml of medium (cell concentration), which is indicative of the cell growth. The differences in cell number/ml medium and cell viability across various experimental conditions were statistically analysed with t-test as shown.’

*References:*

1. Xagoraris I, Farrajota Neves da Silva P, Kokaraki G, et al. Sting Is Commonly and Differentially Expressed in T-and Nk-Cell but Not B-Cell Non-Hodgkin Lymphomas. Cancers. 2022;14: 1186.

2. Uhlén M, Fagerberg L, Hallström BM, et al. Proteomics. Tissue-based map of the human proteome. Science. 2015;347: 1260419.

3. Huang W-T, Weng S-W, Huang C-C, Lin H-C, Tsai P-C, Chuang J-H. Expression of Toll-like receptor9 in diffuse large B-cell lymphoma: further exploring CpG oligodeoxynucleotide in NFκB pathway. APMIS. 2012;120: 872-881.
