# Supplementary material for: Toll-like receptor 9 (TLR9) expression correlates with cell of origin and predicts clinical outcome in diffuse large B-cell lymphoma
Source: BMC Cancer. 2025 May 28;25:959. doi: 10.1186/s12885-025-14359-7 (PMC12117956; doi:10.1186/s12885-025-14359-7)
Supplement: Supplementary file 3 — Supplementary Material 3. [file 12885_2025_14359_MOESM3_ESM.docx]

**SUPPLEMENTARY MATERIAL**

**Supplemental Figures**

**
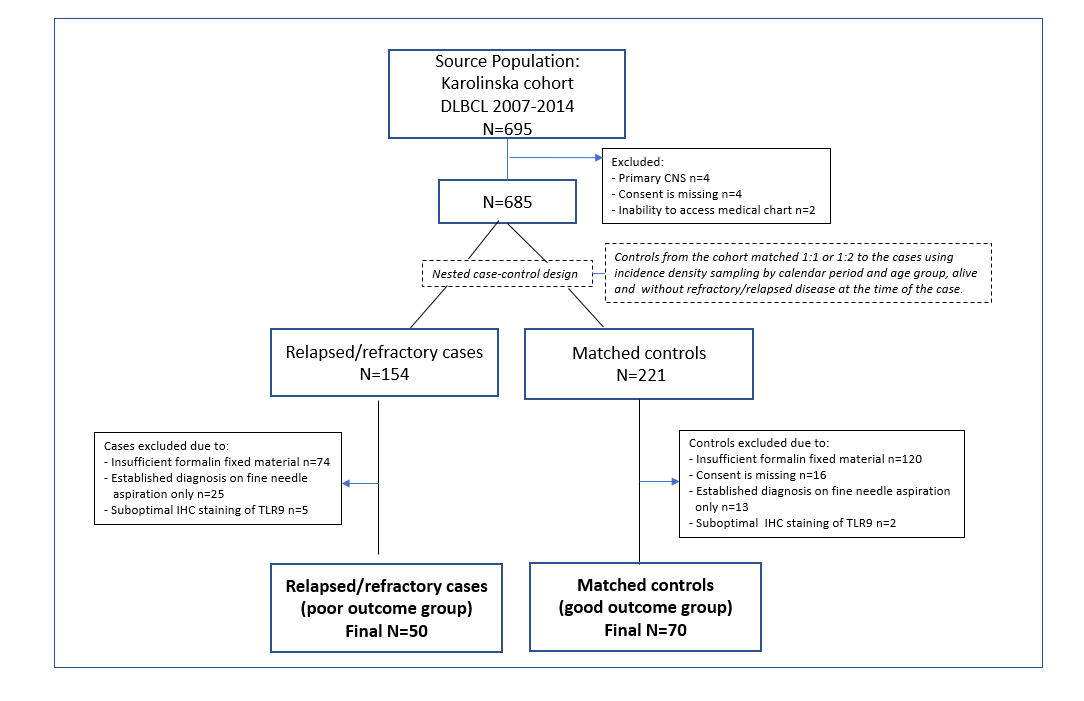
Figure S1**

**Figure S1:** The flow work diagram of the study population.

**Figure S2**


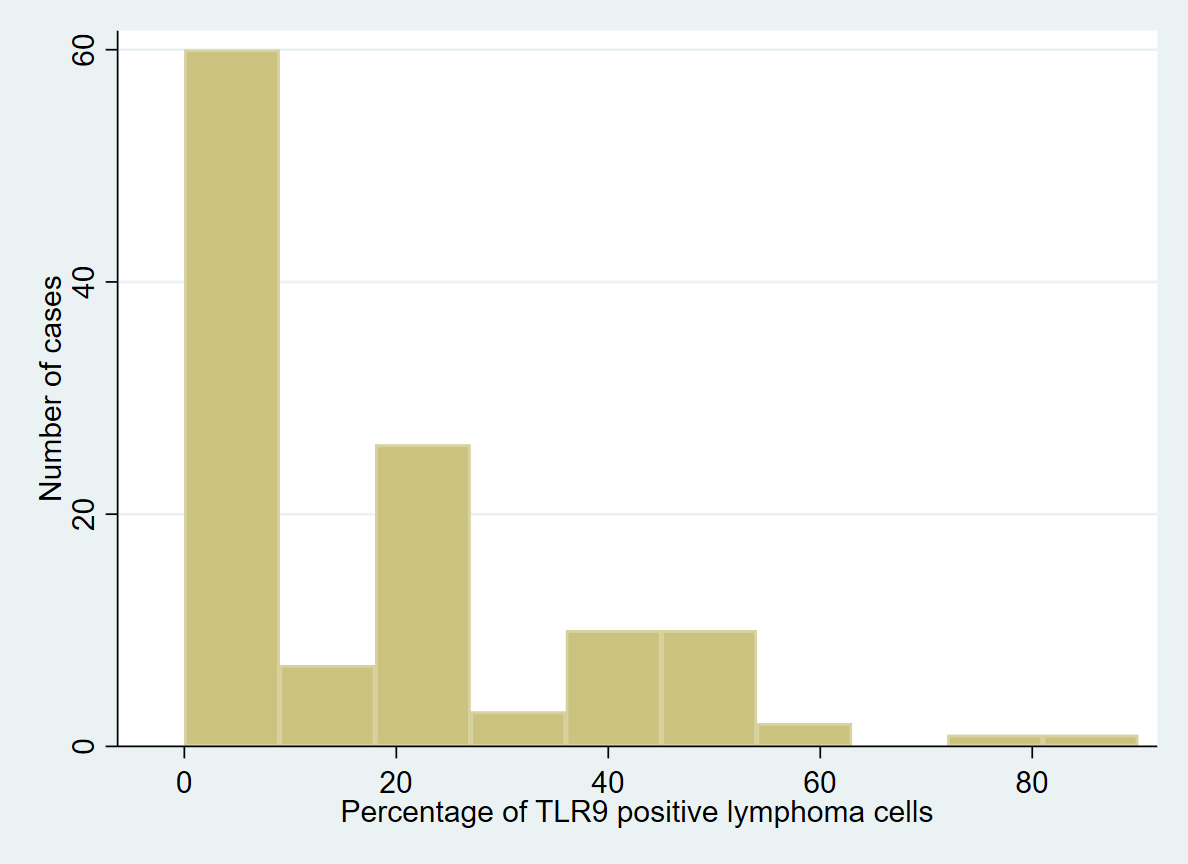


**Figure S2:** Distribution of percentage of total TLR9 positive lymphoma cells versus the number of tissue samples.

**Figure S3**

**
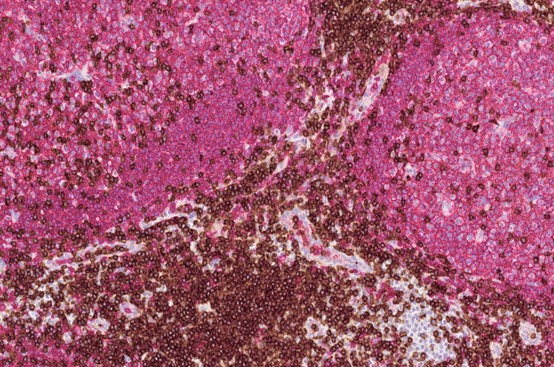

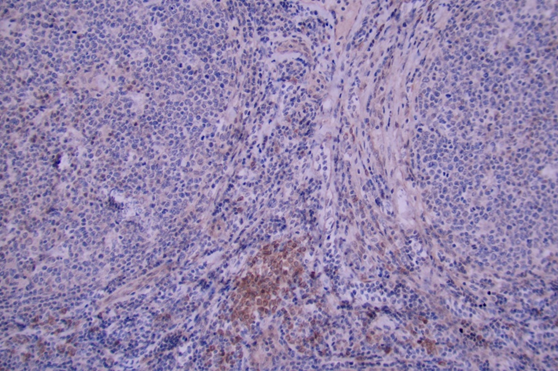
**
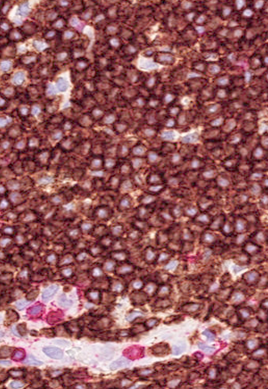

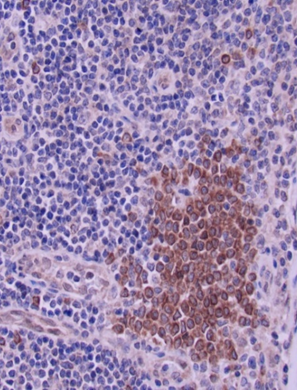
**A**

A: Left upper and lower; High TLR9 expression in a subset of T cells in the interfollicular area of reactive lymph node. Right upper; double stains CD3 brown and CD20 red. Right lower; CD4 brown and CD8 red showing corresponding T-cells with high TLR9 expression are CD4 positive.


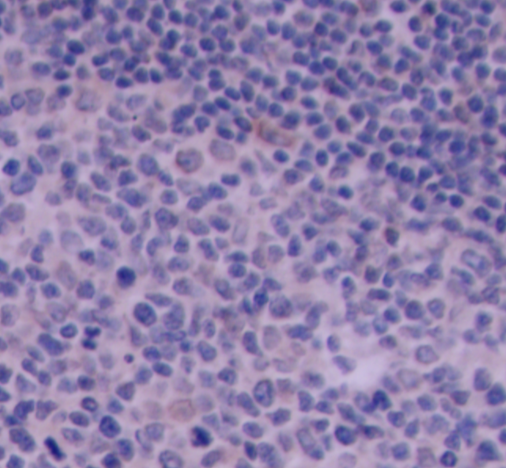
**B**

**
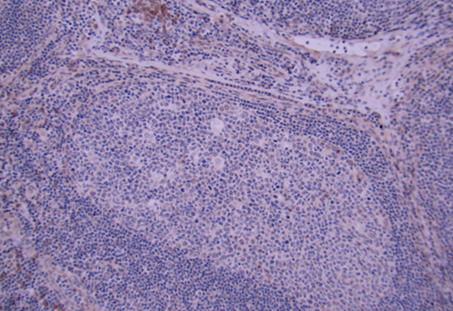
**

B: moderately high expression of TLR9 in dendritic cells.

**Figure S4**

**Figure S4**: Significant high *TLR9* mRNA level in ABC cell line OCI-LY3 compared to GCB cell line MS. Control positive TLR9 mRNA represented by mantle cell lymphoma cell lines Granta 519 and Z138. The experiment was repeated at least twice with similar results.

**Figure S5**


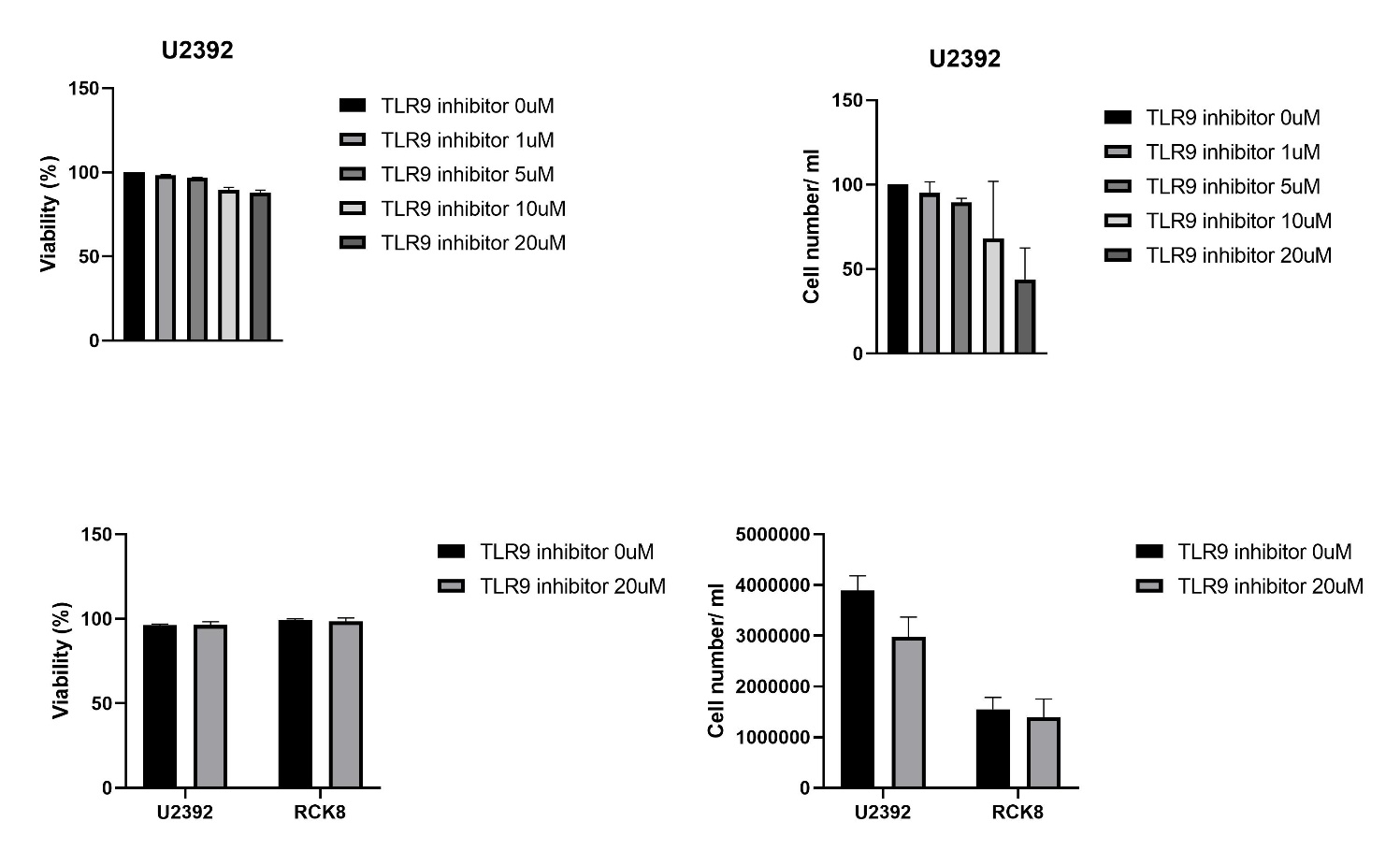


**Figure S5**: Viability and cell growth post ODN4084-F (TLR9 inhibition) in U2932 cells.

The standard error bars represent three different counts of cell viability and cell number / mL. The experiment was repeated at least twice with similar results.

**Figure S6**

**
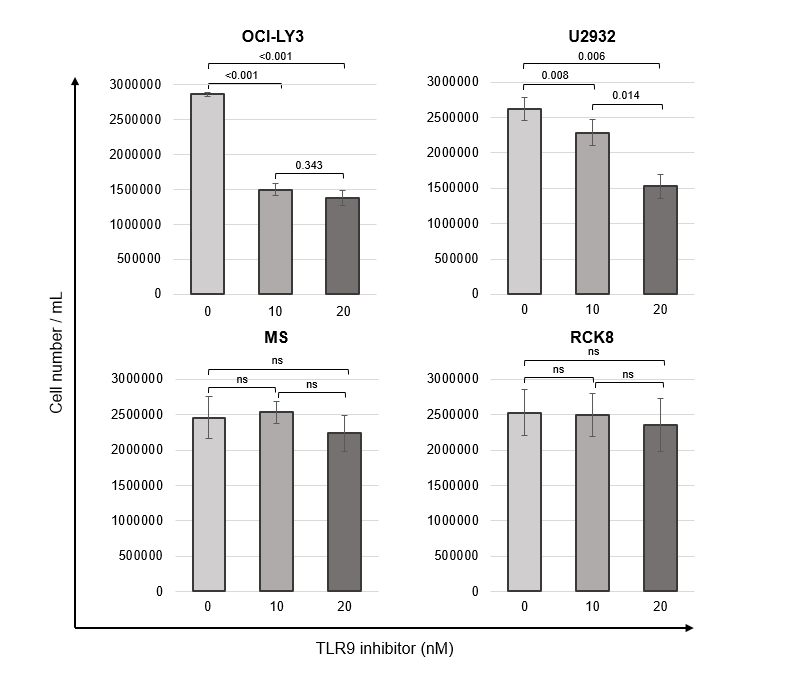
**

**Figure S6:** Cell growth (number of viable cells/ml) after treatment of Diffuse Large B-cell lymphoma (DLBCL) cell lines; ABC (OCI-LY3 and U2932) and GCB (MS and RCK8) with the TLR9 inhibitor ODN4084-F. Significant decrease in cell growth was observed in DLBCL lines of ABC type but not in those of GCB type (p<0.05, significant; ns=not significant; paired t-test). The standard error bars represent three different counts of cell viability and cell number / ml. The experiment was repeated at least twice with similar results.

**Figure S7**

**
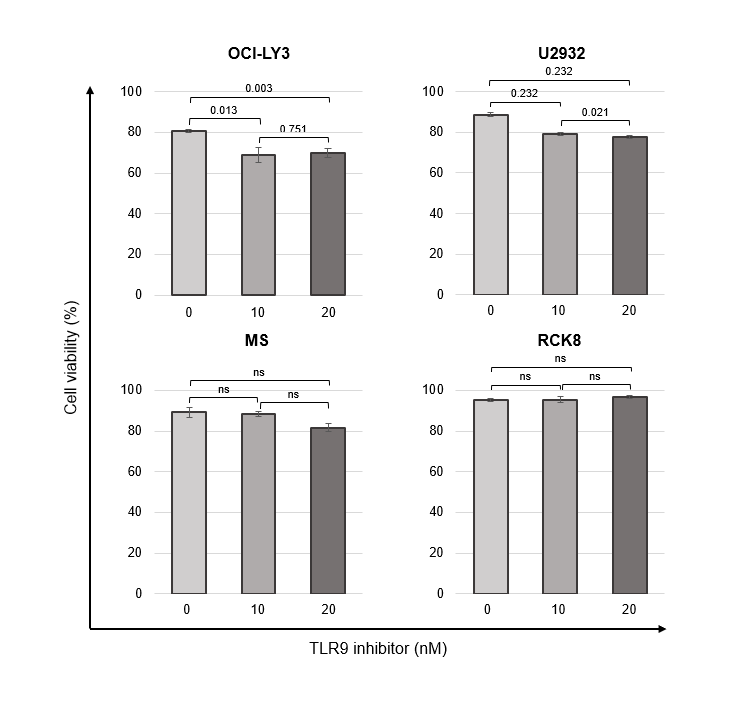
**

**Figure S7:** Cell viability after treatment of Diffuse Large B-cell lymphoma (DLBCL) cell lines (A: ABC; B: GCB) with the TLR9 inhibitor *ODN4084-F*. Minimal decrease in cell viability was observed in DLBCL lines of ABC type but not in those of GCB type (p<0.05, significant; ns=not significant; paired t-test) ns=not significant; paired t-test). The standard error bars represent three different counts of cell viability and cell number/mL. The experiment was repeated at least twice with similar results.

**Figure S8**

**
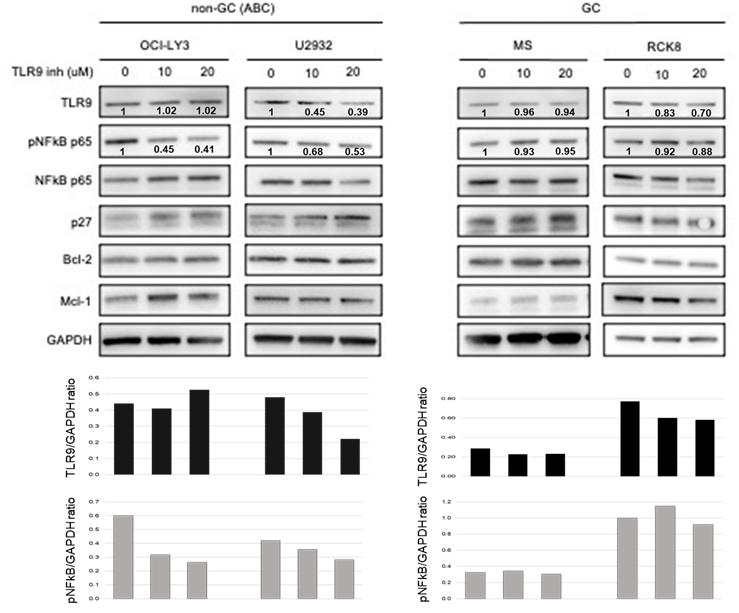
**

**Figure S8:** Western blot analysis by using TLR9 inhibitor ODN4084-F on ABC and GCB cell lines showing the effect on expression of proteins involved in the cell cycle and cell survival by increasing TLR9 inhibitor concentration. The effects of the TLR9 inhibitor on cell growth were associated with a concentration-dependent increase of the universal CDK inhibitor p27 and a decrease of the NF-κB activation. The numbers underneath the band for TLR9 and phospho NF-κB p65 represent the relative density ratio normalized with GAPDH levels.
