# Supplementary material for: Toll-like receptor 9 (TLR9) expression correlates with cell of origin and predicts clinical outcome in diffuse large B-cell lymphoma
Source: BMC Cancer. 2025 May 28;25:959. doi: 10.1186/s12885-025-14359-7 (PMC12117956; doi:10.1186/s12885-025-14359-7)

Figure 2

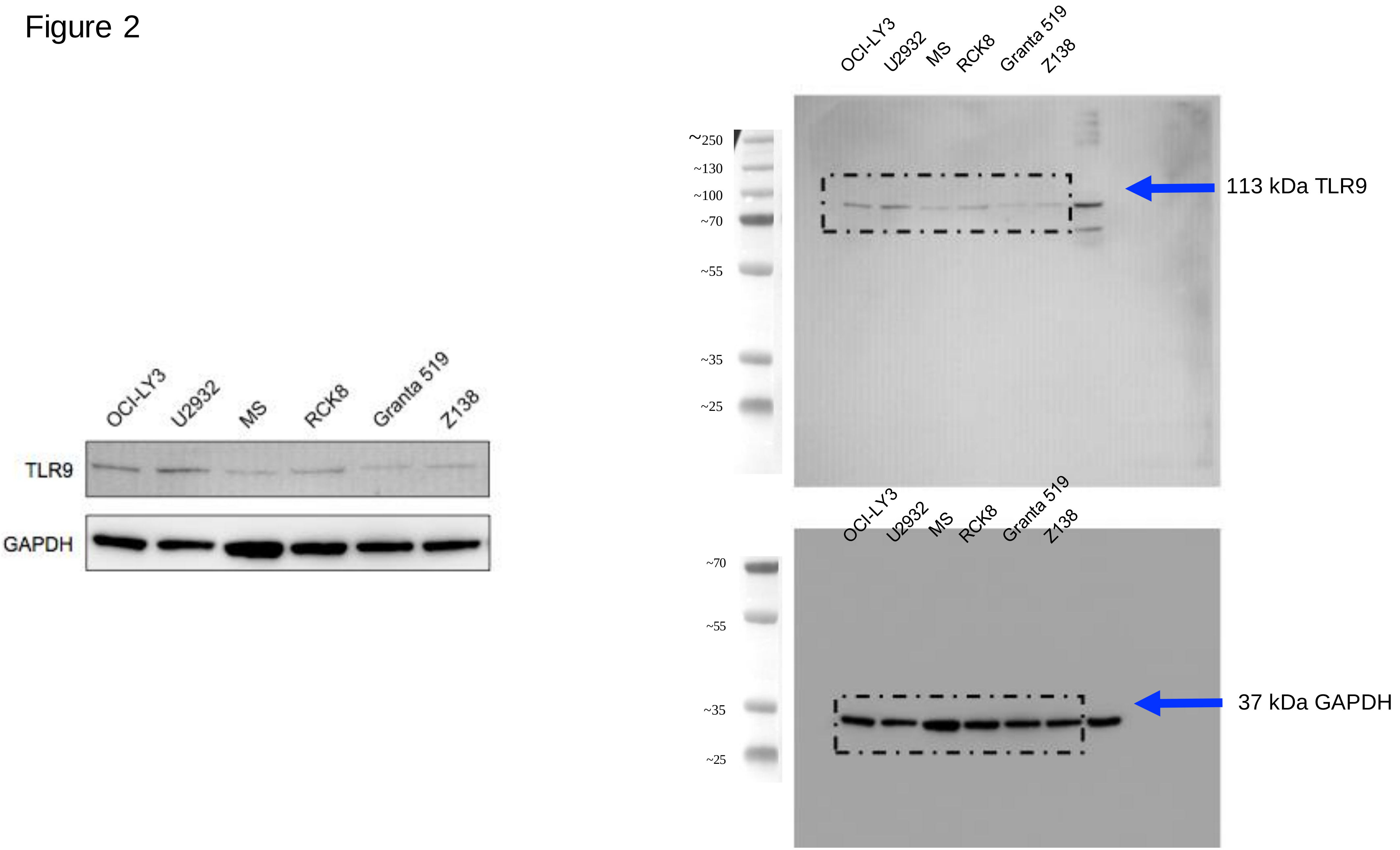
OCI-LY3
U2932
MS
RCK8
Granta 519
Z138
TLR9
GAPDH
~250
~130
~100
~70
~55
~35
~25
113 kDa TLR9
OCI-LY3
U2932
MS
RCK8
Granta 519
Z138
~70
~55
~35
~25
37 kDa GAPDH

Figure S8

TLR9

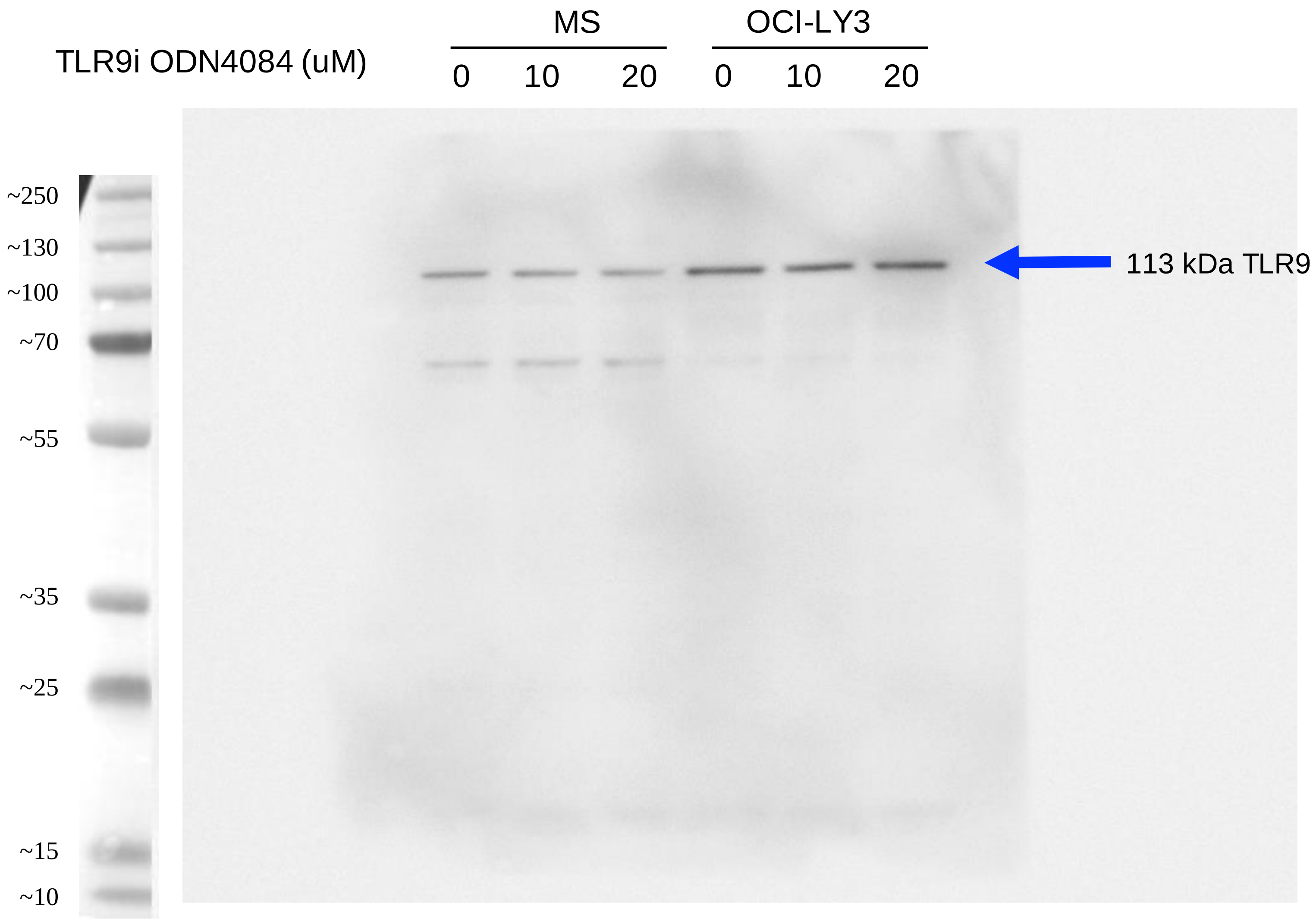

Figure S8

TLR9

The crossed immunoblots refer to another project and Is not related to this paper

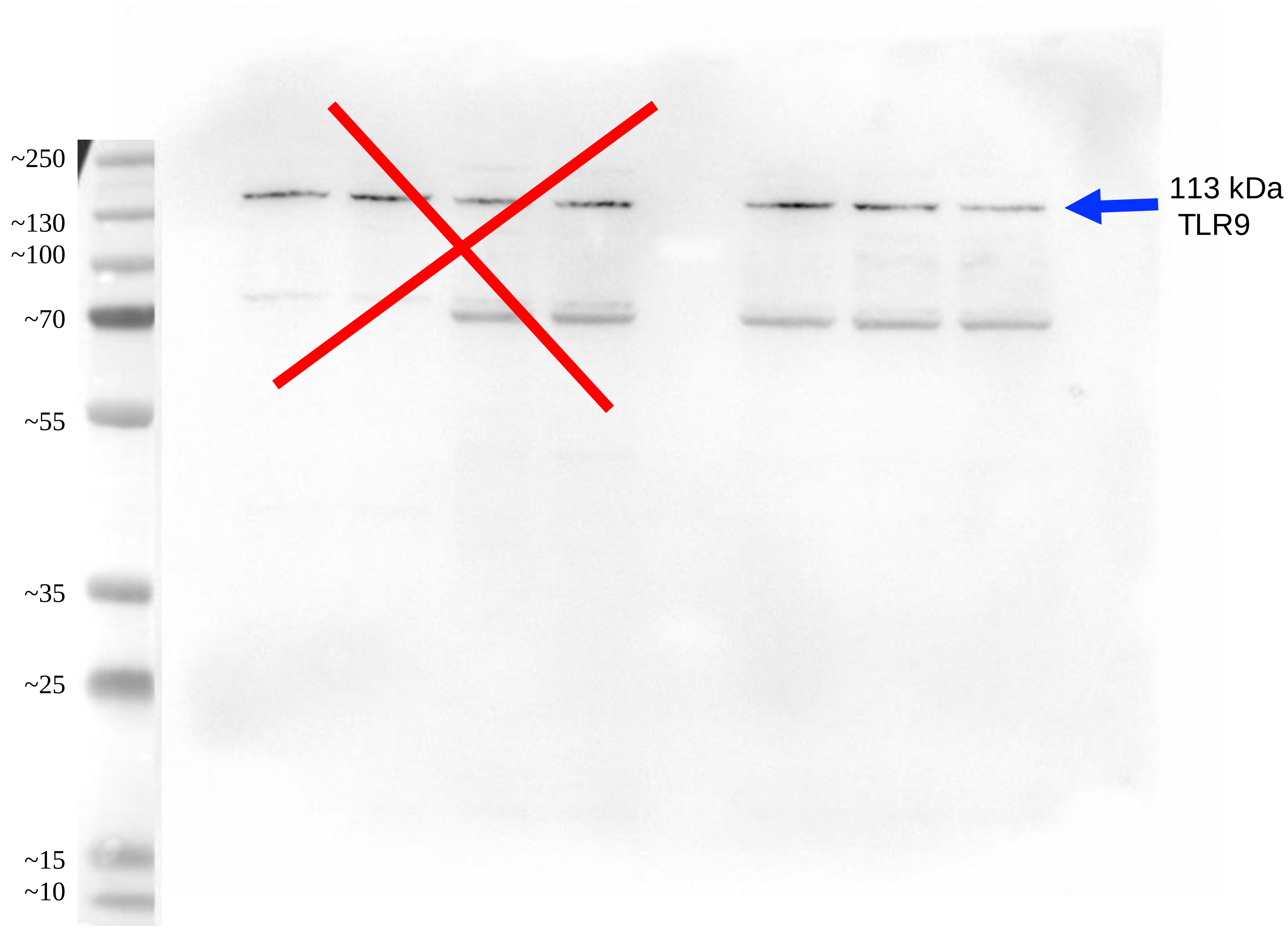

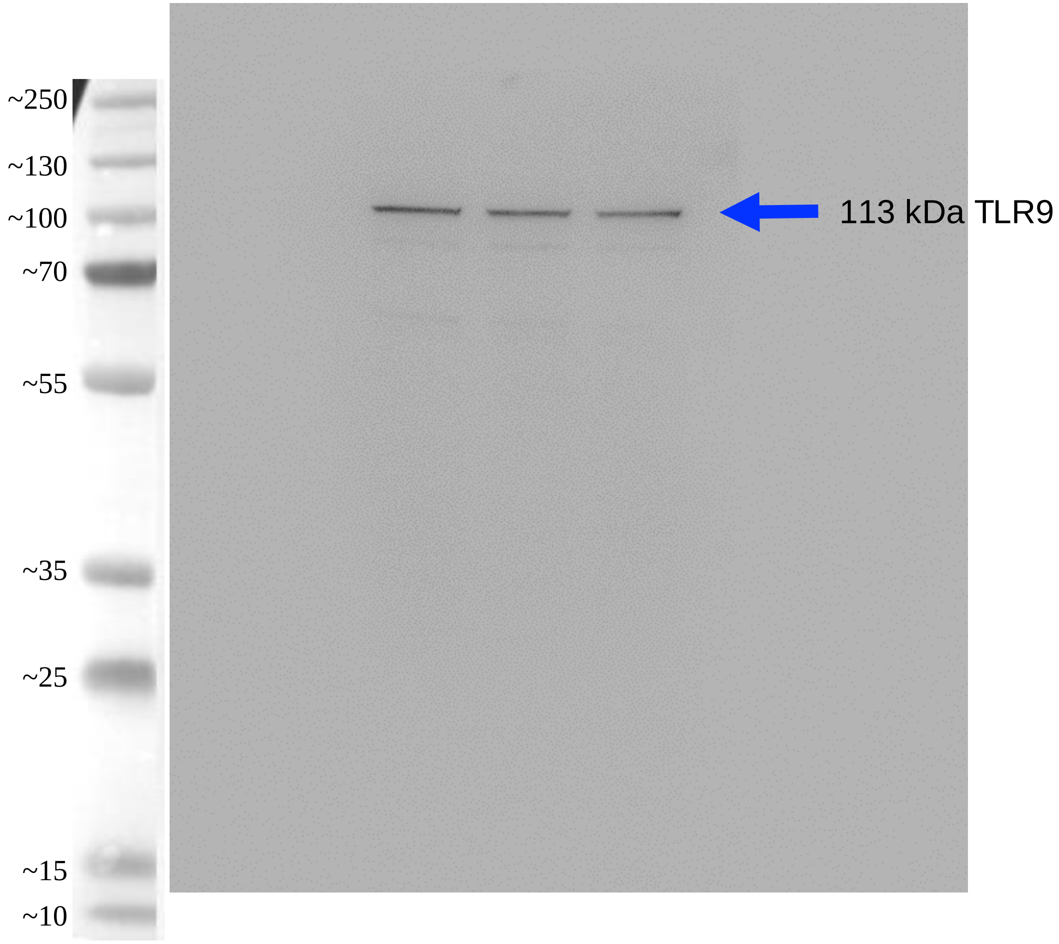

Figure S8

pNFκB p65

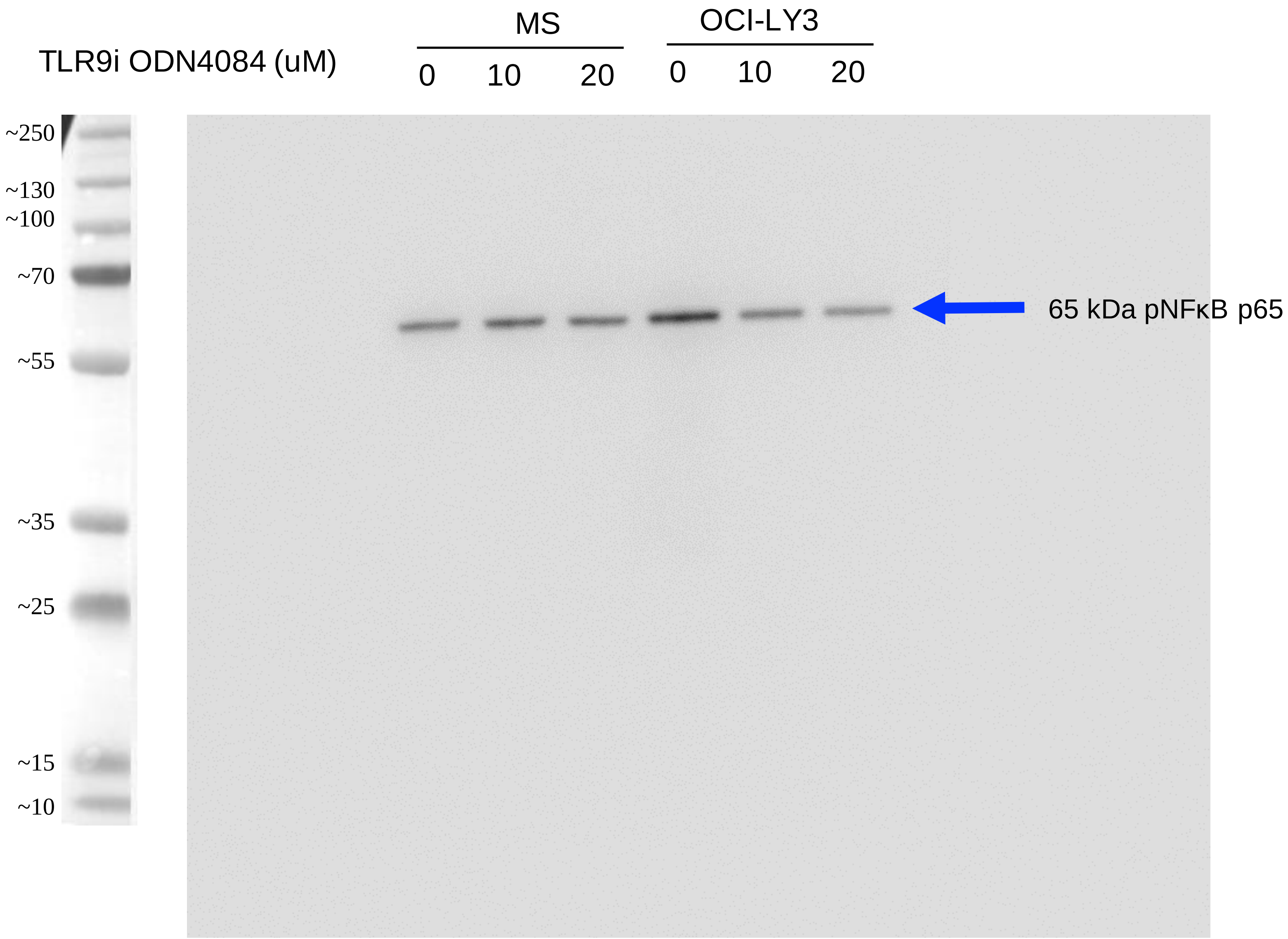

Figure S8

pNFκB p65 (longer exp.)

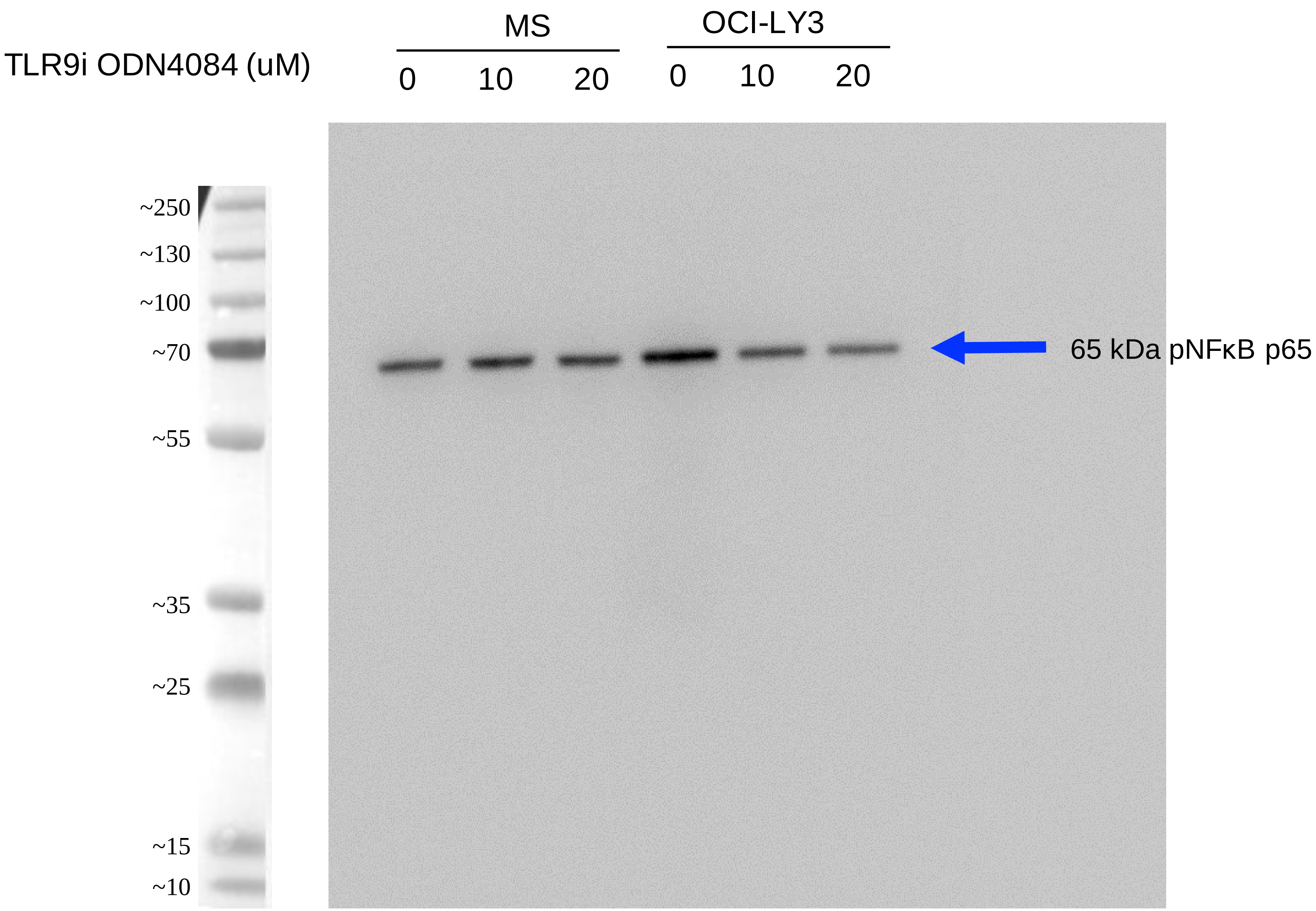

Figure S8

pNFκB p65

The crossed immunoblots refer to another project and Is not related to this paper

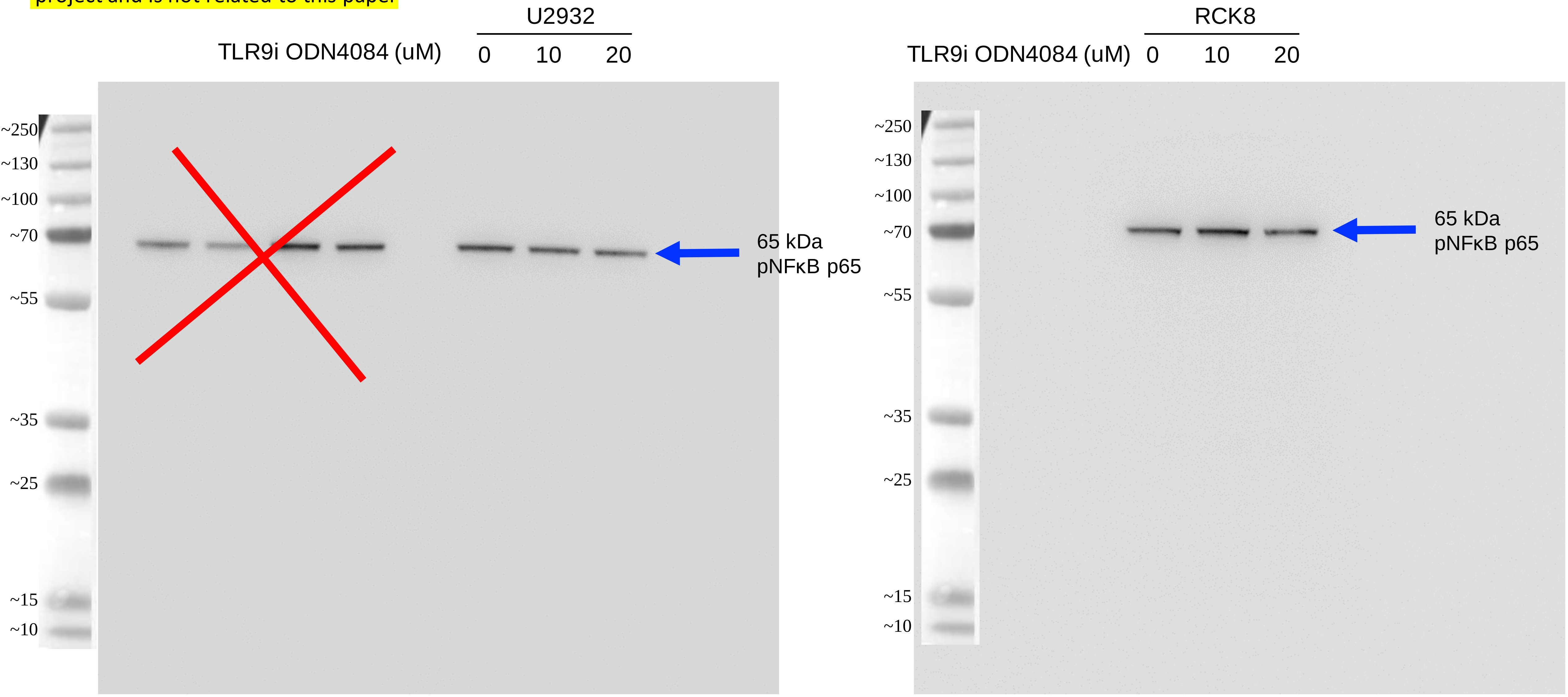

Figure S8

pNFκB p65 (longer exp.)

The crossed immunoblots refer to another project and Is not related to this paper

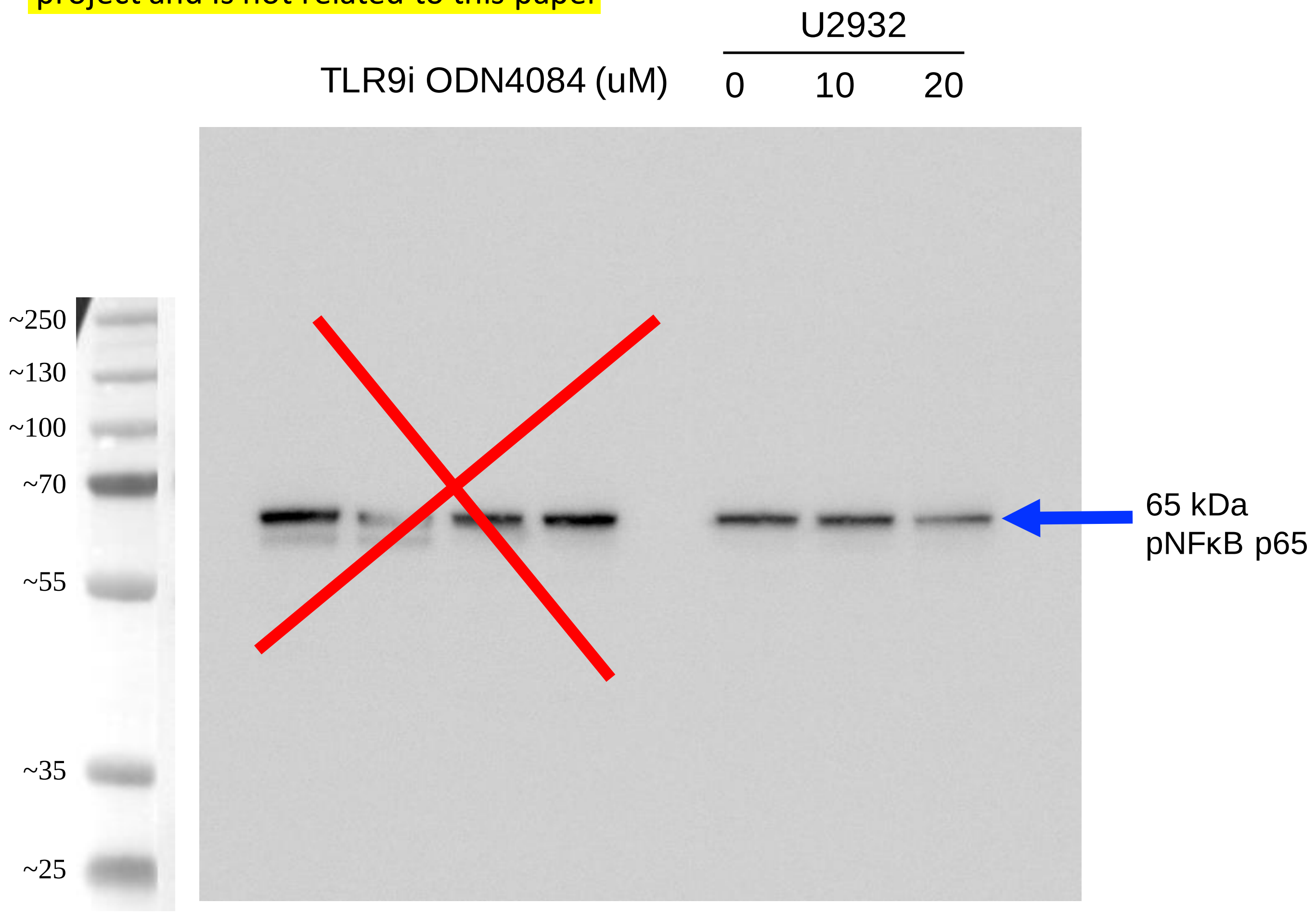

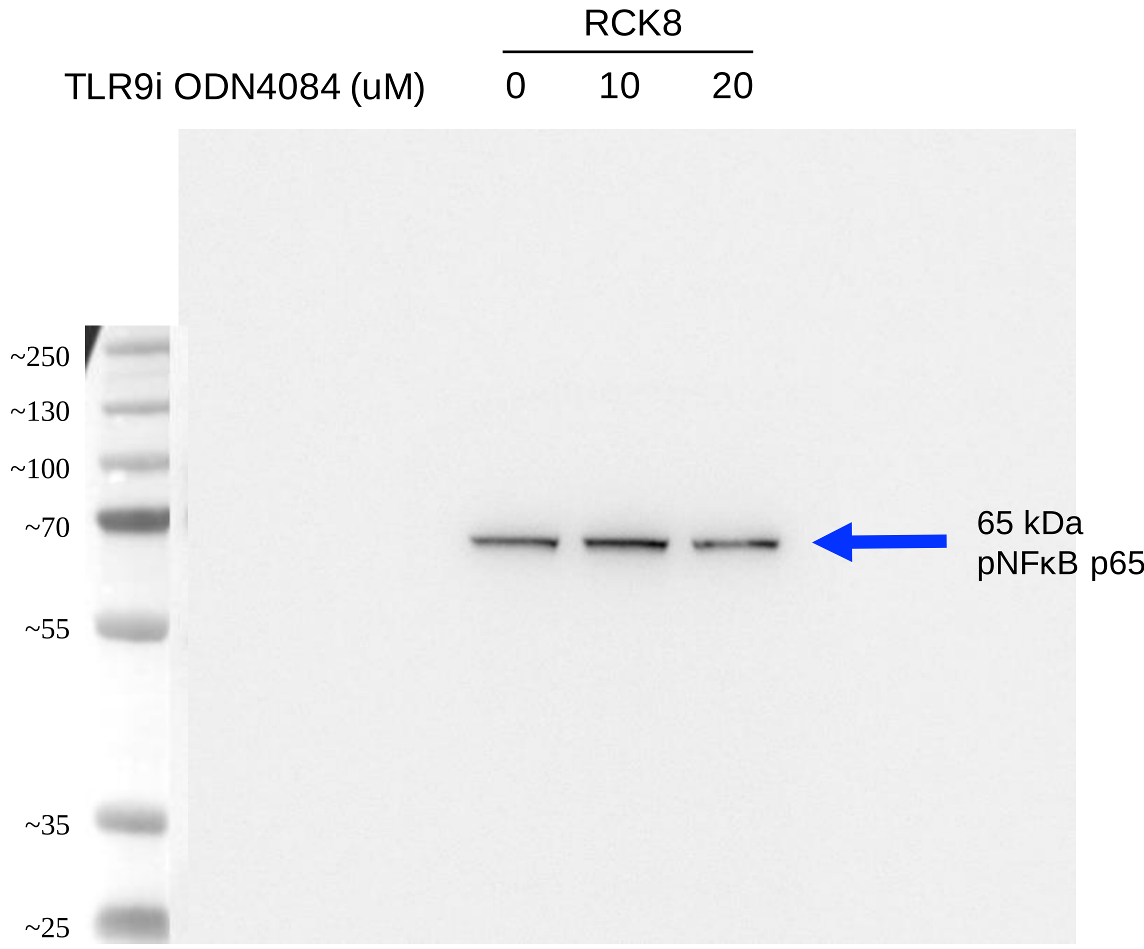

Figure S8

NFκB p65

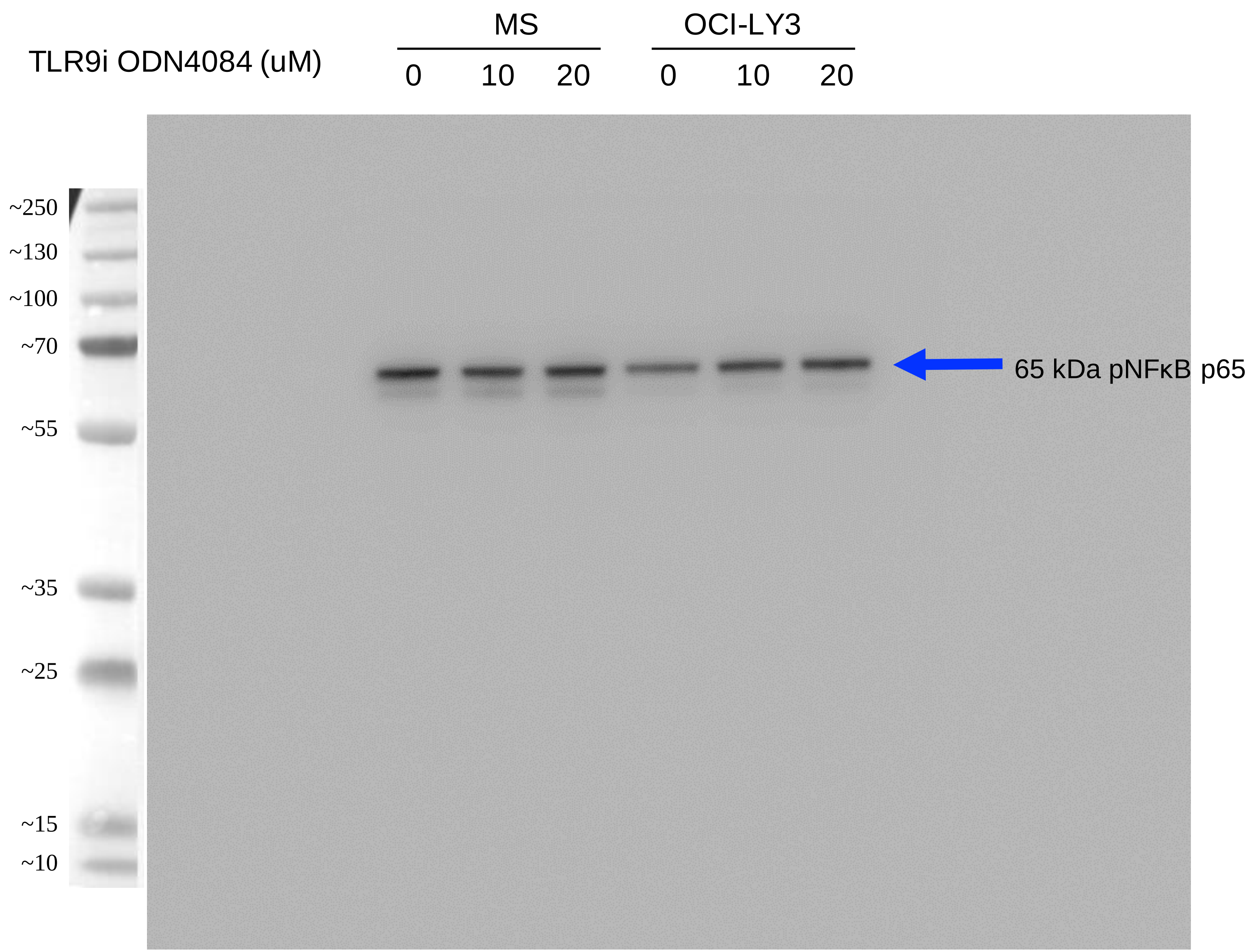

Figure S8

NFκB p65

The crossed immunoblots refer to another project and Is not related to this paper

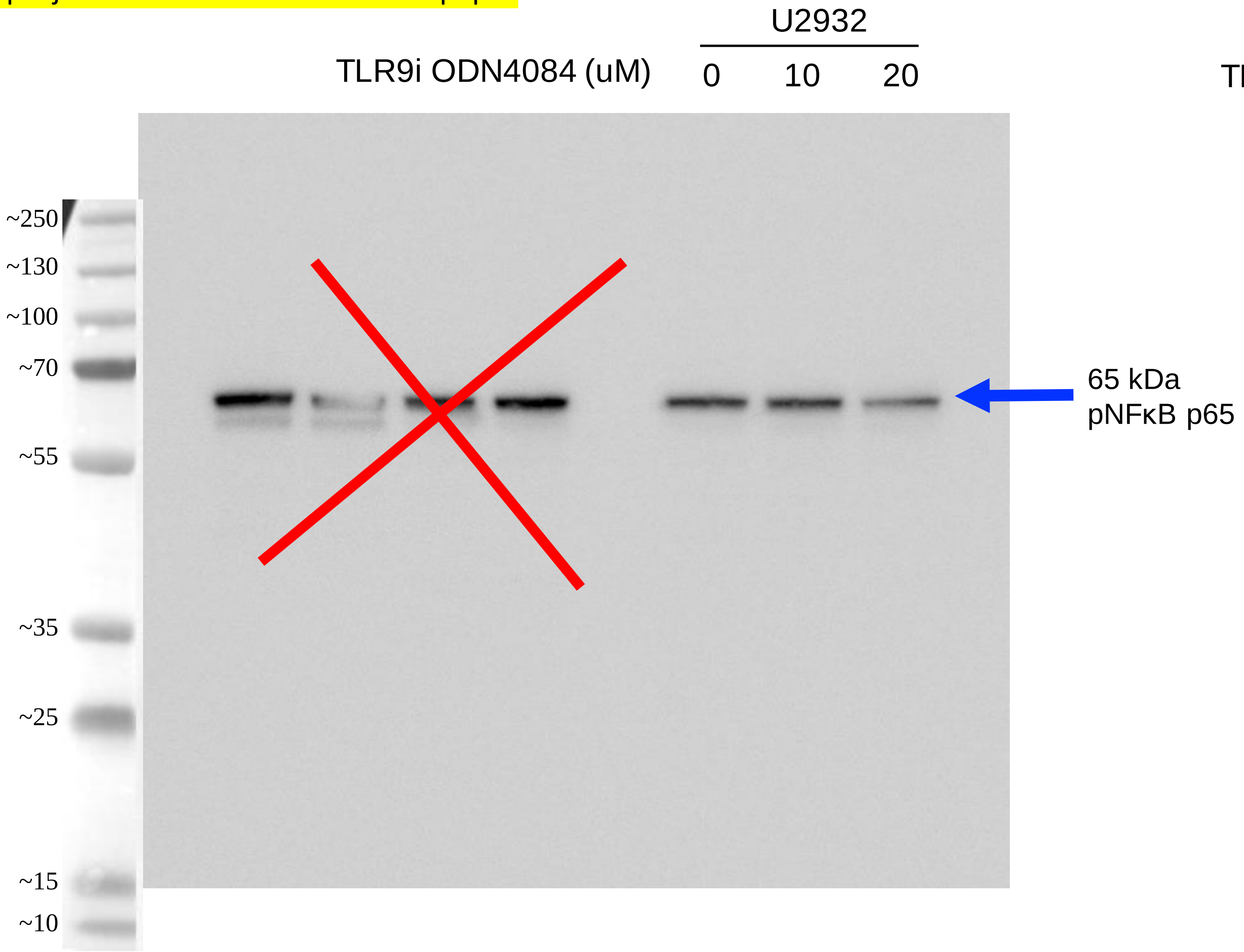

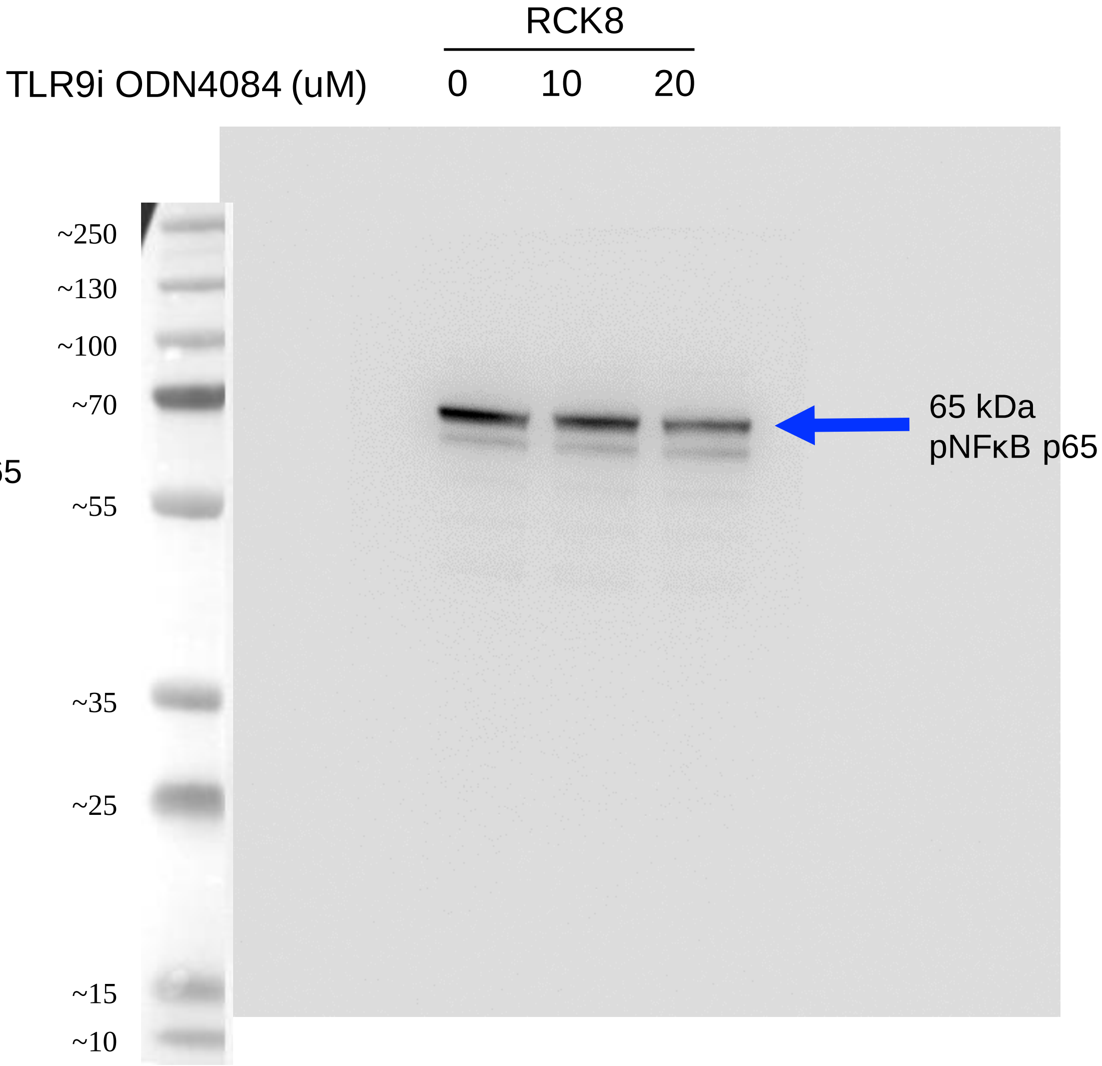

Figure S8

p27

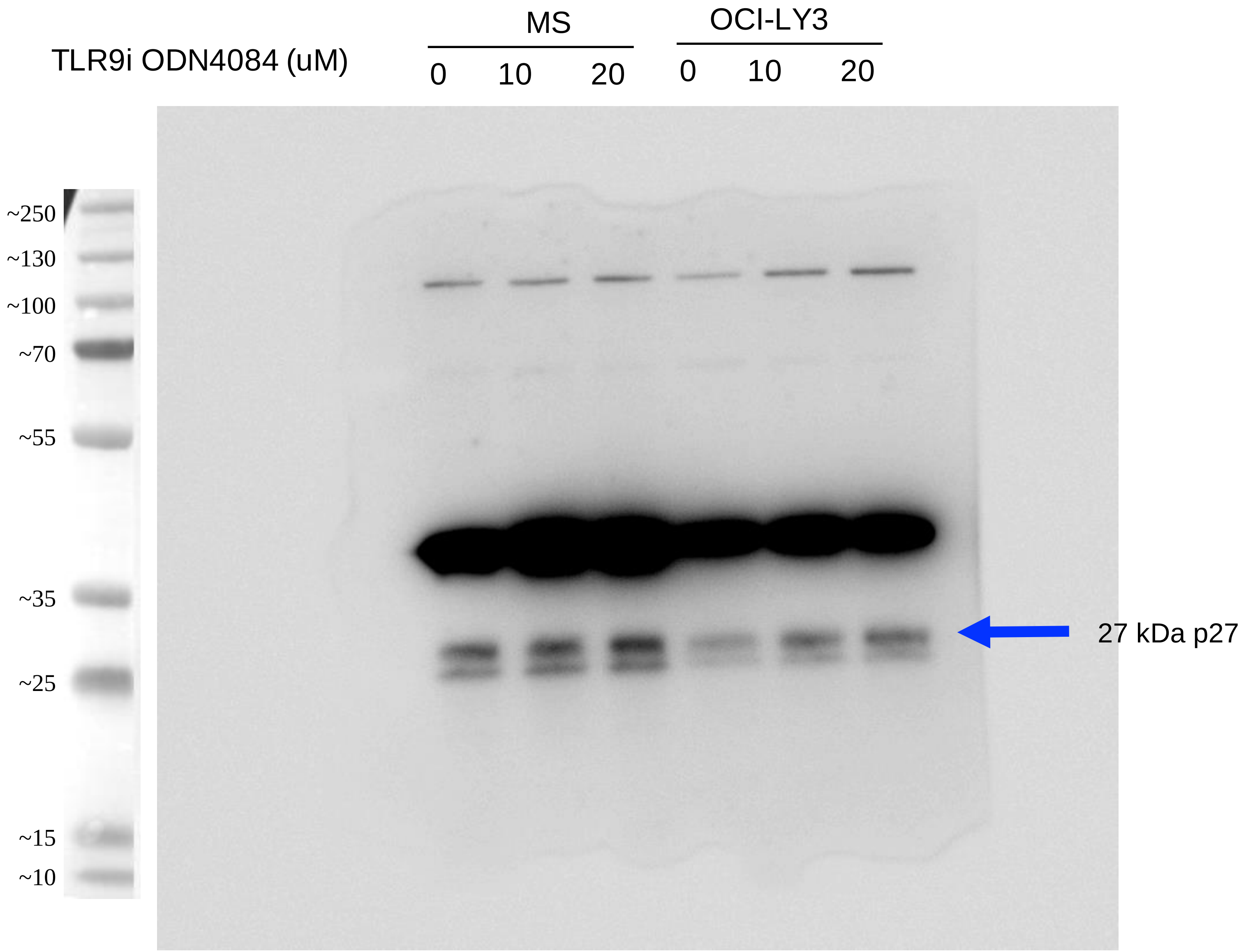

Figure S8

The crossed immunoblots refer to another project and Is not related to this paper

p27

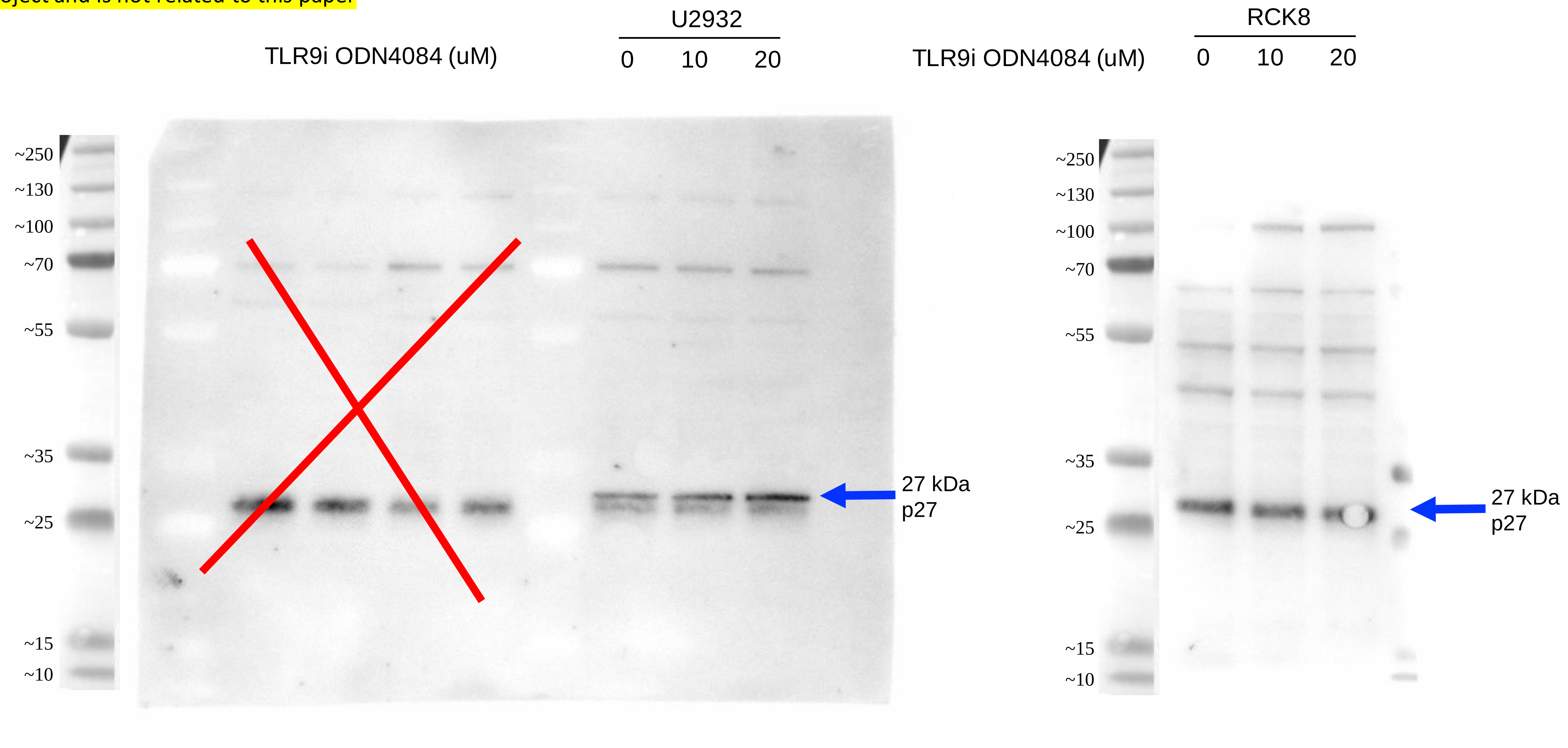

Figure S8

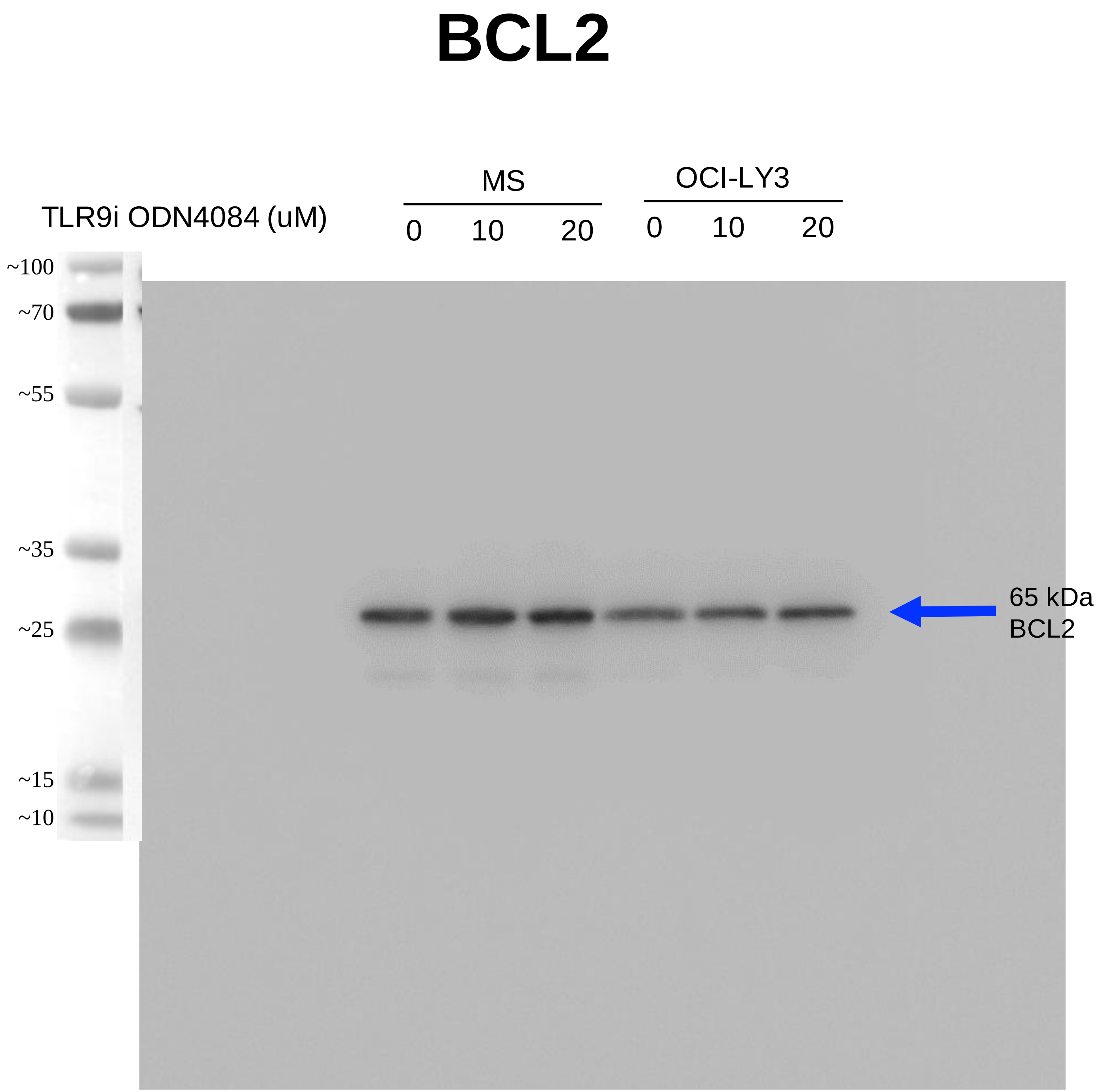

BCL2
MS
OCI-LY3
TLR9i ODN4084 (uM)
0
10
20
0
10
20
~100
~70
~55
~35
~25
~15
~10
65 kDa
BCL2

Figure S8

BCL2

The crossed immunoblots refer to another project and Is not related to this paper

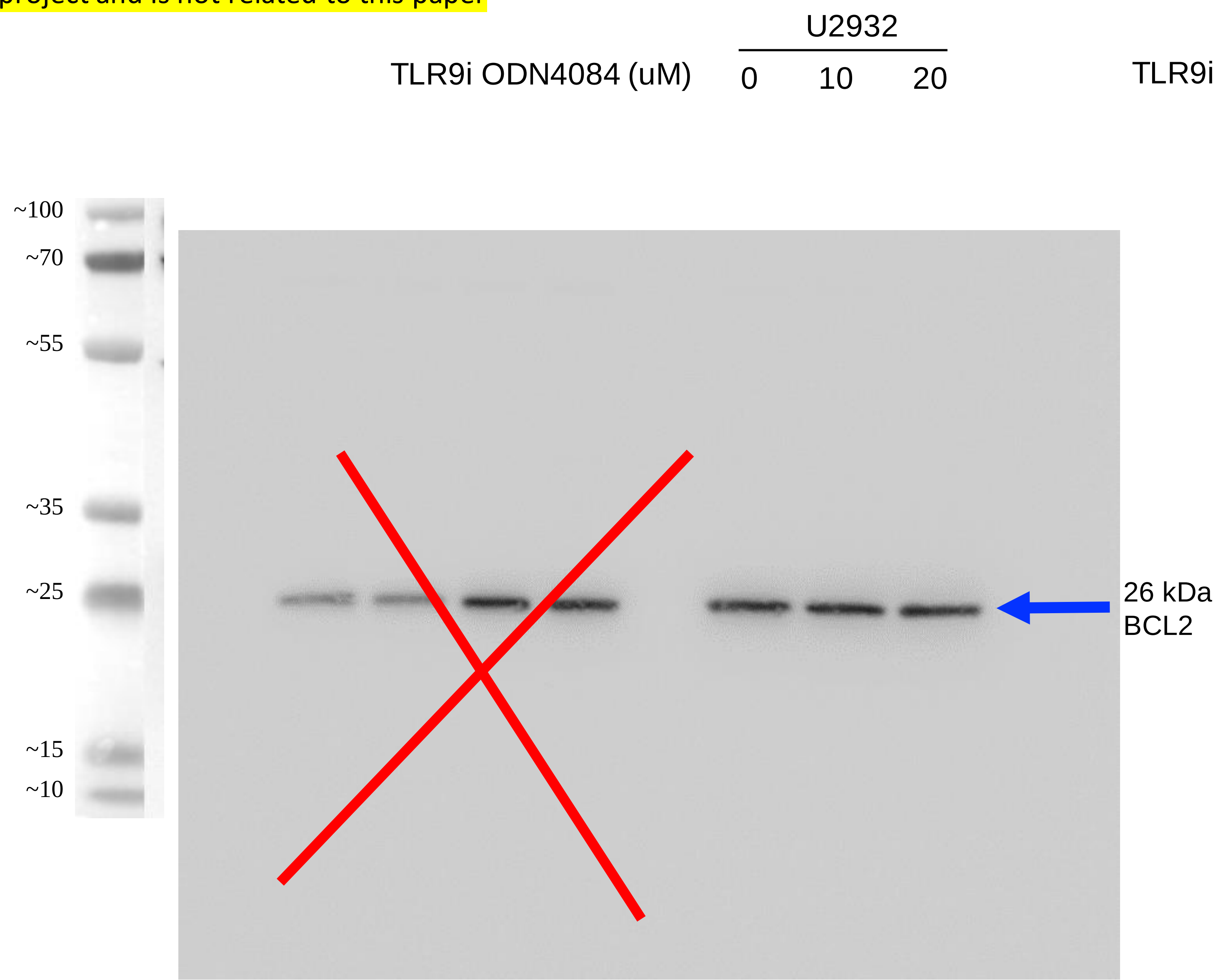

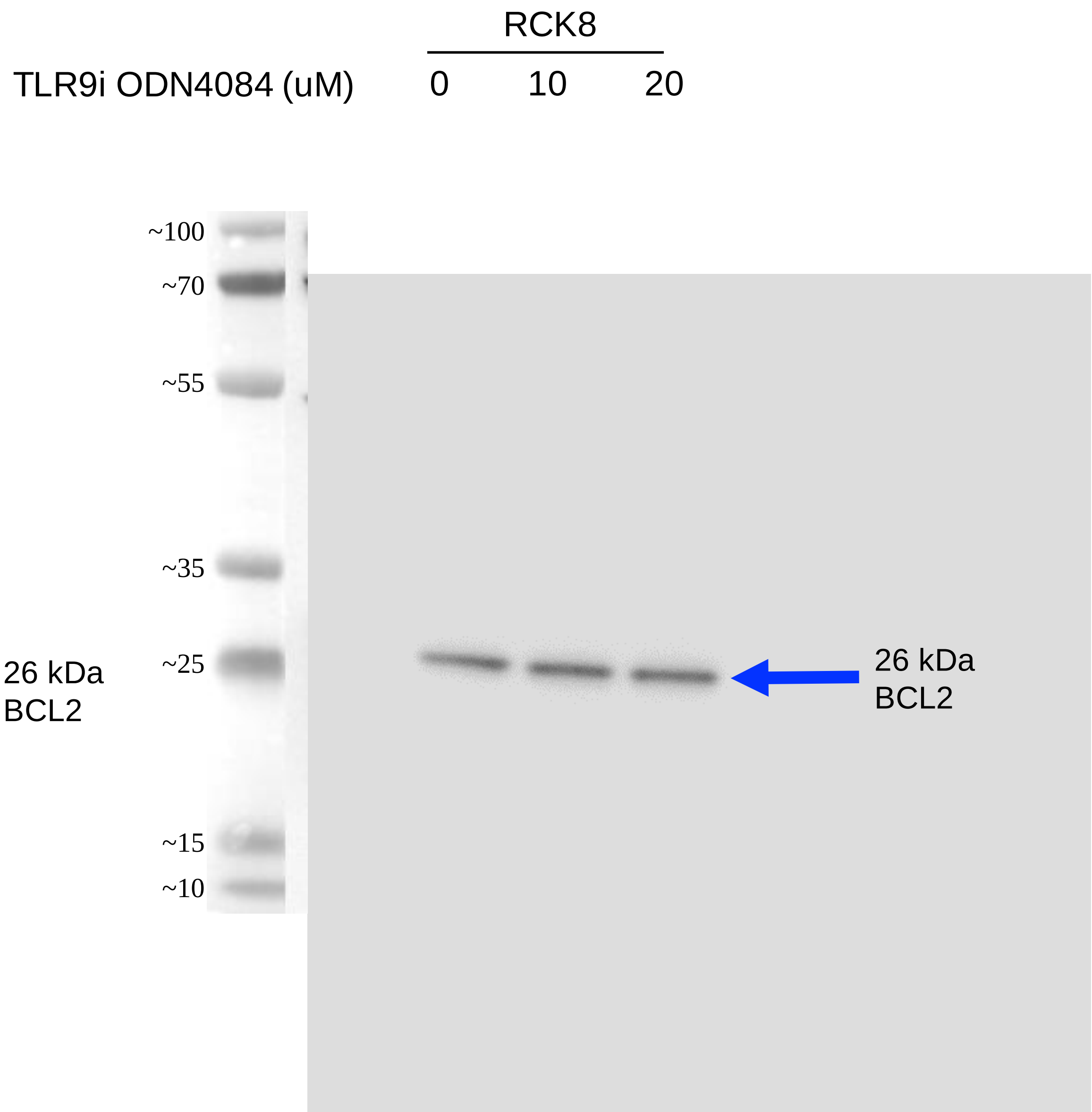

Figure S8

MCL1

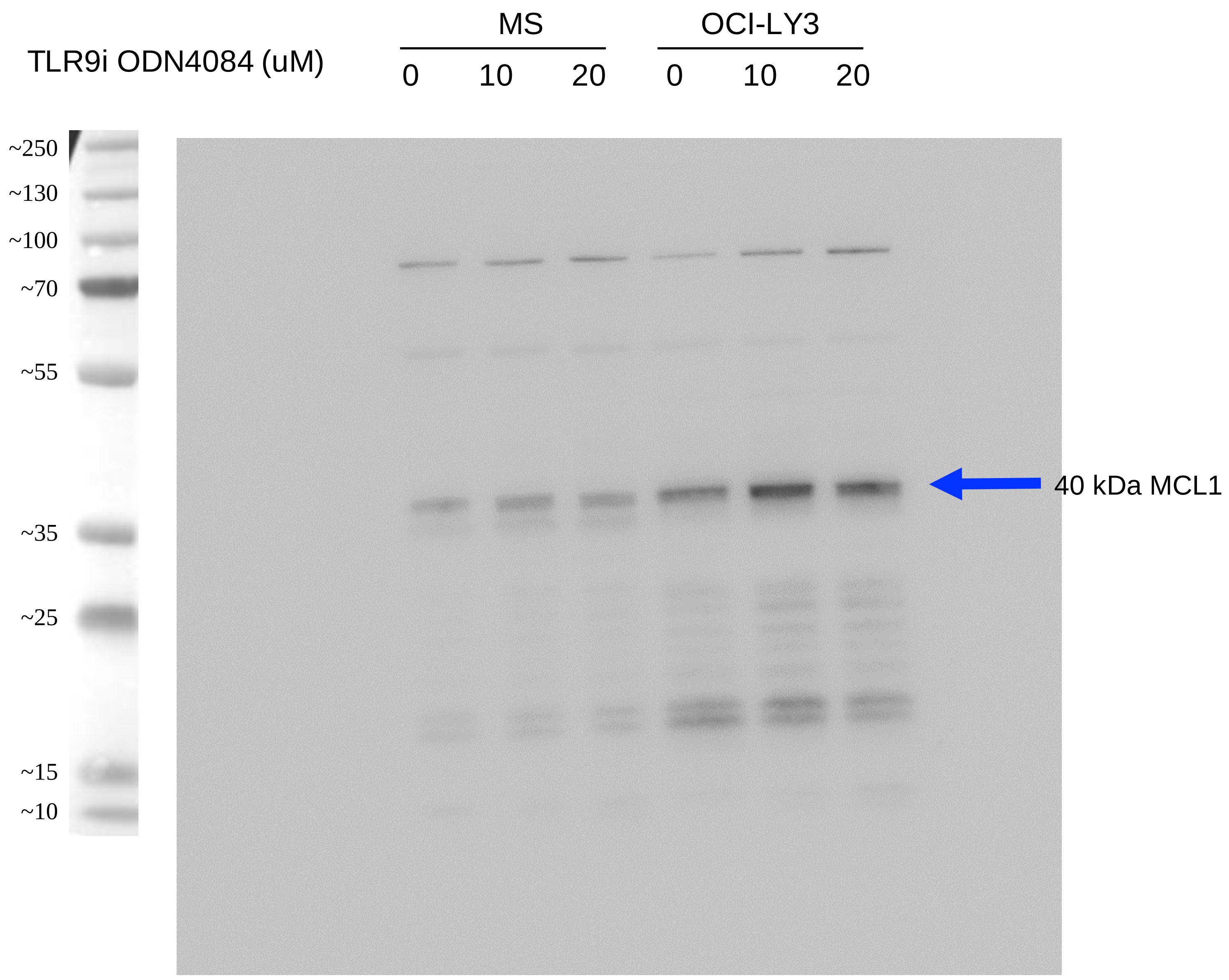

Figure S8

MCL1

The crossed immunoblots refer to another project and Is not related to this paper

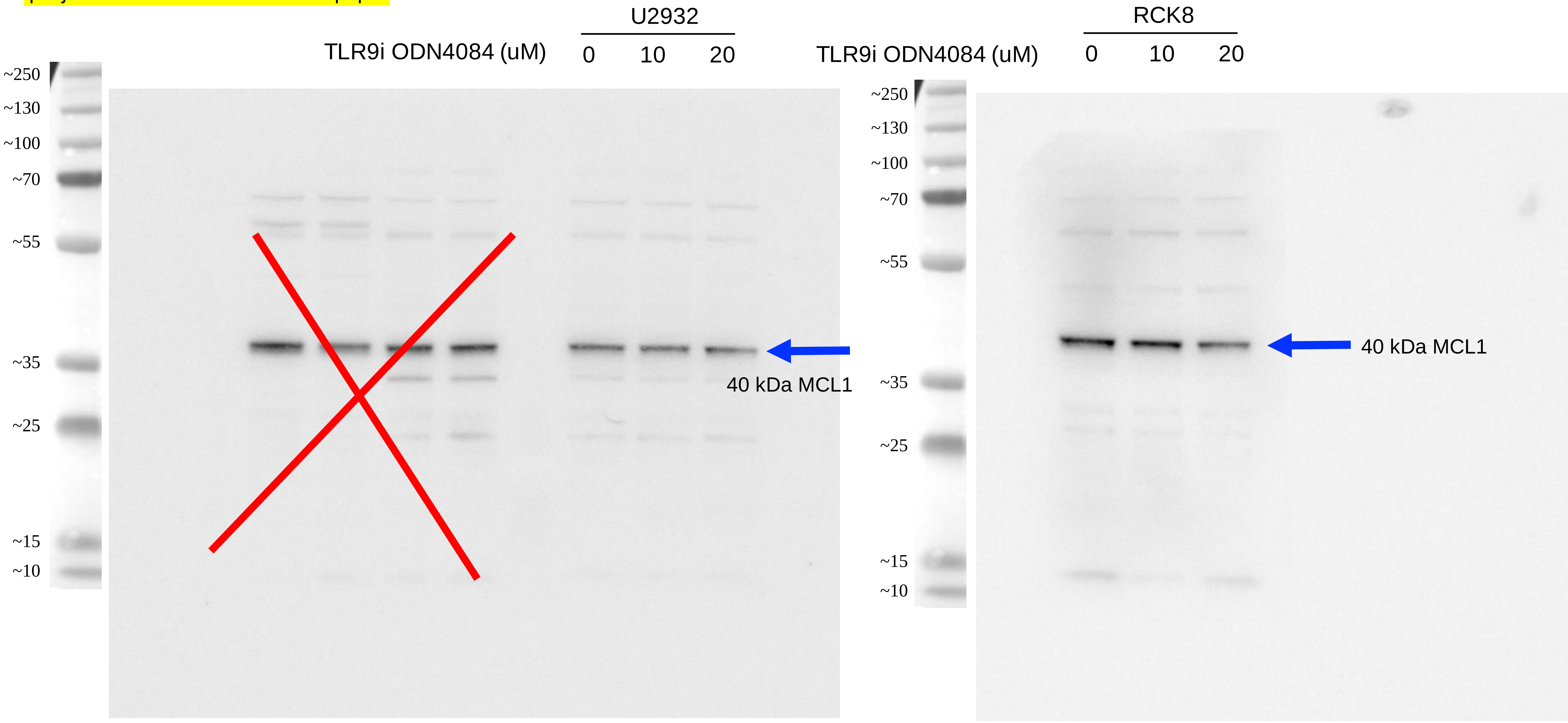

Figure S8

GAPDH

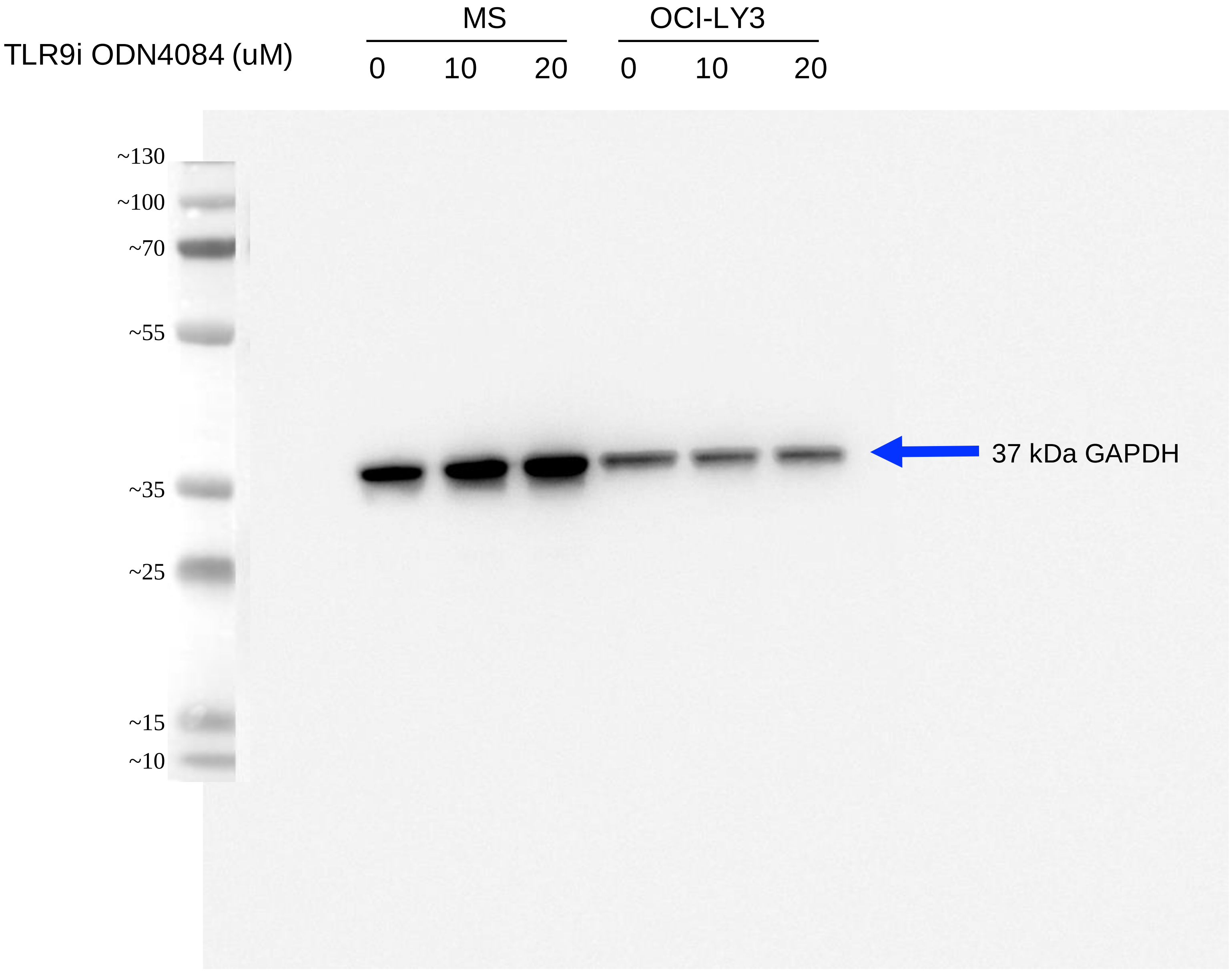

Figure S8

GAPDH (longer exp.)

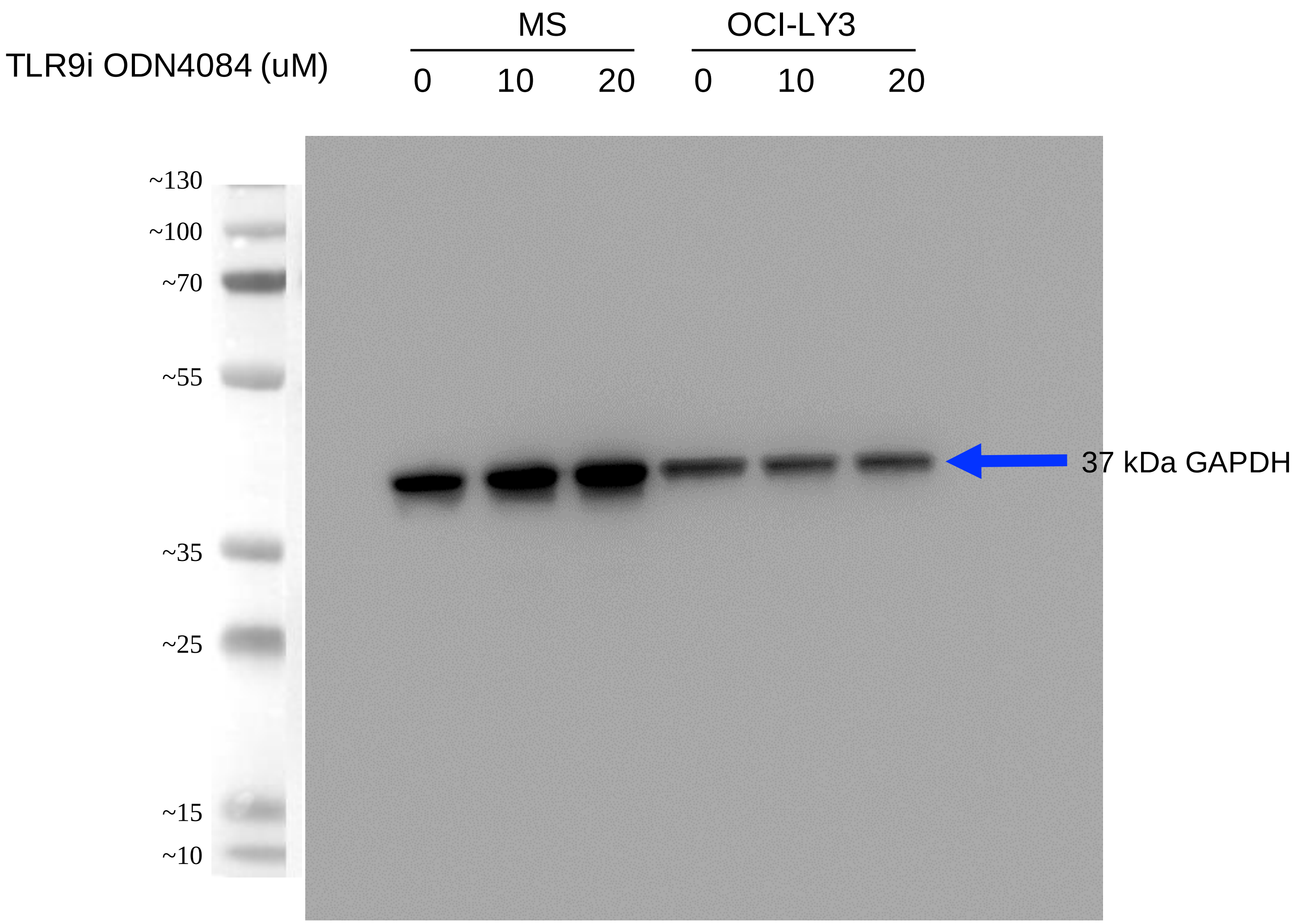

Figure S8

GAPDH

The crossed immunoblots refer to another project and Is not related to this paper

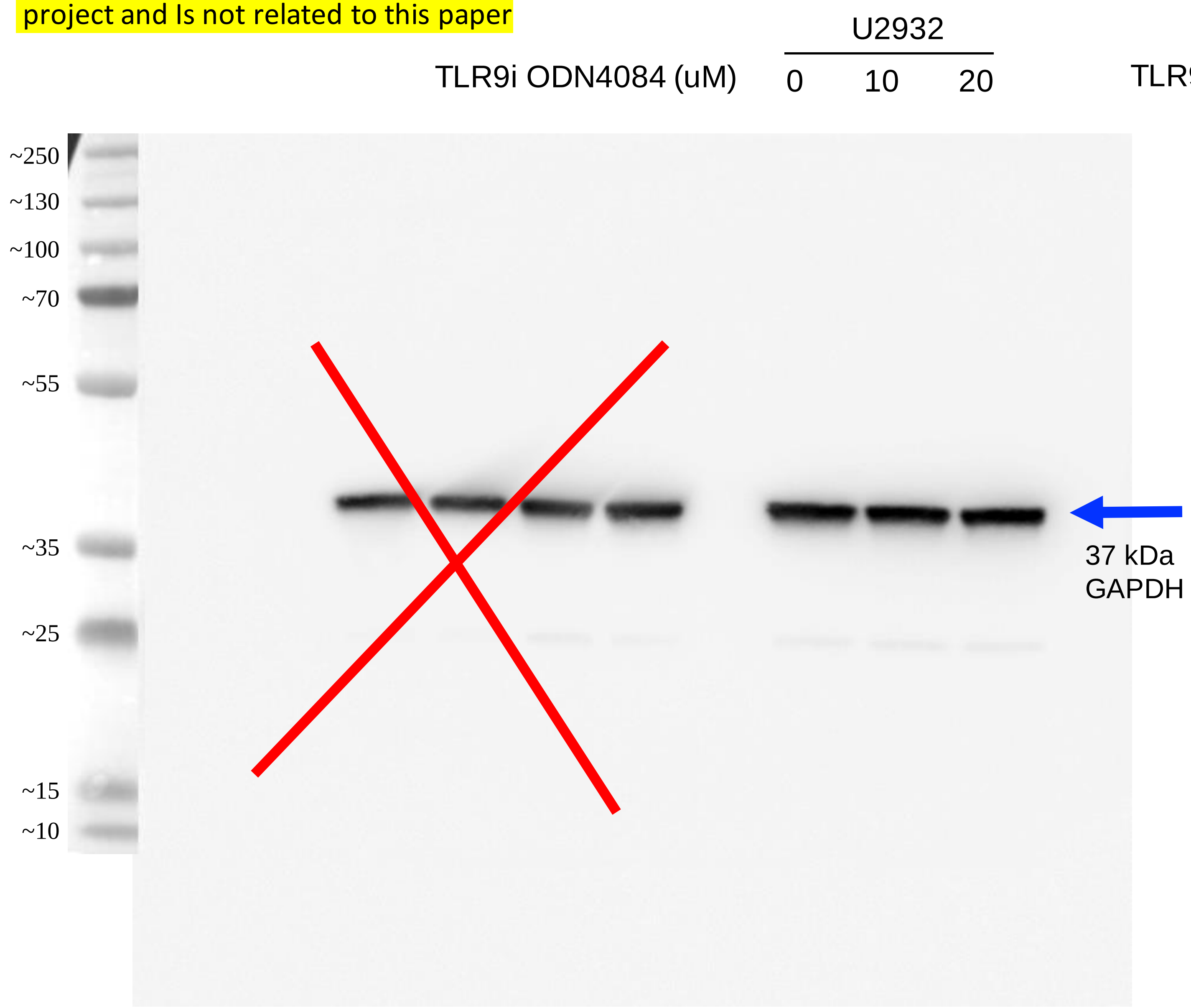

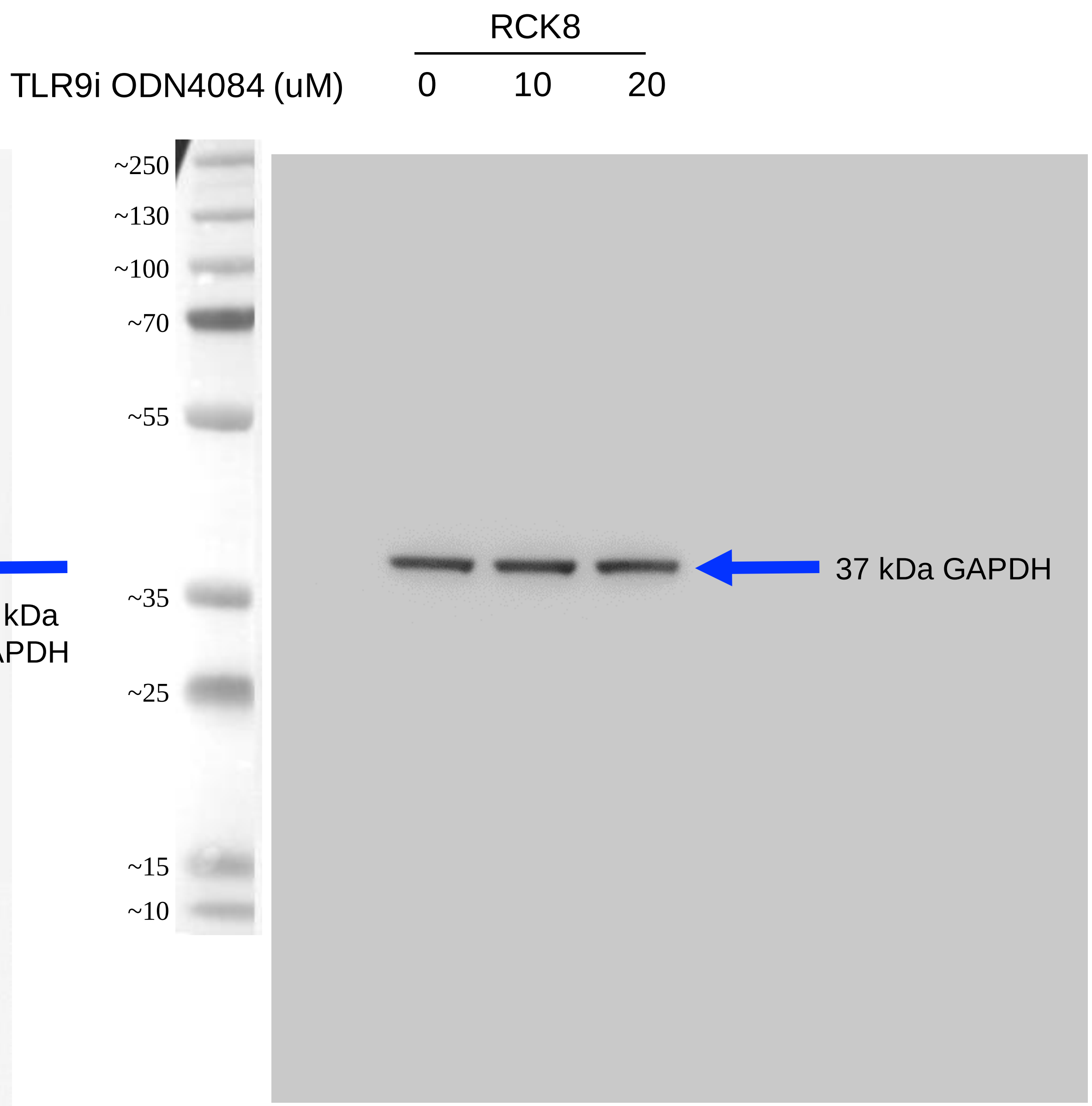

Figure S8

GAPDH

The crossed immunoblots refer to another project and Is not related to this paper

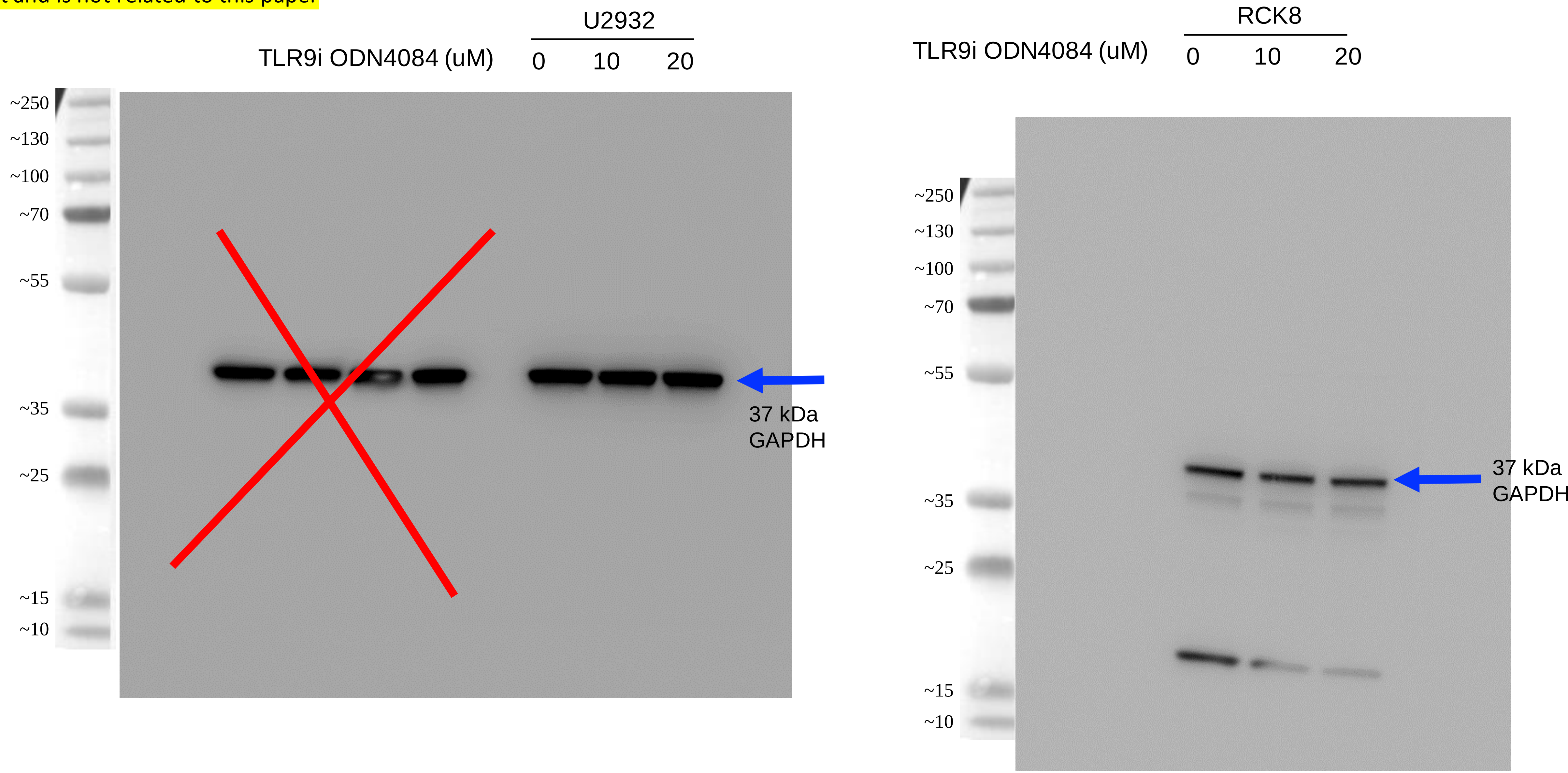

Supplement: Supplementary file 4 — Supplementary Material 4. [file 12885_2025_14359_MOESM4_ESM.zip › Raw data for Fig 2 and S8_merged_pdf_REV4.pdf]
