# Supplementary material for: Toll-like receptor 9 (TLR9) expression correlates with cell of origin and predicts clinical outcome in diffuse large B-cell lymphoma
Source: BMC Cancer. 2025 May 28;25:959. doi: 10.1186/s12885-025-14359-7 (PMC12117956; doi:10.1186/s12885-025-14359-7)

Figure S8

TLR9

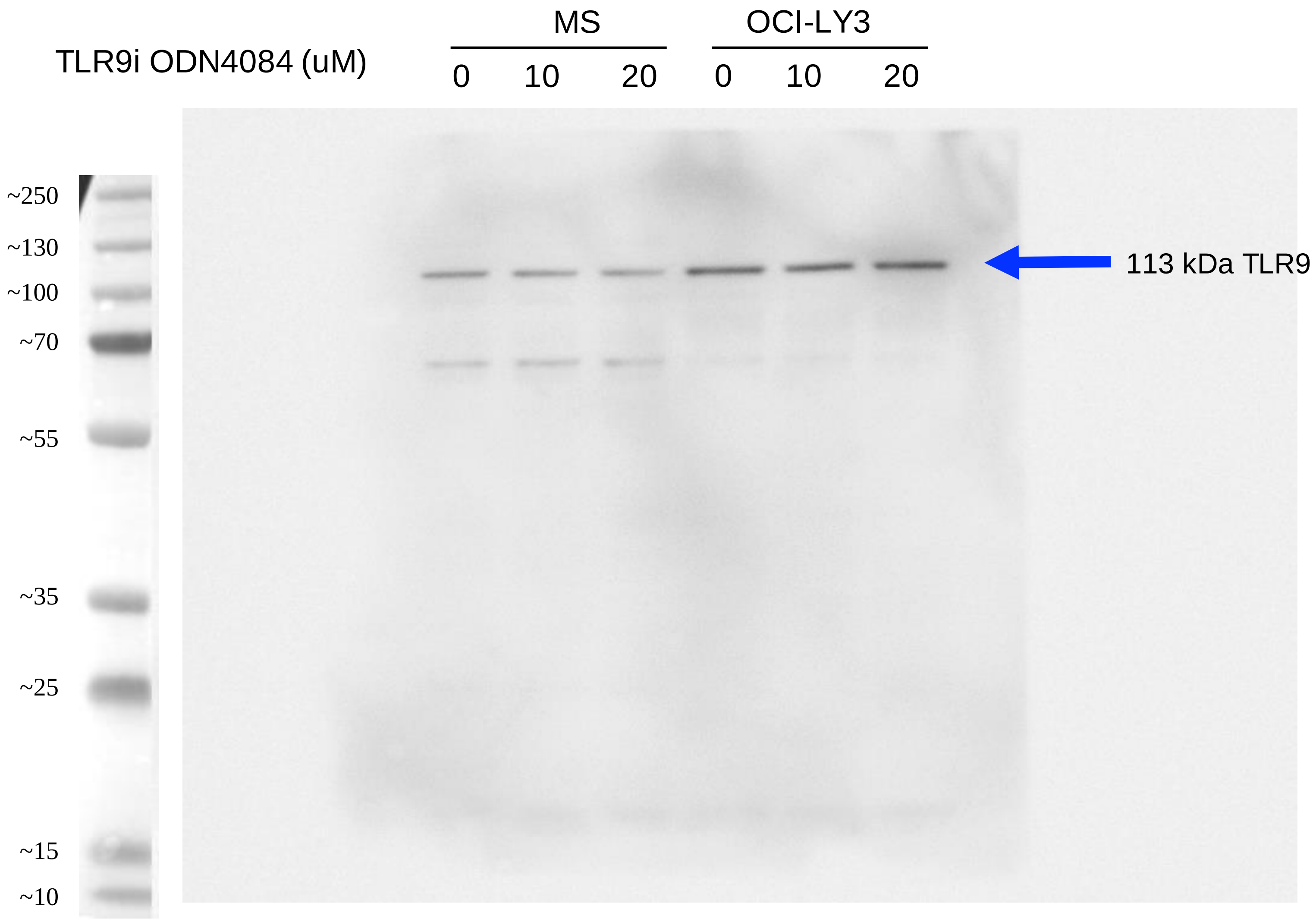

Figure S8

TLR9

The crossed immunoblots refer to another project and Is not related to this paper

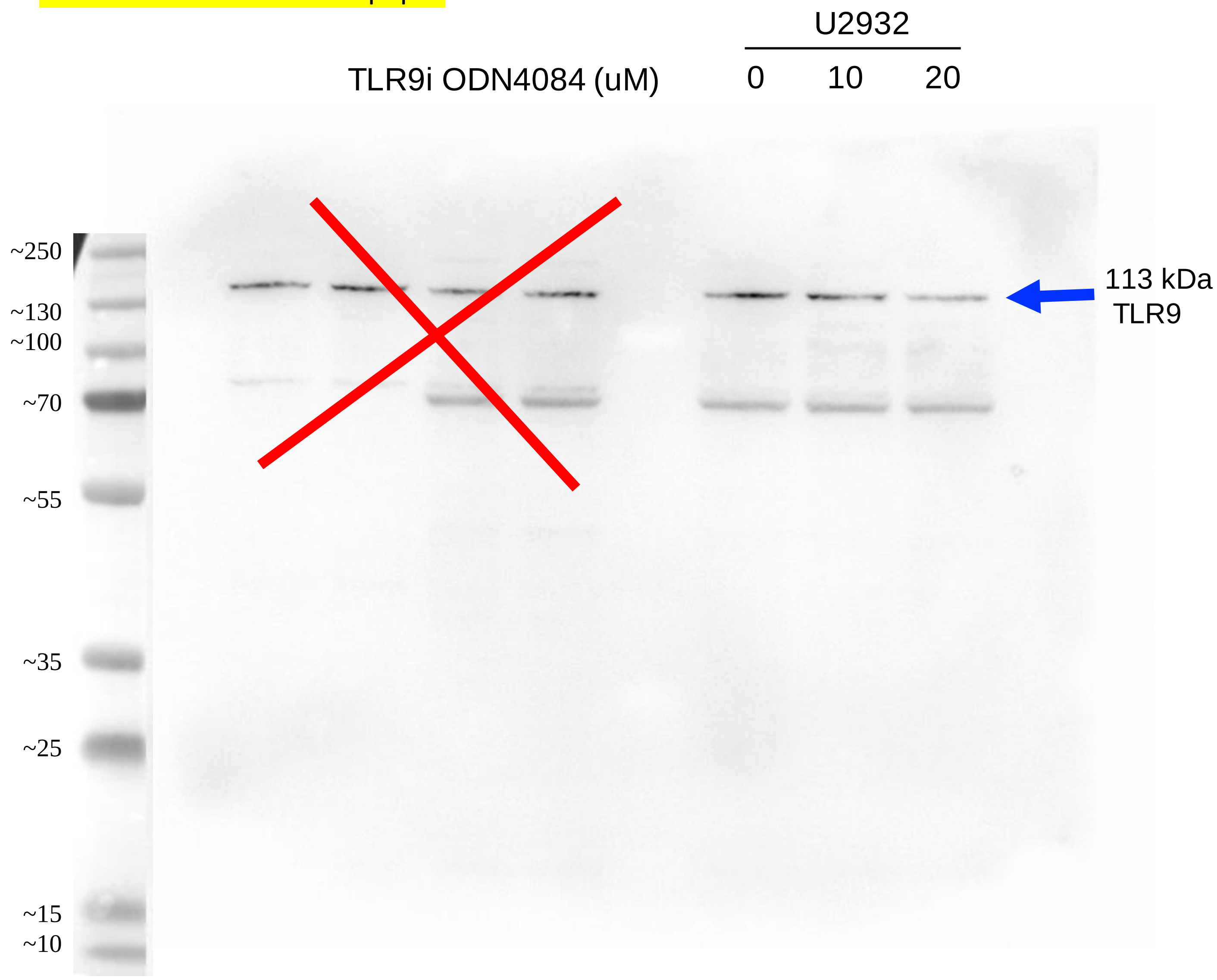

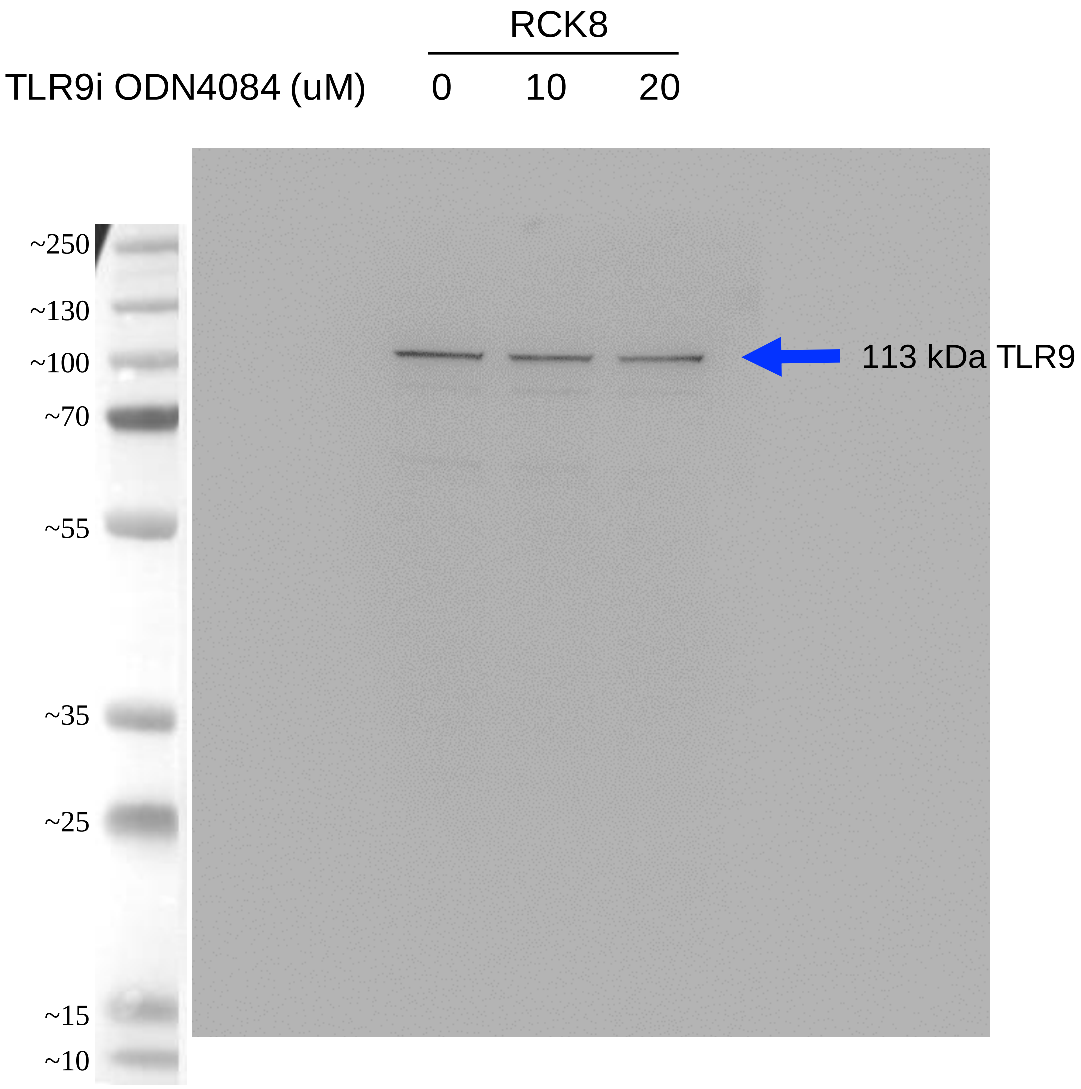

Figure S8

pNFκB p65

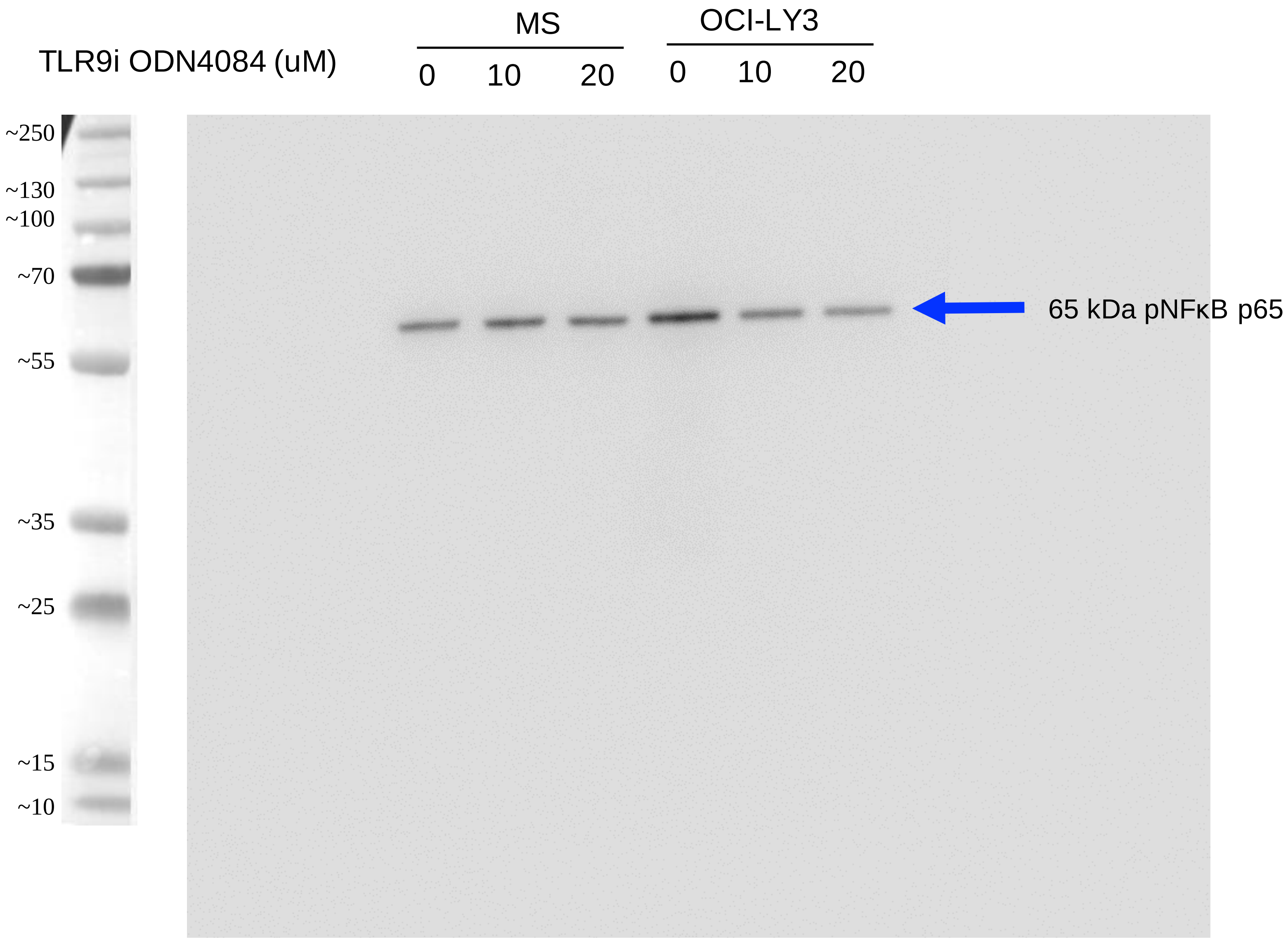

Figure S8

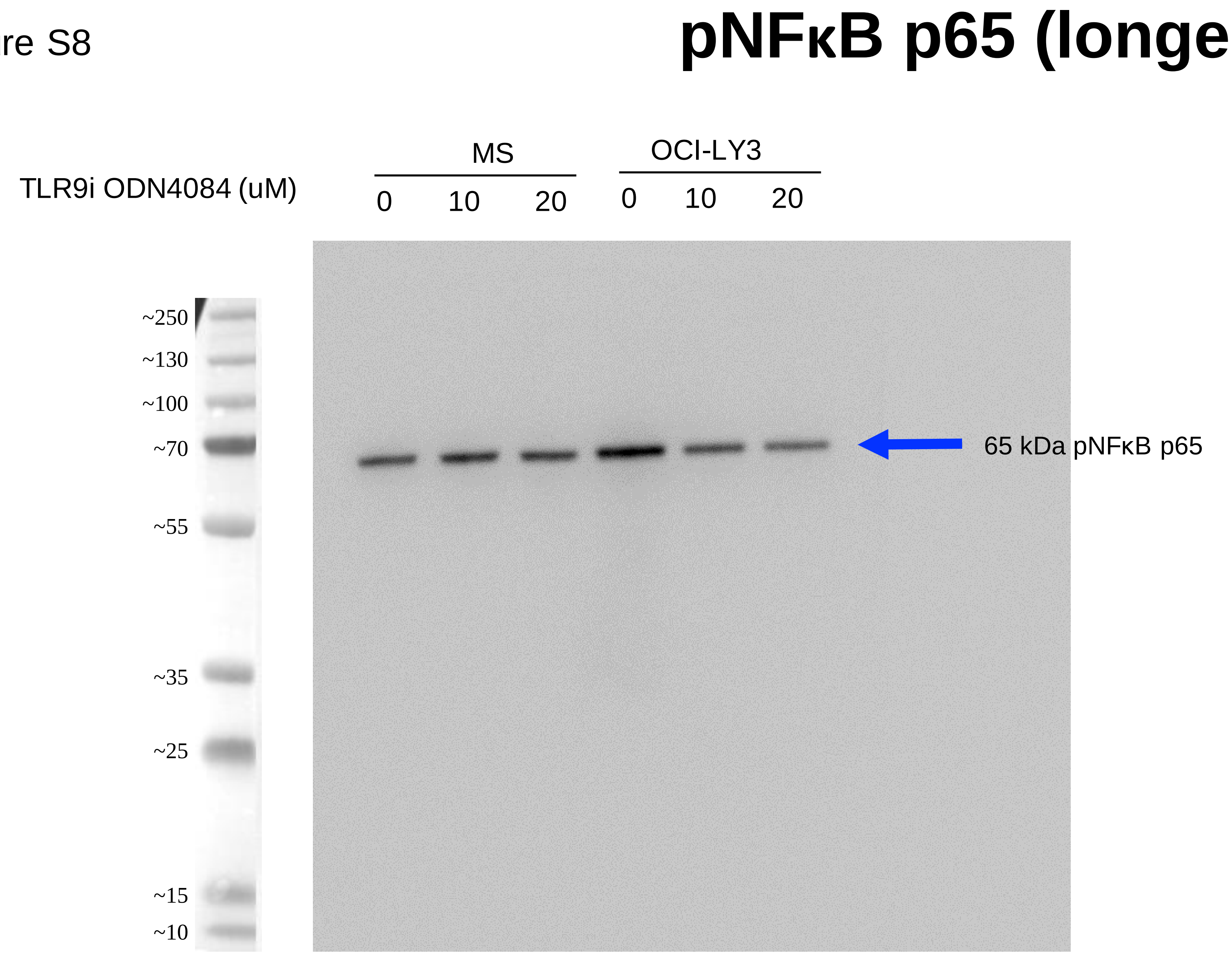
pNFκB p65 (longer exp.)
MS
OCI-LY3
TLR9i ODN4084 (uM)
0
10
20
0
10
20
~250
~130
~100
~70
~55
~35
~25
~15
~10
65 kDa pNFκB p65

Figure S8

pNFκB p65

The crossed immunoblots refer to another project and Is not related to this paper

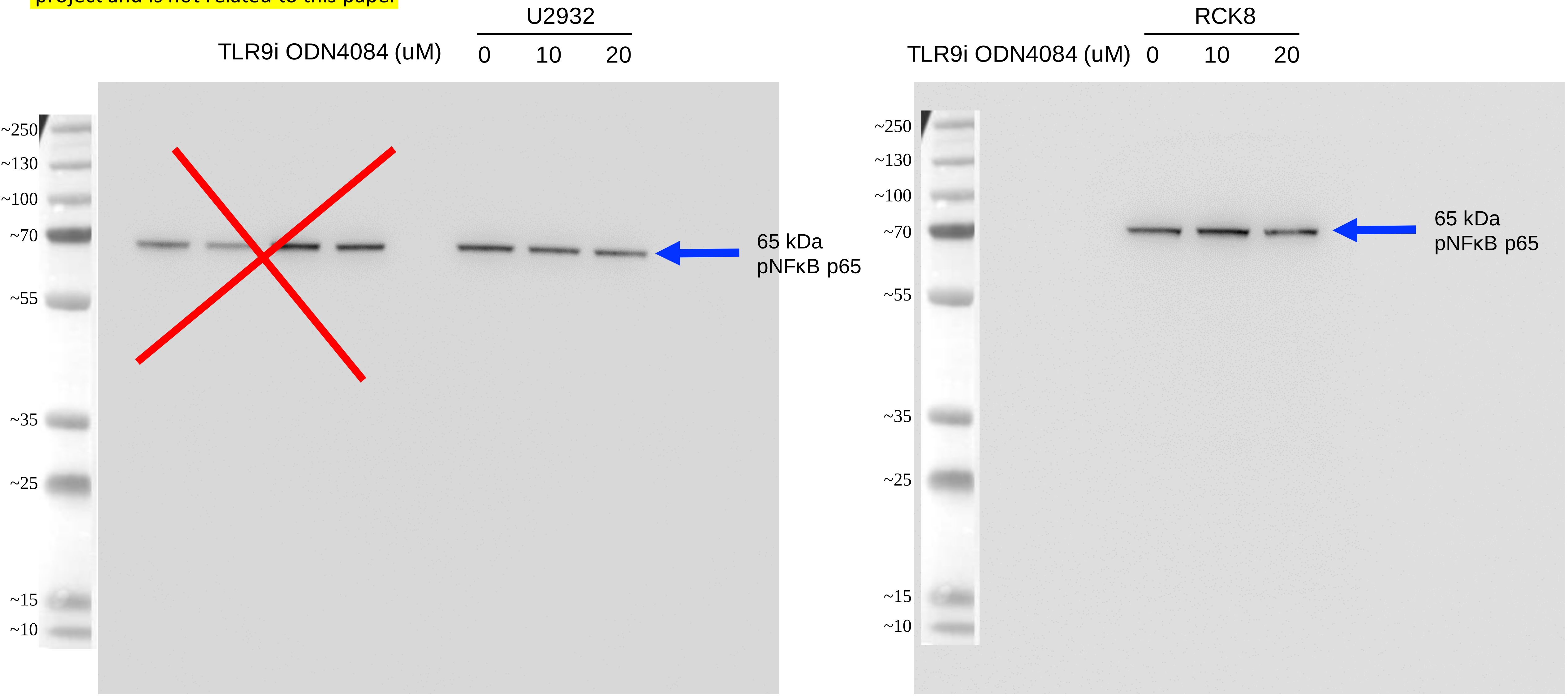

Figure S8

pNFκB p65 (longer exp.)

The crossed immunoblots refer to another project and Is not related to this paper

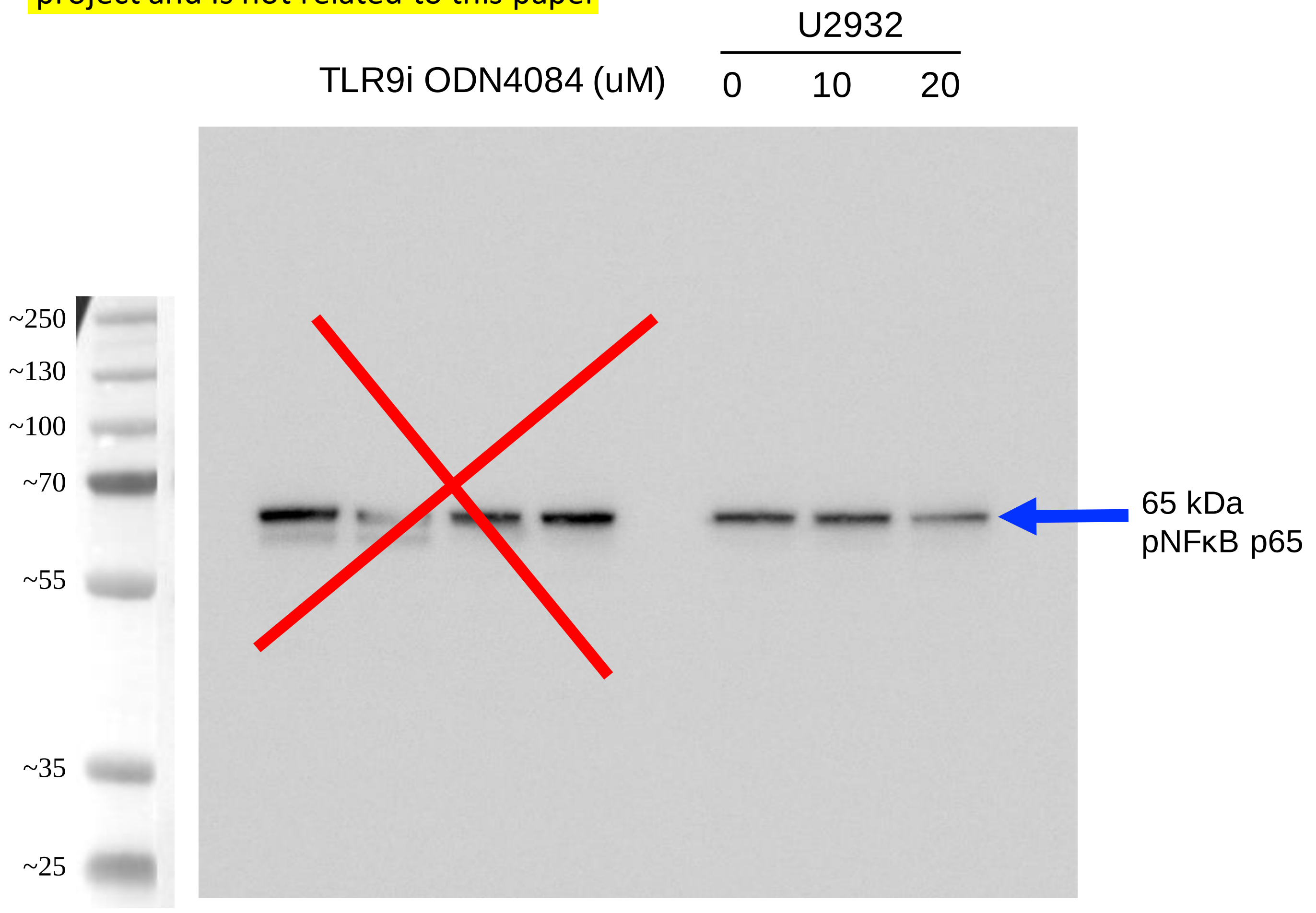

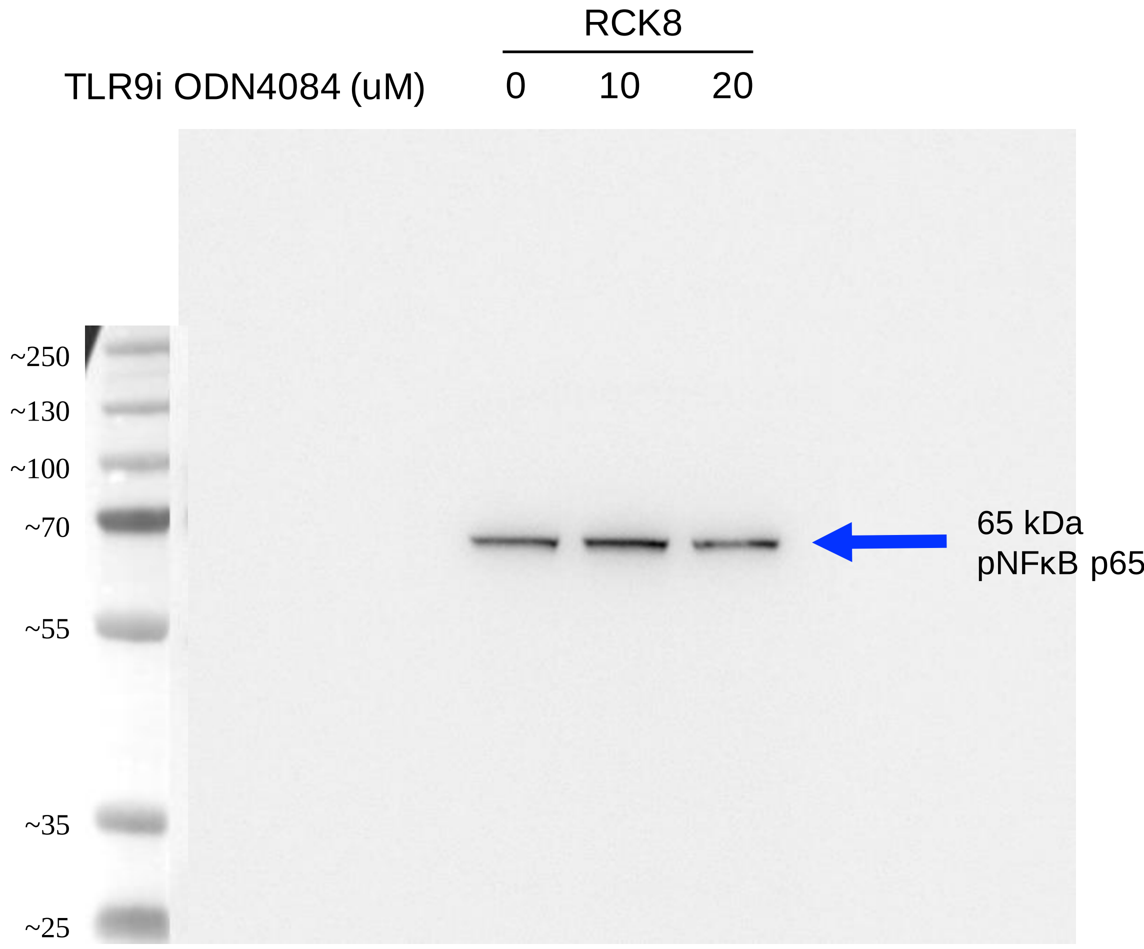

Figure S8

NFκB p65

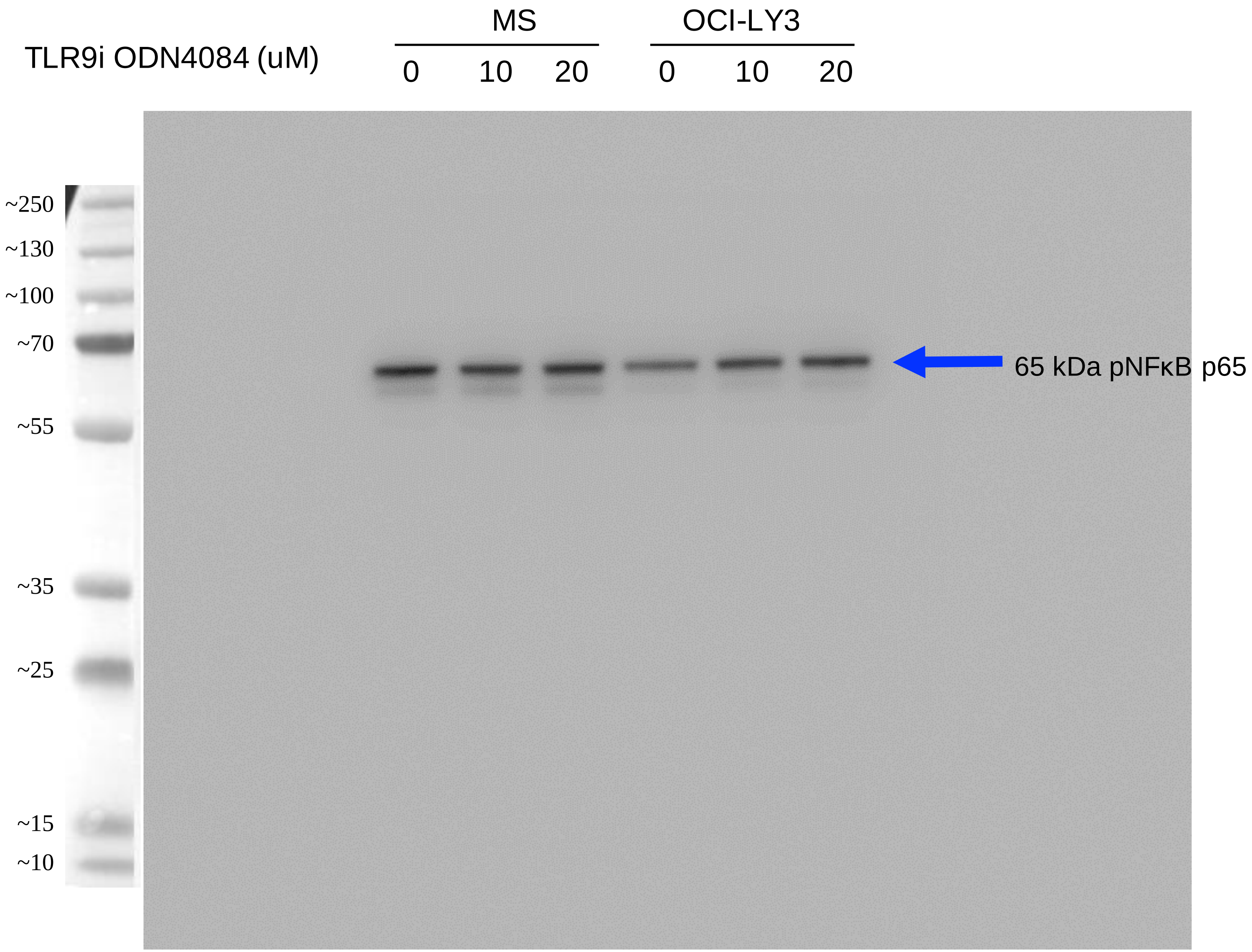

Figure S8

NFκB p65

The crossed immunoblots refer to another project and Is not related to this paper

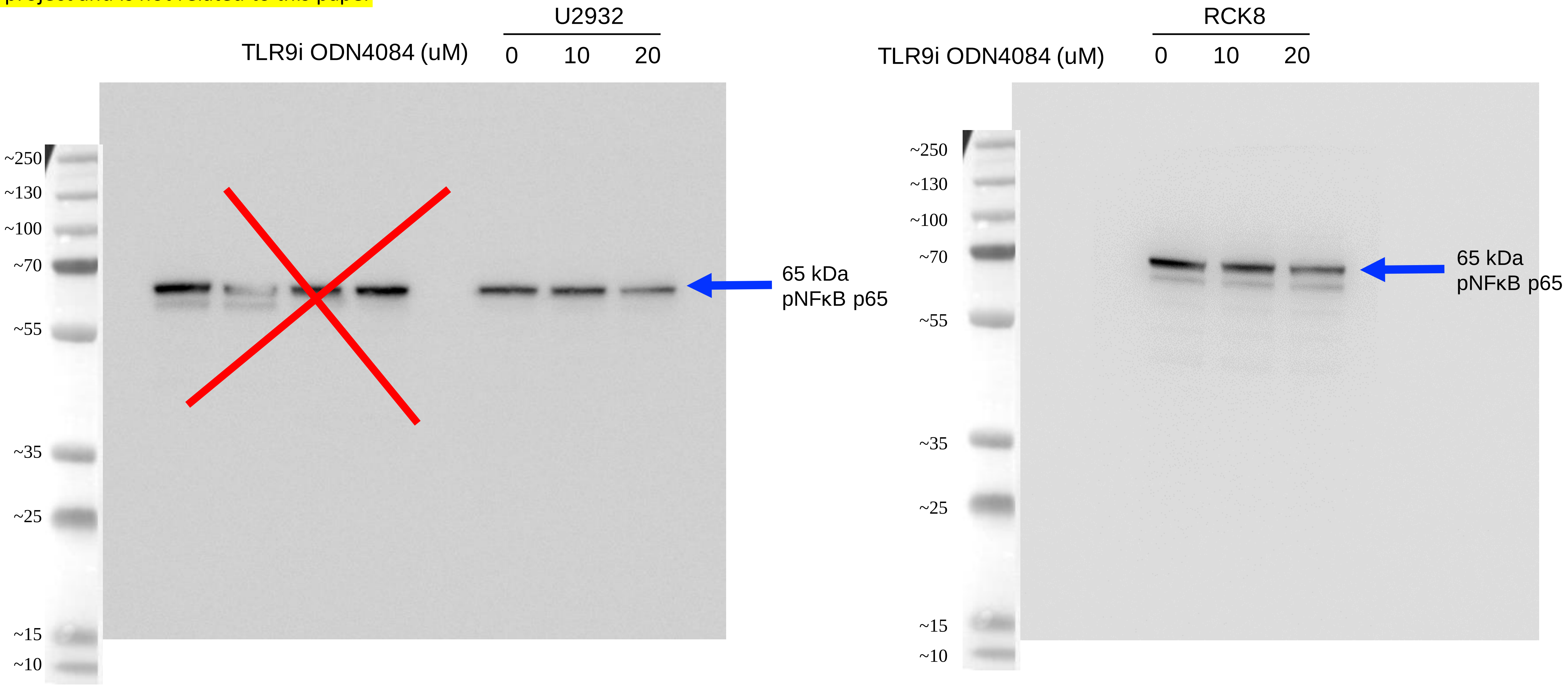

Figure S8

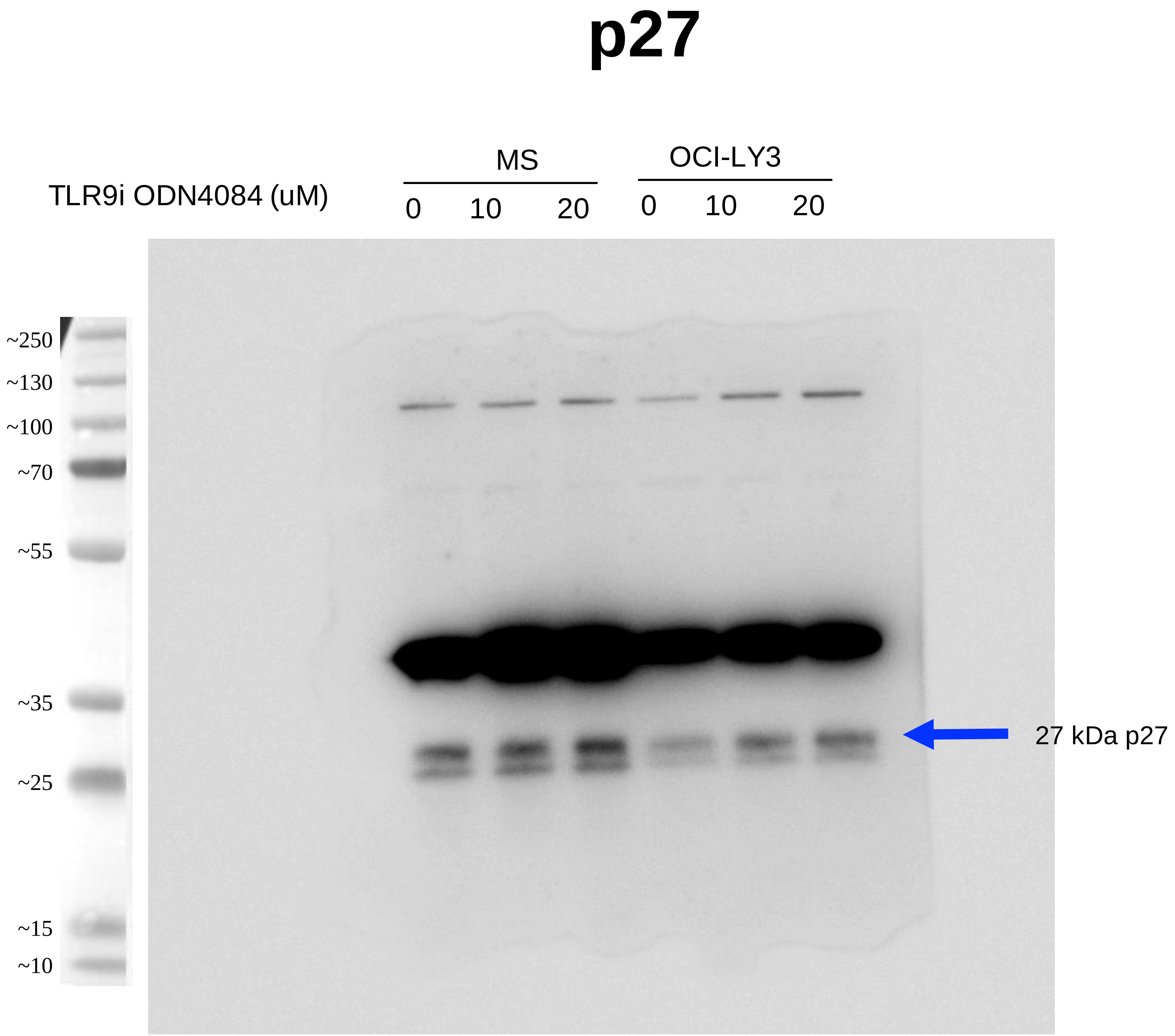

p27
MS
OCI-LY3
TLR9i ODN4084 (uM)
0
10
20
0
10
20
~250
~130
~100
~70
~55
~35
~25
~15
~10
27 kDa p27

Figure S8

p27

The crossed immunoblots refer to another project and Is not related to this paper

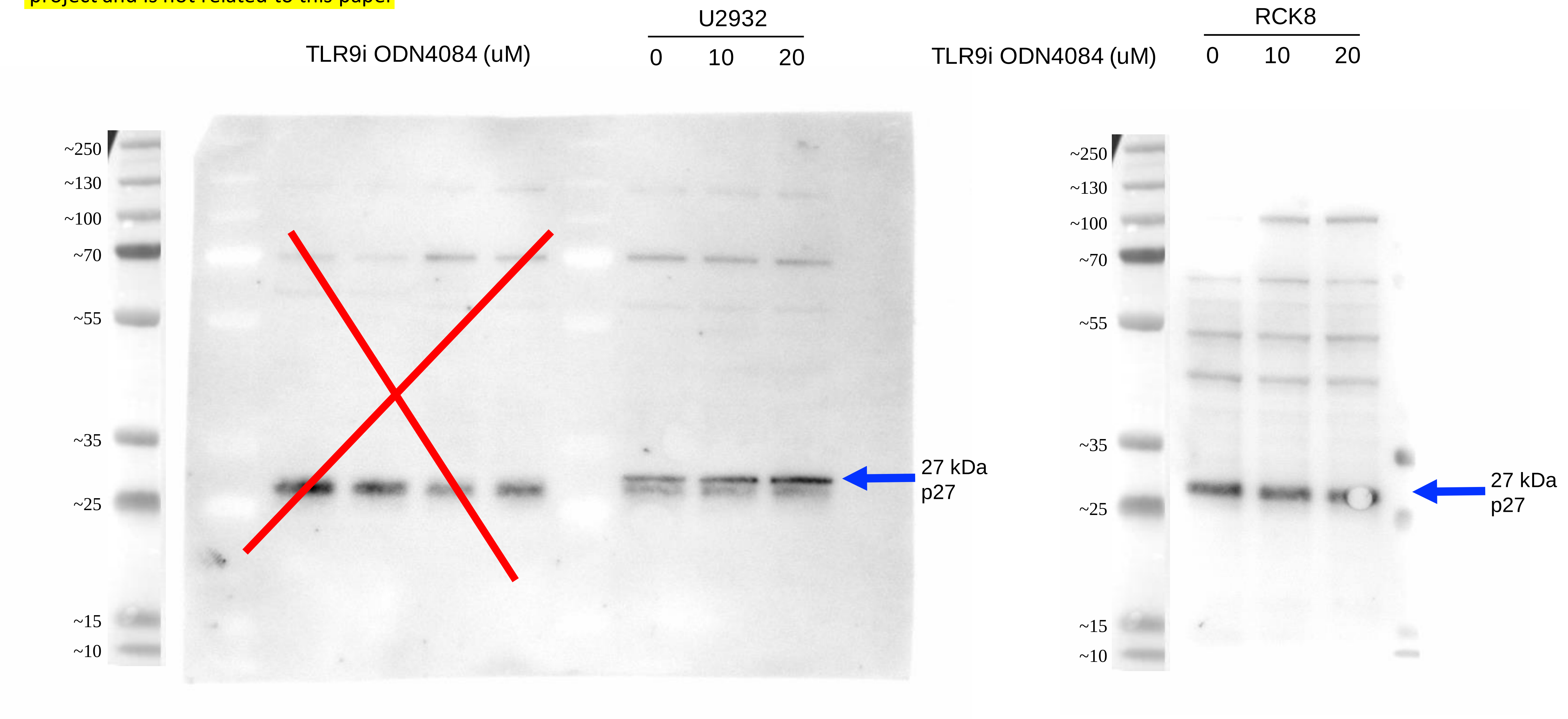

Figure S8

BCL2

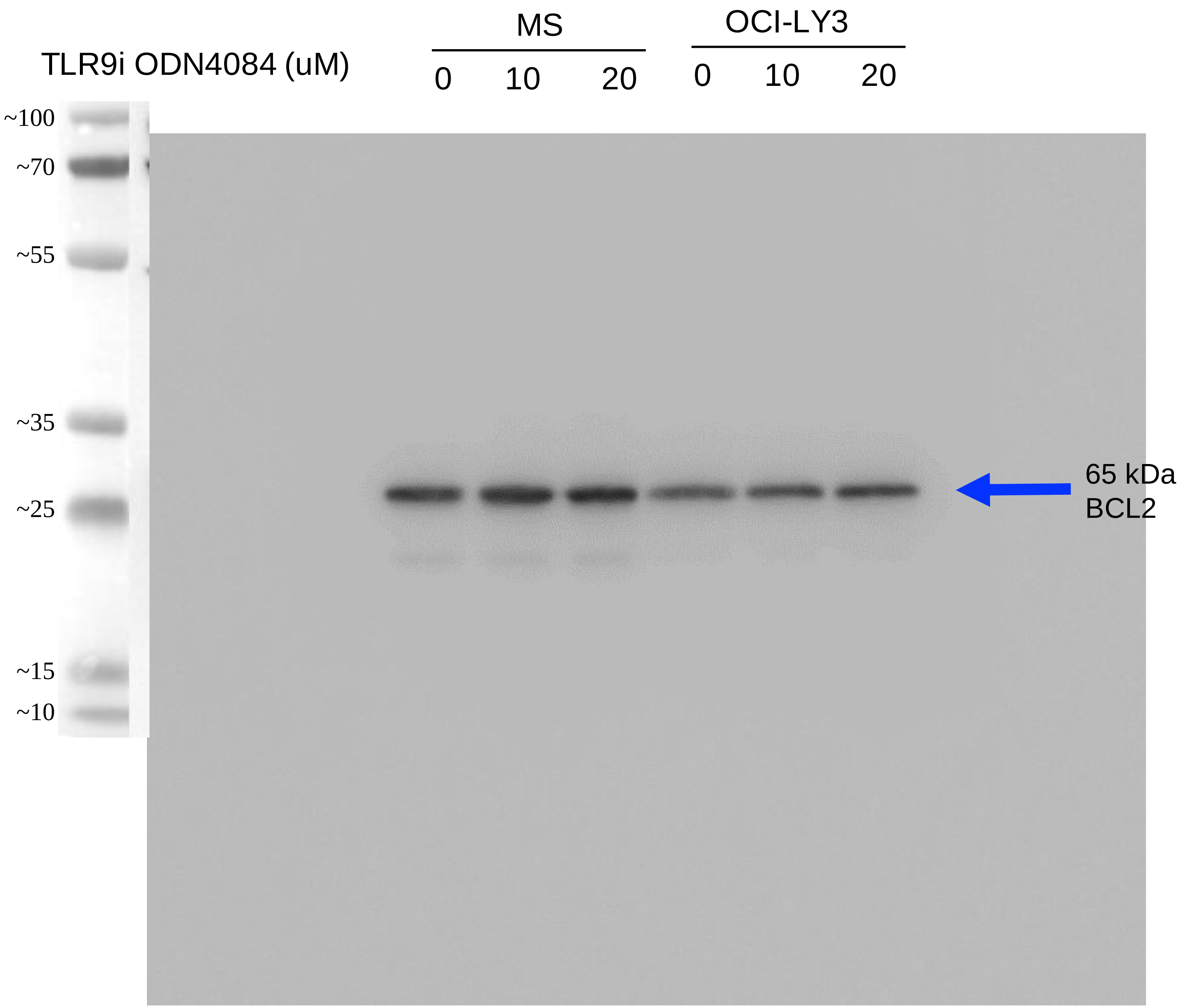

Figure S8

BCL2

The crossed immunoblots refer to another project and Is not related to this paper

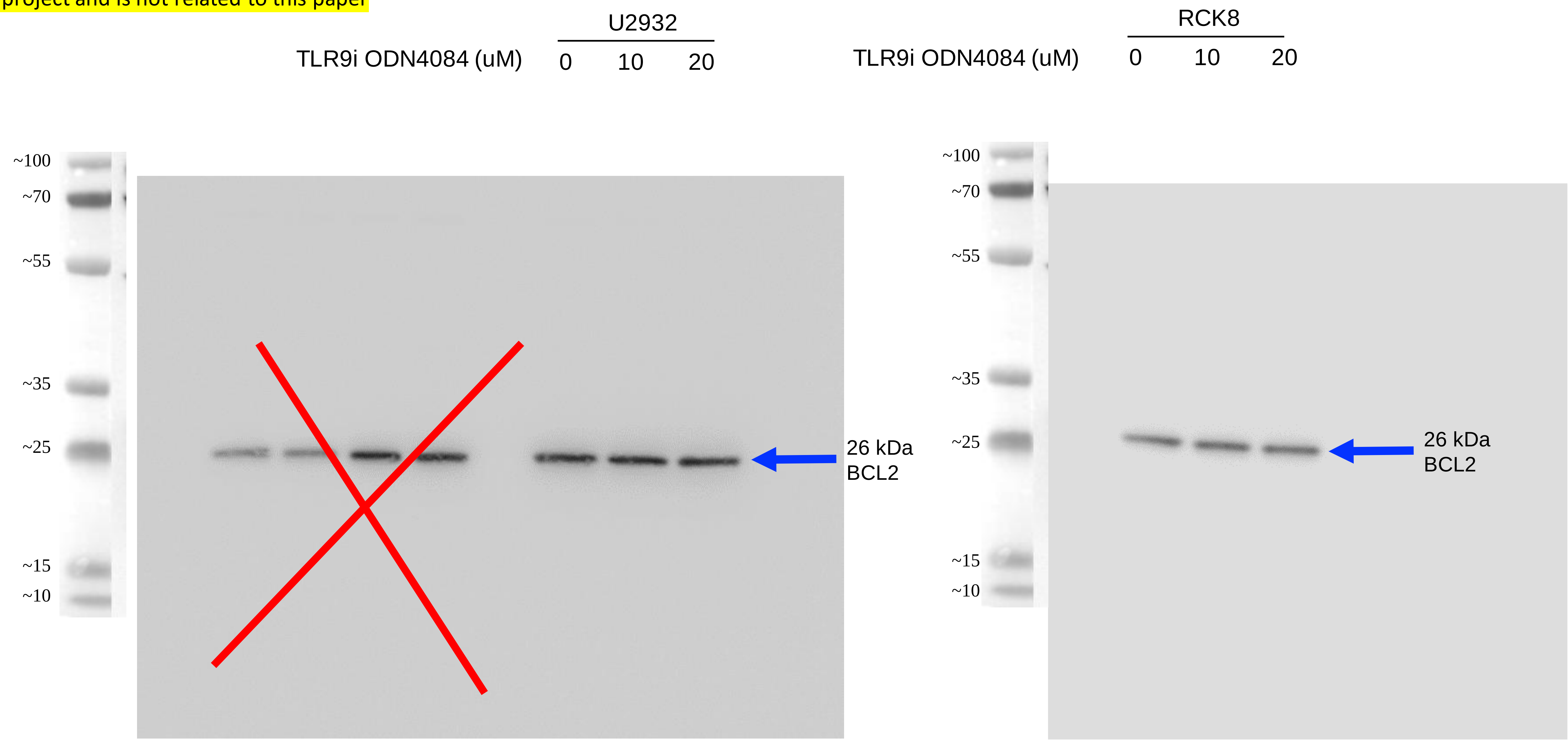

Figure S8

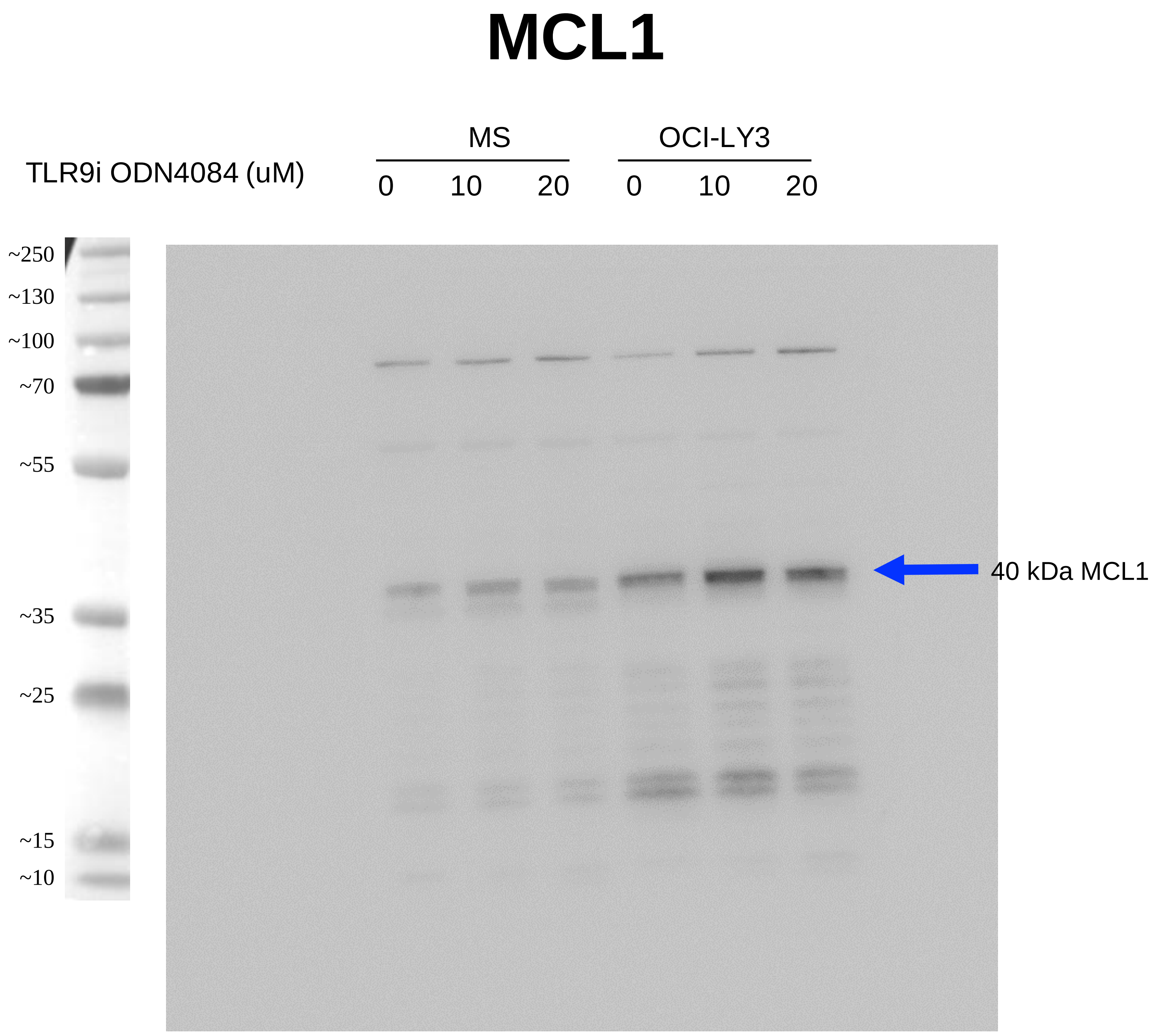
MCL1
MS
OCI-LY3
TLR9i ODN4084 (uM)
0
10
20
0
10
20
~250
~130
~100
~70
~55
~35
~25
~15
~10
40 kDa MCL1

Figure S8

MCL1

The crossed immunoblots refer to another project and Is not related to this paper

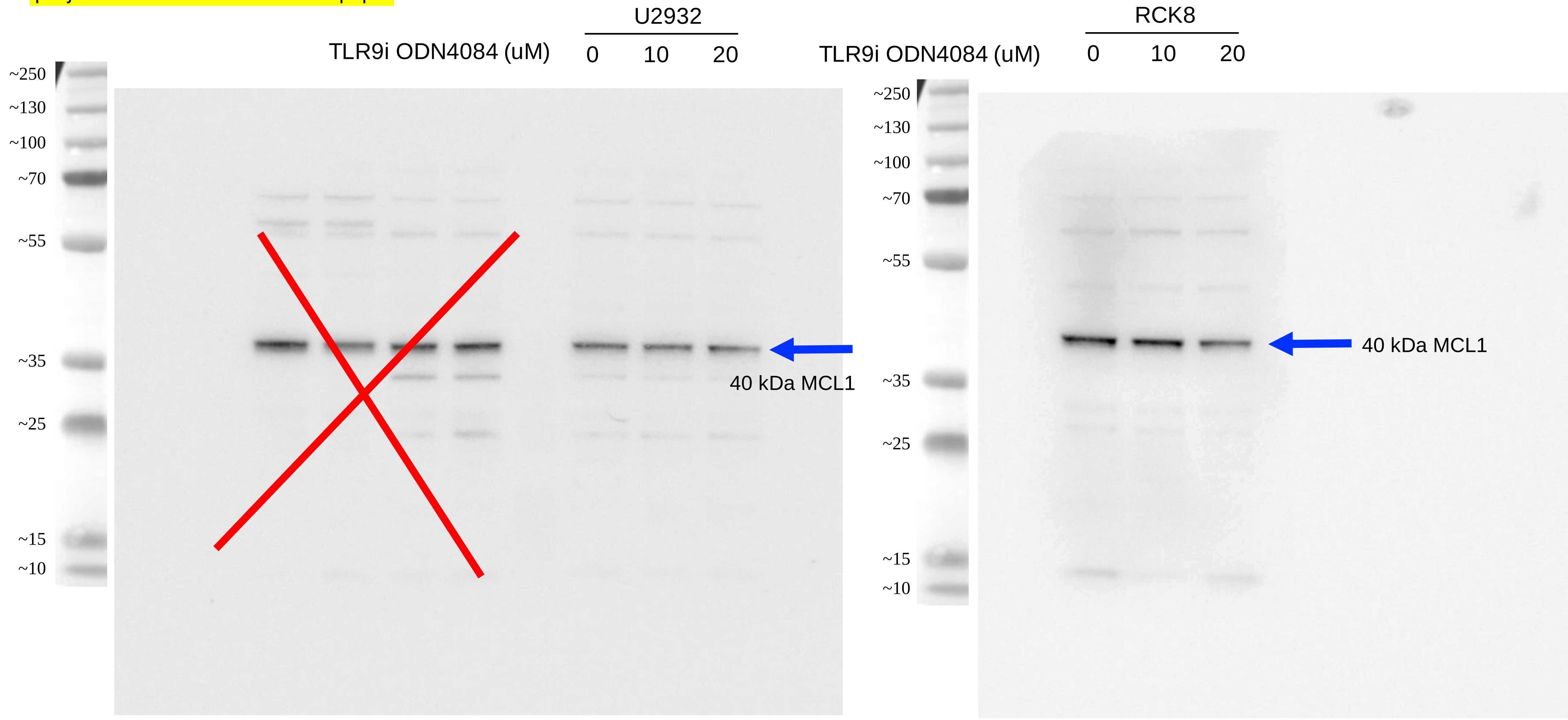

Figure S8

GAPDH

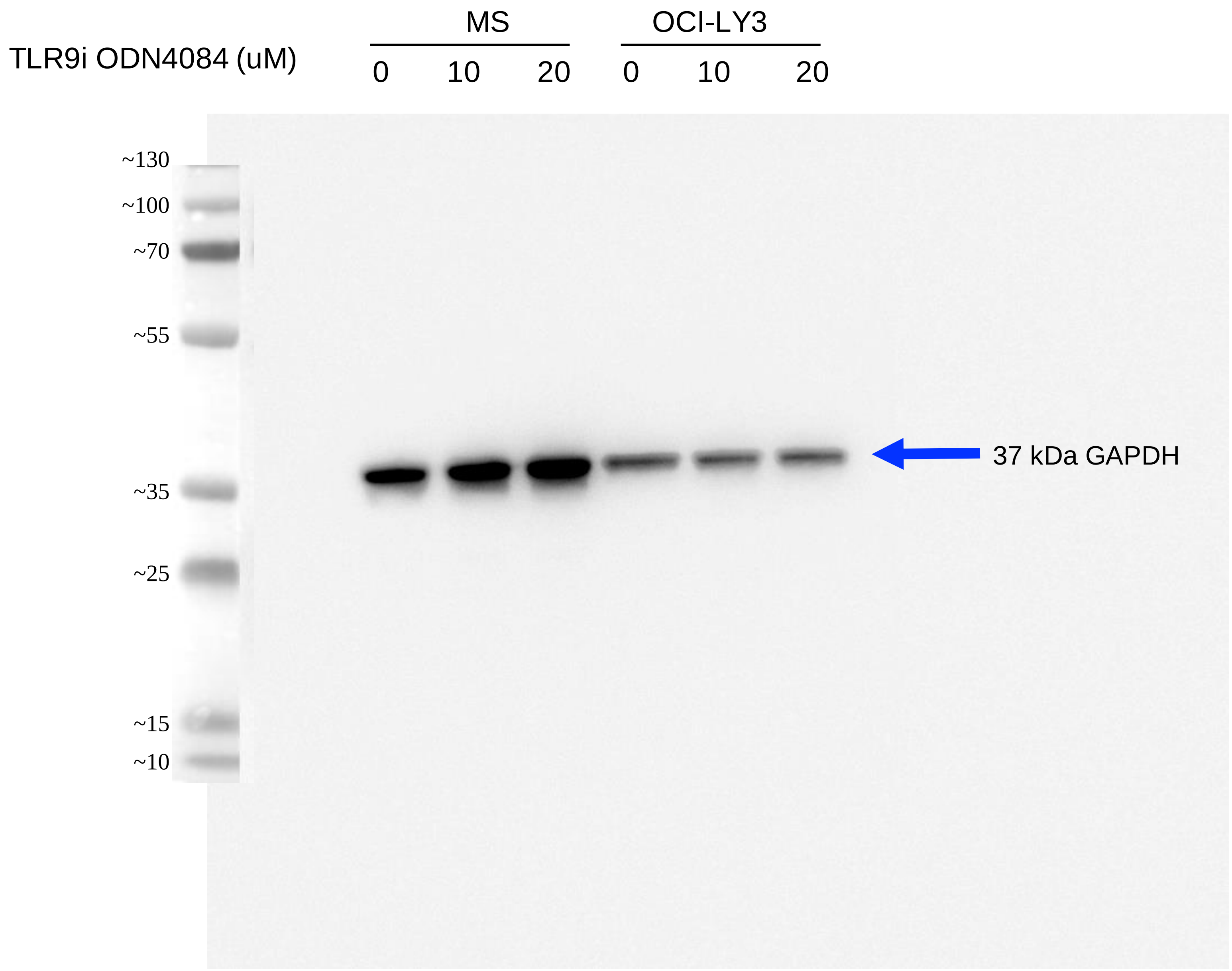

Figure S8

GAPDH (longer exp.)

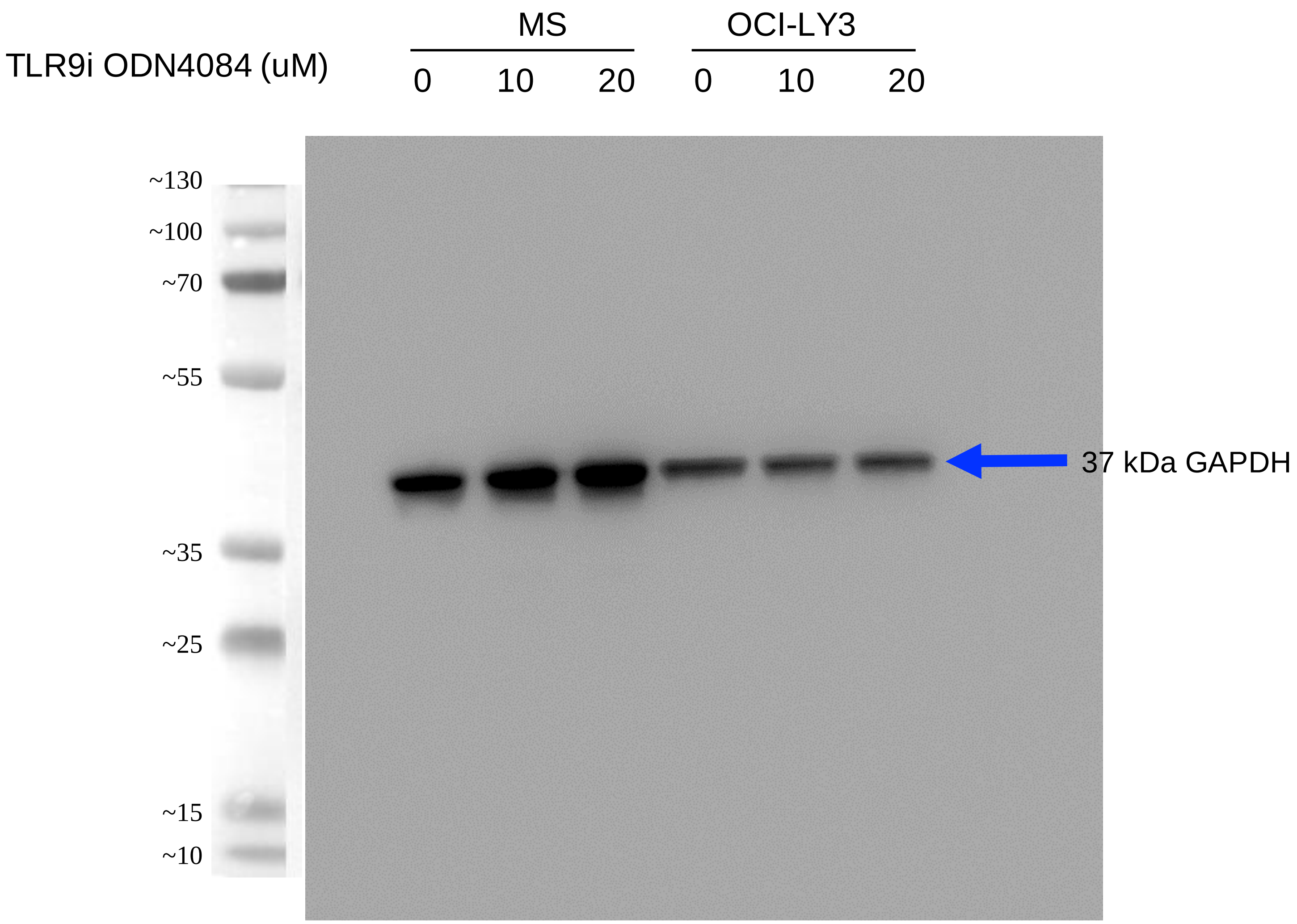

Figure S8

GAPDH

The crossed immunoblots refer to another project and Is not related to this paper

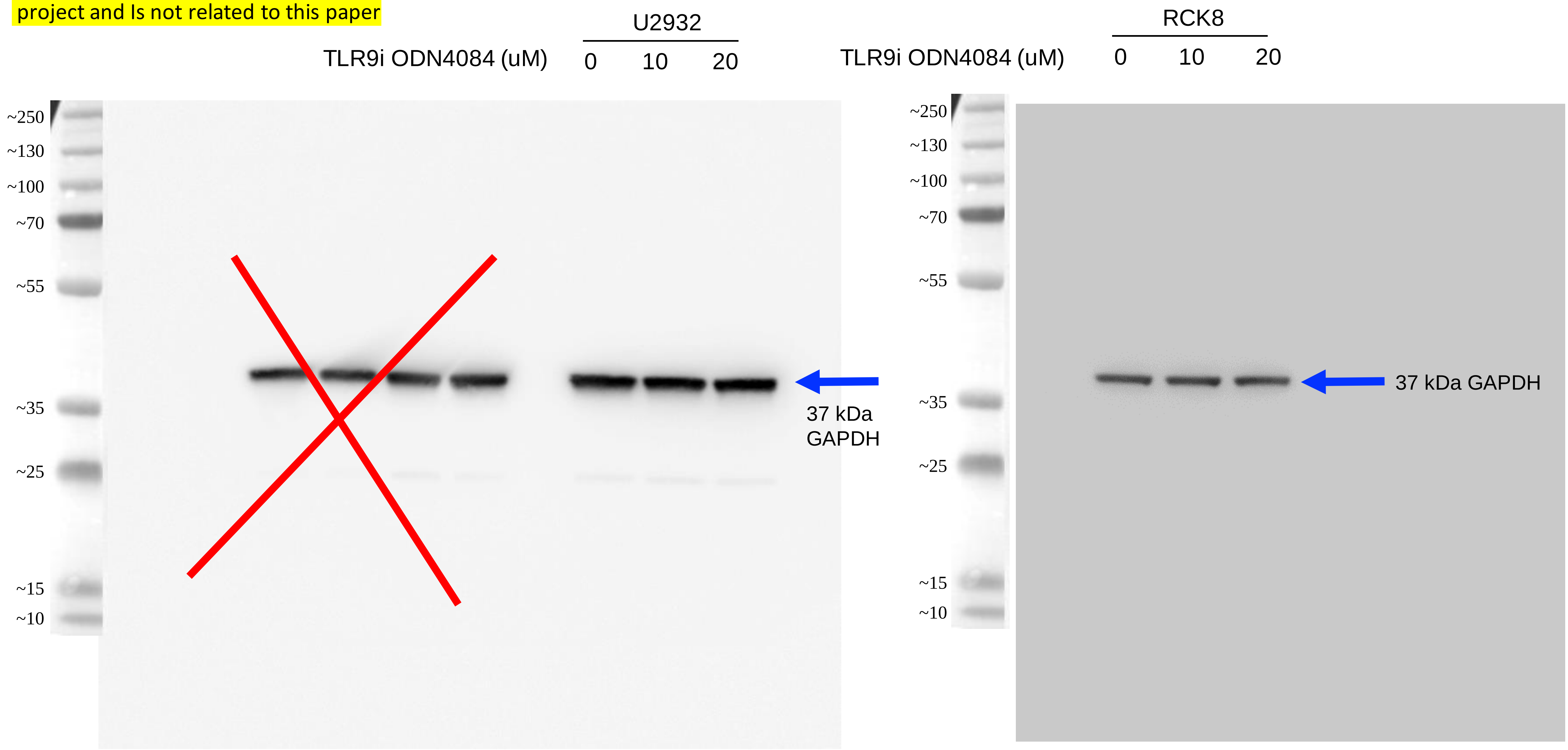

Figure S8

GAPDH

The crossed immunoblots refer to another project and Is not related to this paper

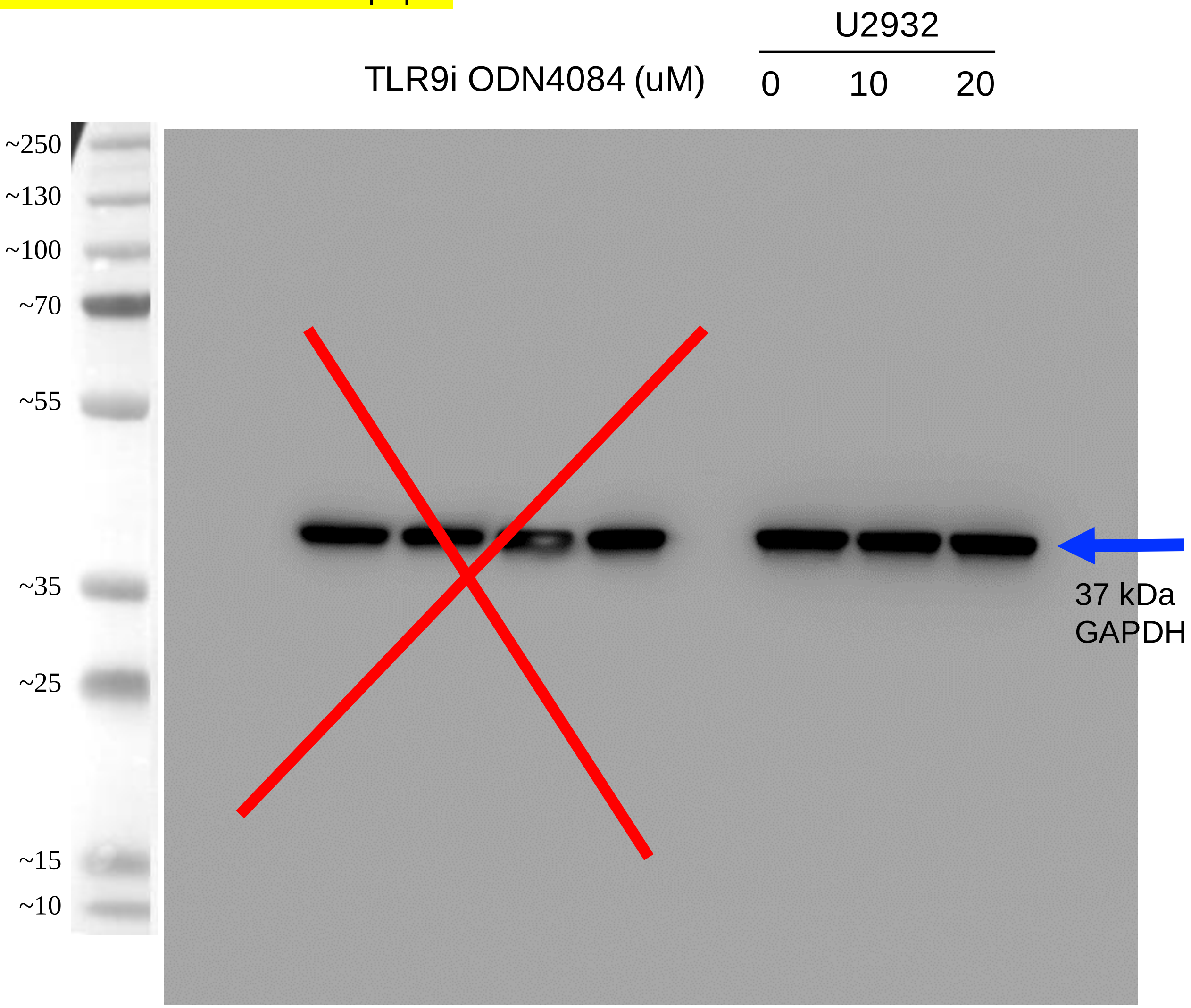

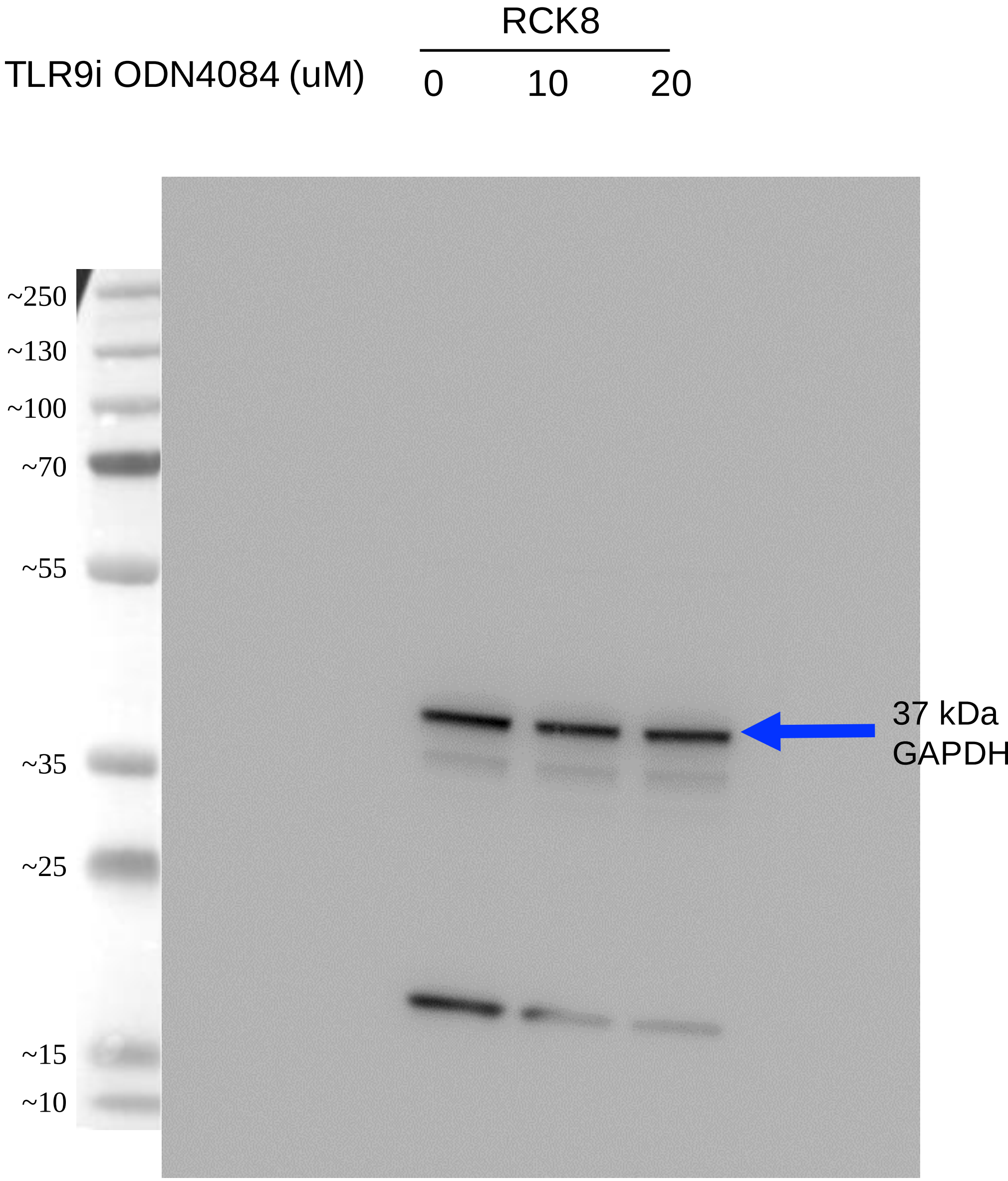

Supplement: Supplementary file 4 — Supplementary Material 4. [file 12885_2025_14359_MOESM4_ESM.zip › Raw data for Fig S8_REV4.pdf]
