# Supplementary figures and images for: Toll-like receptor 9 (TLR9) expression correlates with cell of origin and predicts clinical outcome in diffuse large B-cell lymphoma
Source: BMC Cancer. 2025 May 28;25:959. doi: 10.1186/s12885-025-14359-7 (PMC12117956; doi:10.1186/s12885-025-14359-7)

Figure 2

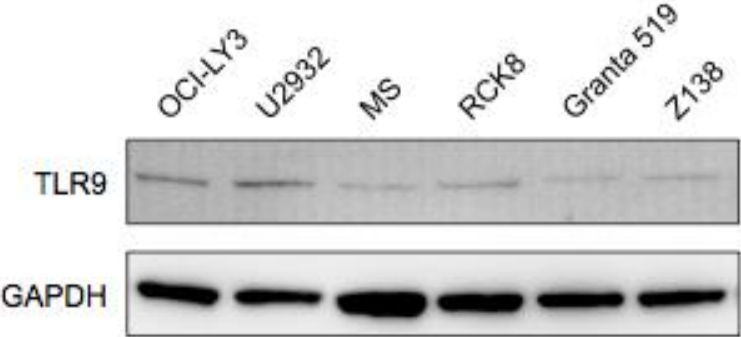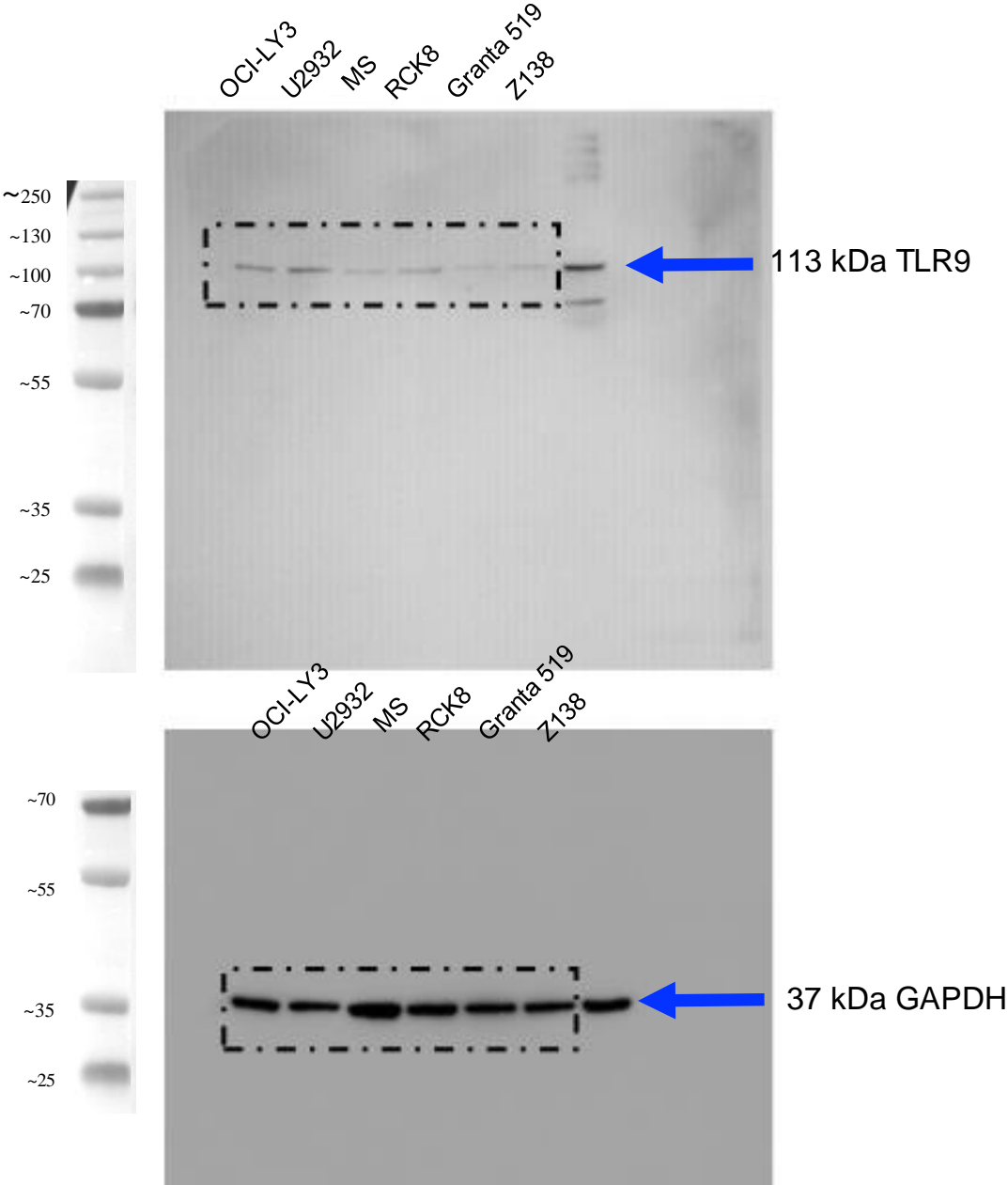

Supplement: Supplementary file 4 — Supplementary Material 4. [file 12885_2025_14359_MOESM4_ESM.zip › Raw data for Fig 2_REV4.pdf]
